# A randomized first-in-human phase I trial of differentially adjuvanted Pfs48/45 malaria vaccines in Burkinabé adults

**DOI:** 10.1172/JCI175707

**Published:** 2024-04-01

**Authors:** Alfred B. Tiono, Jordan L. Plieskatt, Alphonse Ouedraogo, Ben Idriss Soulama, Kazutoyo Miura, Edith C. Bougouma, Mohammad Naghizadeh, Aissata Barry, Jean Baptist B. Yaro, Sem Ezinmegnon, Noelie Henry, Ebenezer Addo Ofori, Bright Adu, Susheel K. Singh, Augustin Konkobo, Karin Lövgren Bengtsson, Amidou Diarra, Cecilia Carnrot, Jenny M. Reimer, Amidou Ouedraogo, Moussa Tienta, Carole A. Long, Issa N. Ouedraogo, Issaka Sagara, Sodiomon B. Sirima, Michael Theisen

**Affiliations:** 1 Groupe de Recherche Action en Santé (GRAS), Ouagadougou, Burkina Faso.; 2 Department for Congenital Disorders, Statens Serum Institut (SSI), Copenhagen, Denmark.; 3 Laboratory of Malaria and Vector Research, National Institute of Allergy and Infectious Diseases, National Institutes of Health, Rockville, Maryland, USA.; 4 Centre for Medical Parasitology at Department of Immunology and Microbiology, University of Copenhagen, Copenhagen, Denmark.; 5 Noguchi Memorial Institute for Medical Research, College of Health Sciences, University of Ghana, Legon, Accra, Ghana.; 6 Novavax AB, Uppsala, Sweden.; 7 Malaria Research and Training Center, Mali–National Institute of Allergy and Infectious Diseases International Center for Excellence in Research, University of Sciences, Techniques and Technologies of Bamako, Bamako, Mali.

**Keywords:** Infectious disease, Vaccines, Malaria

## Abstract

**BACKGROUND:**

Malaria transmission-blocking vaccines aim to interrupt the transmission of malaria from one person to another.

**METHODS:**

The candidates R0.6C and ProC6C share the 6C domain of the *Plasmodium falciparum* sexual-stage antigen Pfs48/45. R0.6C utilizes the glutamate-rich protein (GLURP) as a carrier, and ProC6C includes a second domain (Pfs230-Pro) and a short 36–amino acid circumsporozoite protein (CSP) sequence. Healthy adults (*n* = 125) from a malaria-endemic area of Burkina Faso were immunized with 3 intramuscular injections, 4 weeks apart, of 30 μg or 100 μg R0.6C or ProC6C each adsorbed to Alhydrogel (AlOH) adjuvant alone or in combination with Matrix-M (15 μg or 50 μg, respectively). The allocation was random and double-blind for this phase I trial.

**RESULTS:**

The vaccines were safe and well tolerated with no vaccine-related serious adverse events. A total of 7 adverse events, mild to moderate in intensity and considered possibly related to the study vaccines, were recorded. Vaccine-specific antibodies were highest in volunteers immunized with 100 μg ProC6C-AlOH with Matrix-M, and 13 of 20 (65%) individuals in the group showed greater than 80% transmission-reducing activity (TRA) when evaluated in the standard membrane feeding assay at 15 mg/mL IgG. In contrast, R0.6C induced sporadic TRA.

**CONCLUSION:**

All formulations were safe and well tolerated in a malaria-endemic area of Africa in healthy adults. The ProC6C-AlOH/Matrix-M vaccine elicited the highest levels of functional antibodies, meriting further investigation.

**TRIAL REGISTRATION:**

Pan-African Clinical Trials Registry (https://pactr.samrc.ac.za) PACTR202201848463189.

**FUNDING:**

The study was funded by the European and Developing Countries Clinical Trials Partnership (grant RIA2018SV-2311).

## Abstract 2

An in-human randomized clinical trial in Burkinabes found two malaria vaccines to be safe, tolerated, immunogenic, and functional against ProC6C.

## Introduction

*Plasmodium falciparum* malaria is transmitted by mosquitoes of the genus *Anopheles*. During a blood meal from an infected person, the female mosquito ingests sexual-stage parasites, which fertilize in the mosquito midgut to form zygotes and subsequently invasive sporozoites. When the mosquito takes another blood meal, these sporozoites are injected into the human host. Since transmission by mosquitoes is a biological bottleneck for malaria, measures to block transmission are integral components of malaria control strategies (1). Transmission-blocking vaccines (TBVs) aim to induce antisporogonic antibodies that disrupt parasite development in the mosquito, thereby halting transmission to another human (2). The Pfs48/45 antigen is an established *P*. *falciparum* TBV candidate (3). Pfs48/45 is expressed during gametocyte development in humans and on the surface of gametes (4), where it forms a protein complex with Pfs230 (5). The importance of Pfs48/45 in parasite fertilization is supported by the finding that male gametes lacking Pfs48/45 cannot bind to female gametes in the mosquito midgut, thereby preventing parasite development and ultimately invasive sporozoites (3). Immune-epidemiological studies conducted in malaria-endemic areas have demonstrated a high prevalence of naturally acquired antibodies against Pfs48/45 and Pfs230 (reviewed in ref. 7), suggesting that vaccine response might be modulated by preexisting immunity.

We have developed 2 vaccine antigens (R0.6C and ProC6C) based on the C-terminal 6-cysteine domain (6C) of Pfs48/45 (8, 9). This domain comprises the immune-dominant epitope I, which is the most effective target for transmission-blocking (TB) immunity (10, 11). Of the 2 vaccine designs, R0.6C contains the glutamate-rich protein (GLURP; “R0”) genetically coupled to 6C (9, 12), and ProC6C contains the Pfs230-Pro domain (“Pro”) joined to the 6C fragment through a short spacer sequence (“C”) derived from the circumsporozoite protein (CSP) major and minor repeats (8, 13). The CSP sequence was designed to distance the Pfs230 and Pfs48/45 domains to maintain conformation as well as to elicit anti-CSP titers with the potential to be functional (13). Immunization with either R0.6C or ProC6C has previously elicited functional antibodies in small rodents (8, 13, 14). Furthermore, the GLURP-R0 domain included in R0.6C may also elicit functional antibodies against the asexual blood stage (12, 15). Thus, both R0.6C and ProC6C may be regarded as multistage vaccines. The vaccine antigens were produced in *Lactococcus lactis* under current good manufacturing practice (13, 14, 16). Preclinical data led to the adoption of a dual-adjuvant design for subsequent clinical evaluation (8, 13, 14). While Alhydrogel (AlOH) is the most widely used adjuvant in human vaccines, it is also recognized that this adjuvant may not always promote potent humoral immunity. We have therefore developed a formulation strategy where the vaccine antigens are adsorbed on AlOH to enhance stability and uptake by antigen-presenting cells. To further enhance immunopotentiation and vaccine efficacy, the Matrix-M adjuvant was added at bedside, as this adjuvant enhanced very high levels of antibodies in humans when mixed with the R21 malaria vaccine antigen (17).

Here, we report results of the first-in-human phase I clinical trial, termed TBVax1, of 30 μg or 100 μg R0.6C or ProC6C adsorbed to Alhydrogel alone (R0.6C-AlOH and ProC6C-AlOH) or in combination with 15 μg or 50 μg Matrix-M adjuvant (R0.6C-AlOH/MM and ProC6C-AlOH/MM) in malaria-exposed adult volunteers in Burkina Faso. Importantly, our data suggest that the antibodies induced by the vaccines were functional, and the vaccines were found to be safe and well tolerated.

## Results

### Study population.

**Figure 1:**
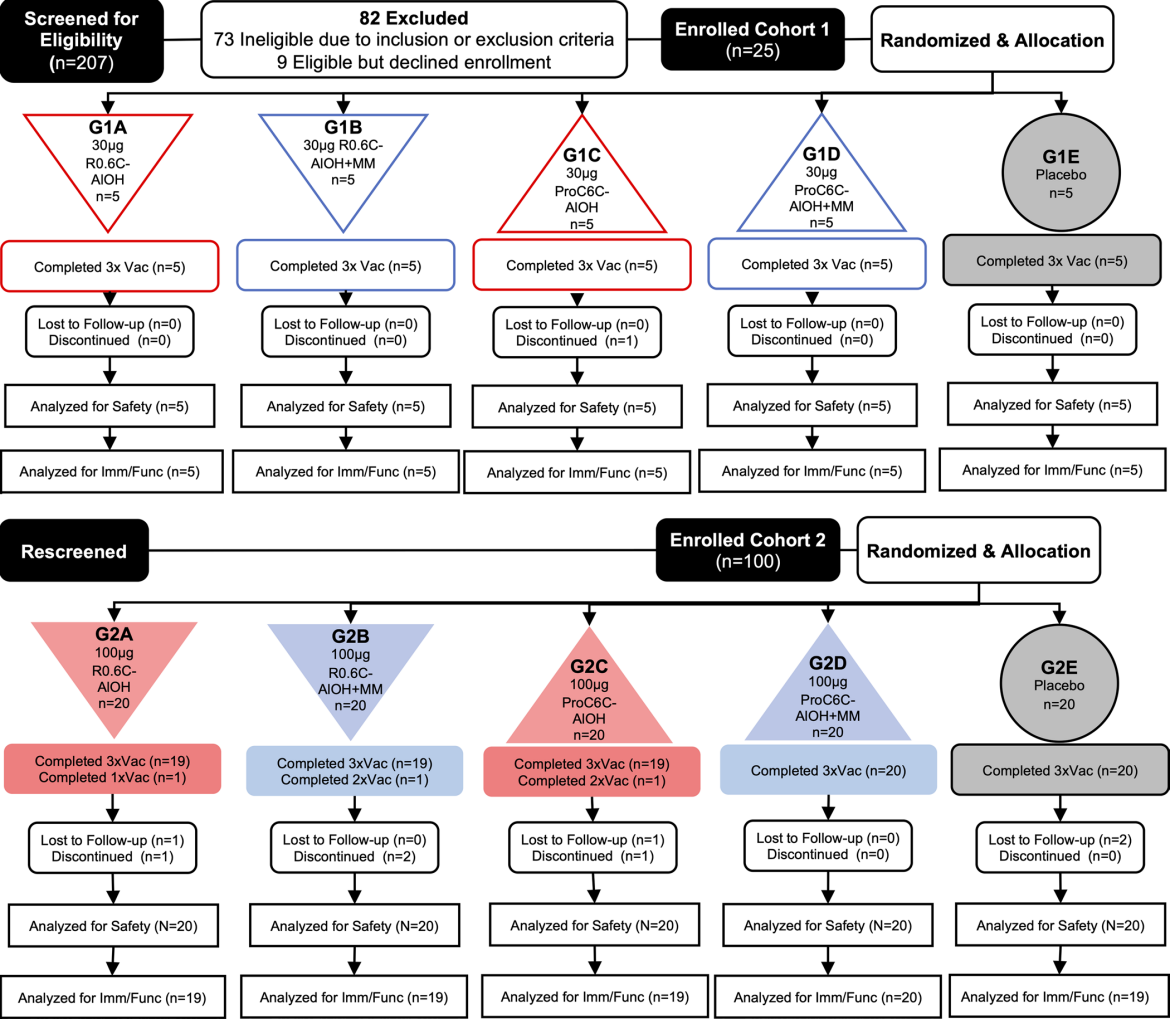
Study flow. Overview of trial flow for cohort 1 (low dose) and cohort 2 (high-dose) first-in-human trial of R0.C6-AlOH/MM and ProC6C-AlOH/MM. A total of 207 participants were screened. A total of 9 individuals did not complete the study, of whom 2 individuals withdrew their consent (groups 2A, 2C) and received only 1 and 2 vaccinations, respectively. An additional 2 migrated out the study area (groups 1C, 2B), and 4 individuals were reported as lost to follow-up (groups 2A, 2C, 2E, 2E). One individual (group 2B) tested positive for pregnancy and did not receive the third vaccine dose but has been followed up to the end of the study for safety reasons.

The phase I study was conducted in the Sabou health district in Burkina Faso and initiated on April 28, 2022. Two hundred seven (*n* = 207) volunteers were screened, and 25 were randomized to receive 3 injections 1 month (30 **±** 2 days) apart of either 30 μg of the study vaccines (R0.6C-AlOH or ProC6C-AlOH) with and without Matrix-M adjuvant (15 μg) or the Euvax B, hepatitis B (Hep B), control vaccine. Volunteers in this low-dose cohort 1 received their first injection starting on May 31, 2022, at the onset of the rainy season. After Data and Safety Monitoring Board (DSMB) review, volunteers not included in cohort 1 were rescreened to enroll 100 volunteers in cohort 2 (high dose), beginning on August 4, 2022, at the middle of peak transmission season. These volunteers were randomized to receive 3 injections of either 100 μg of the study vaccines (R0.6C-AlOH or ProC6C-AlOH) with and without Matrix-M adjuvant (50 μg) or the Euvax B vaccine. All volunteers completed their third vaccination for cohort 1 by September 9, 2022, and for cohort 2 by October 8, 2022. Demographics and other baseline characteristics were similar in all groups (Table 1). The study flow is summarized in Figure 1.

### Clinical and biological safety.

In general, the R0.6C-AlOH and ProC6C-AlOH vaccines (with or without the Matrix-M adjuvant) were well tolerated. No immediate reactogenicity (within the first 60 minutes after vaccination) was recorded in any of the vaccine groups. Local adverse events (AEs) were either mild or moderate (Tables 2 and 3), and resolved within 1 week without treatment. Pain/tenderness at the injection site was the most common AE in both cohort 1 (2 of the 60 doses; 3.3%) and cohort 2 (56 of the 240 doses; 23.3%). In cohort 2, vaccines containing Matrix-M adjuvant were more reactogenic than those without (22/120, 18.5%, vs. 34/120, 28.3%), while the difference was insignificant (*P* = 0.093, Fisher’s exact test). Systemic AEs were either mild or moderate and resolved within 9 days (Tables 2 and 3). Headache was the most frequent symptom in both low-dose (10 cases out of 16 events) and high-dose (12 cases out of 35) cohorts.

Overall, there were 7 AEs judged as being related to vaccination across all groups including the placebo group (Table 4). Headache was the most frequently reported AE to be related to the vaccination. All the AEs reported were mild to moderate in intensity. A detailed listing of unsolicited AEs is provided in Supplemental Table 1 (supplemental material available online with this article; https://doi.org/10.1172/JCI175707DS1). Overall, 118 unsolicited AEs were recorded, with 35 occurring in cohort 1 (low dose, *n* = 25) and 85 occurring in cohort 2 (high dose, *n* = 200) (Supplemental Table 1 and Supplemental Figure 1). The highest incidence of AEs was recorded within recipients of the low dose of ProC6C-AlOH, while the lowest incidence of AEs was recorded within recipients of the high dose of ProC6C-AlOH (Supplemental Figure 1).

### Immunogenicity of ProC6C and R0.6C formulations in malaria-exposed adults.

**Figure 2:**
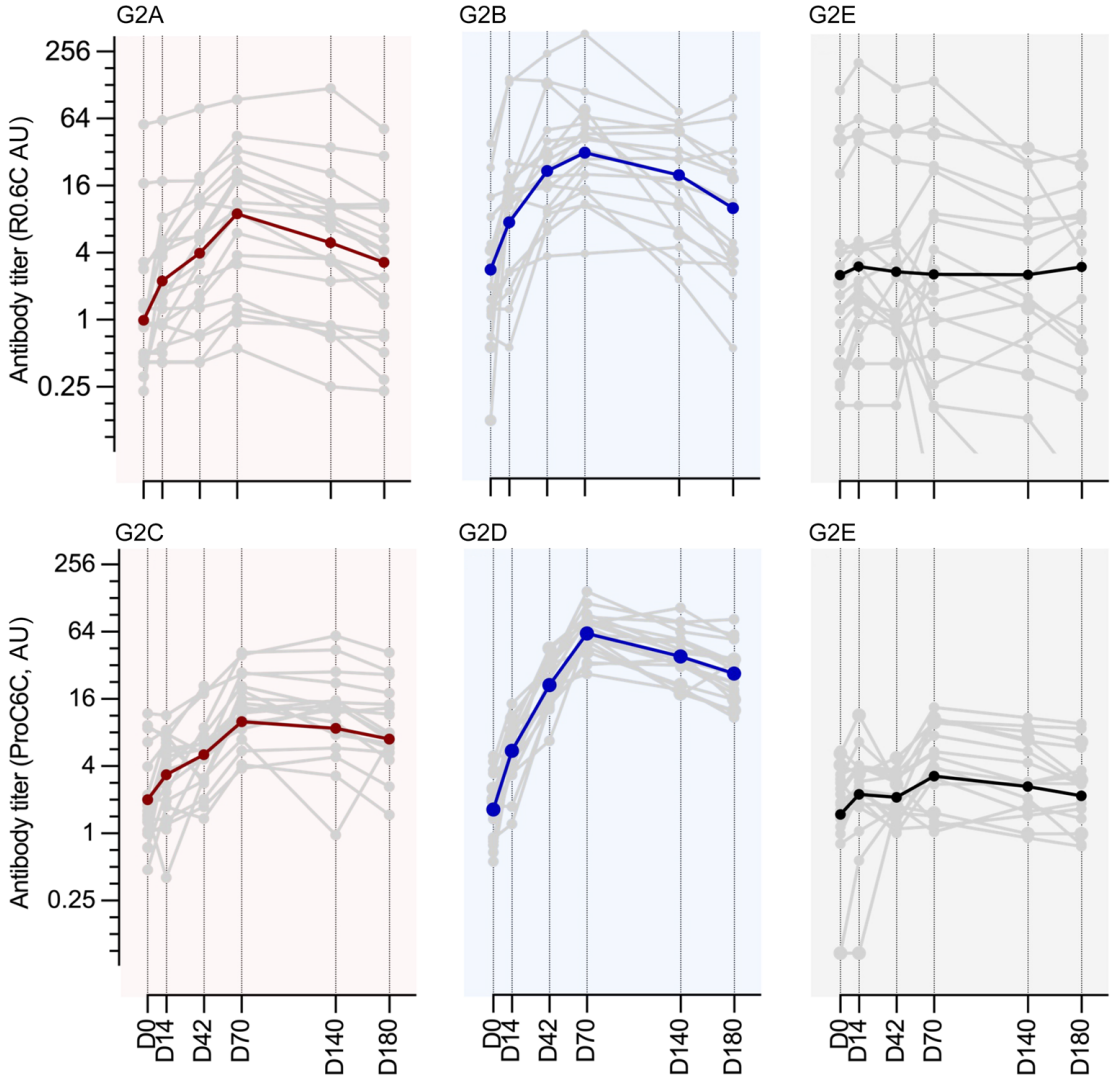
Vaccine-induced immunogenicity (cohort 2). The immunogenicity to the vaccine immunogens (R0.6C or ProC6C) was evaluated at each study time point (D0, D14, D42, D70, D140, and D180). Groups containing the AlOH adjuvant alone (geometric mean, red line) and AlOH/MM adjuvant (geometric mean, blue line) are plotted independently. Cohort 2 volunteers received 100 μg protein. The control group received Euvax B vaccine (Hep B, G2E) and was plotted to each vaccine immunogen (geometric mean, black line). Individual volunteers are plotted by gray lines for each group. GMT and fold increase/decrease are indicated in Tables 5 and 6, respectively. Vaccine-induced immunogenicity for cohort 1 (low dose) is provided in Supplemental Figure 2.

Vaccine-specific IgG responses were measured at baseline (day 0 [D0]), 2 weeks after each vaccination (D14, D42, D70), and 2 and 4 months after the last vaccination (D140 and D180) using the ELISA plates coated with corresponding immunogens (either R0.6C or ProC6C). The sera from control Euvax B vaccine groups (G1E and G2E) were tested against both R0.6C and ProC6C antigens. All volunteers in cohort 1 (Table 5 and Supplemental Figure 2) and cohort 2 (Figure 2 and Table 5) responded to vaccination by generating high levels of vaccine-specific antibodies. As expected for semi-immune individuals, antigen-specific IgGs against R0.6C and ProC6C were present before vaccination. The geometric mean titer (GMT) ranged from 0.9 to 3.8, and there were insignificant differences among different groups at baseline (*P* = 0.744 for R0.6C titers and *P* = 0.630 for ProC6C titers by 1-way ANOVA using log-transformed ELISA titers). At D70, R0.6C GMT increased to 8.0 (95% confidence interval [CI], 1.5 to 41.9), 41.4 (19.1 to 89.7), 7.3 (3.6 to 14.7), and 30.0 (18.5 to 48.6) for groups G1A (30 μg R0.6C-AlOH), G1B (30 μg R0.6C-AlOH/MM), G2A (100 μg R0.6C-AlOH), and G2B (100 μg R0.6C-AlOH/MM), respectively (Table 5). On the other hand, the GMT in the control groups stayed at similar levels: 1.4 (0.1 to 23.5) for G1E and 2.5 (0.8 to 7.7) for G2E (Table 5). There were significant differences among different groups (*P* < 0.001, 1-way ANOVA), and Matrix-M adjuvant groups showed significantly higher titers than the controls at both low and high doses (*P* = 0.023 for G1B vs. G1E and *P* < 0.001 for G2B vs. G2E by Tukey’s multiple-comparison test). On the contrary, insignificant differences were observed between AlOH adjuvant groups and control groups (*P* = 0.563 for G1A vs. G1E and *P* = 0.368 for G2A vs. G2E). There was no significant dose effect for the same vaccine formulations (*P* > 0.999 for G1A vs. G2A and *P* = 0.999 for G1B vs. G2B). Similar patterns of anti-ProC6C IgG responses were observed. At D70, ProC6C GMTs were 7.3 (3.3 to 16.4), 46.2 (22.4 to 95.5), 10.0 (5.2 to 19.1), and 61.8 (50.7 to 75.5) for groups G1C (30 μg ProC6C-AlOH), G1D (30 μg ProC6C-AlOH/MM), G2C (100 μg ProC6C-AlOH), and G2D (100 μg ProC6C-AlOH/MM), respectively, while the GMTs in the control groups were 2.0 (0.3 to 12.1) for G1E and 3.3 (1.8 to 5.9) for G2E (Table 5). There were significant differences among different groups (*P* < 0.001), and Matrix-M adjuvant groups again showed significantly higher titers than the controls (*P* < 0.001 both for G1D vs. G1E and G2D vs. G2E). In the case of ProC6C, the difference between G2C and G2E also reached significance (*P* = 0.019), while the difference between G1C and G1E was insignificant (*P* = 0.371). Similarly to the R0.6C data, the dose effect within the same vaccine formulation did not reach significance (*P* = 0.991 for G1C vs. G2C and *P* = 0.994 for G1D vs. G2D).

Since anti-R0.6C and anti-ProC6C titers cannot be compared directly, a fold increase in titers from D0 and D70 was calculated individually, then compared among different groups (Table 6). There were large variations in fold increase among individuals (ranging from 1.1- to 487.0-fold); therefore, the statistical comparisons were performed only within high-dose groups (*n* = 19–20 per group), not for low-dose groups (*n* = 5 per group). The mean fold increases in G2A, G2B, G2C, and G2D were 11.9 (95% CI, 4.2 to 19.6), 25.7 (8.0 to 43.4), 9.8 (3.8 to 15.8), and 49.2 (27.6 to 70.8), respectively. There were significant differences among different groups (*P* < 0.001), and the fold increase in G2D was significantly higher than those in G2A (*P* = 0.002) and in G2C (*P* = 0.001). The differences for the other group comparisons were insignificant.

As expected, GMT went down over time after D70 for all R0.6C- or ProC6C-vaccinated groups (Table 5). At D180, R0.6C GMTs decreased to 4.5 (0.9 to 21.6), 9.9 (3.1 to 31.5), 3.0 (1.4 to 6.1), and 8.1 (4.0 to 16.5) for groups G1A, G1B, G2A, and G2B, respectively, and the GMTs in the control groups were 1.9 (0.1 to 28.2) for G1E and 1.2 (0.3 to 4.4) for G2E. Only the G2B group showed significantly higher titers than the control G2E group (*P* = 0.034). ProC6C GMTs were 3.8 (1.8 to 8.1), 19.1 (9.0 to 40.6), 7.0 (3.7 to 13.4), and 26.9 (20.6 to 35.2) for G1C, G1D, G2C, and G2D, respectively, at D180, and the GMTs were 2.0 (0.3 to 12.8) for G1E and 2.2 (1.2 to 3.9) for G2E control groups. Except for G1C (*P* = 0.932), the other groups maintained significantly higher titers than the controls (*P* = 0.015 for G1D, *P* = 0.016 for G2C, and *P* < 0.001 for G2D). When fold decreases from D70 to D180 were calculated for the high-dose groups, mean fold decreases in G2A, G2B, G2C, and G2D were 2.7 (2.1 to 3.4), 5.7 (1.9 to 9.5), 1.5 (1.2 to 1.9), and 2.5 (2.0 to 3.0), respectively. The fold decrease in G2B was significantly higher than those in G2C (*P* = 0.006) and in G2D (*P* = 0.047), but no other significant differences were observed.

### Antibody responses against R0.6C and ProC6C constituent antigens.

**Figure 3:**
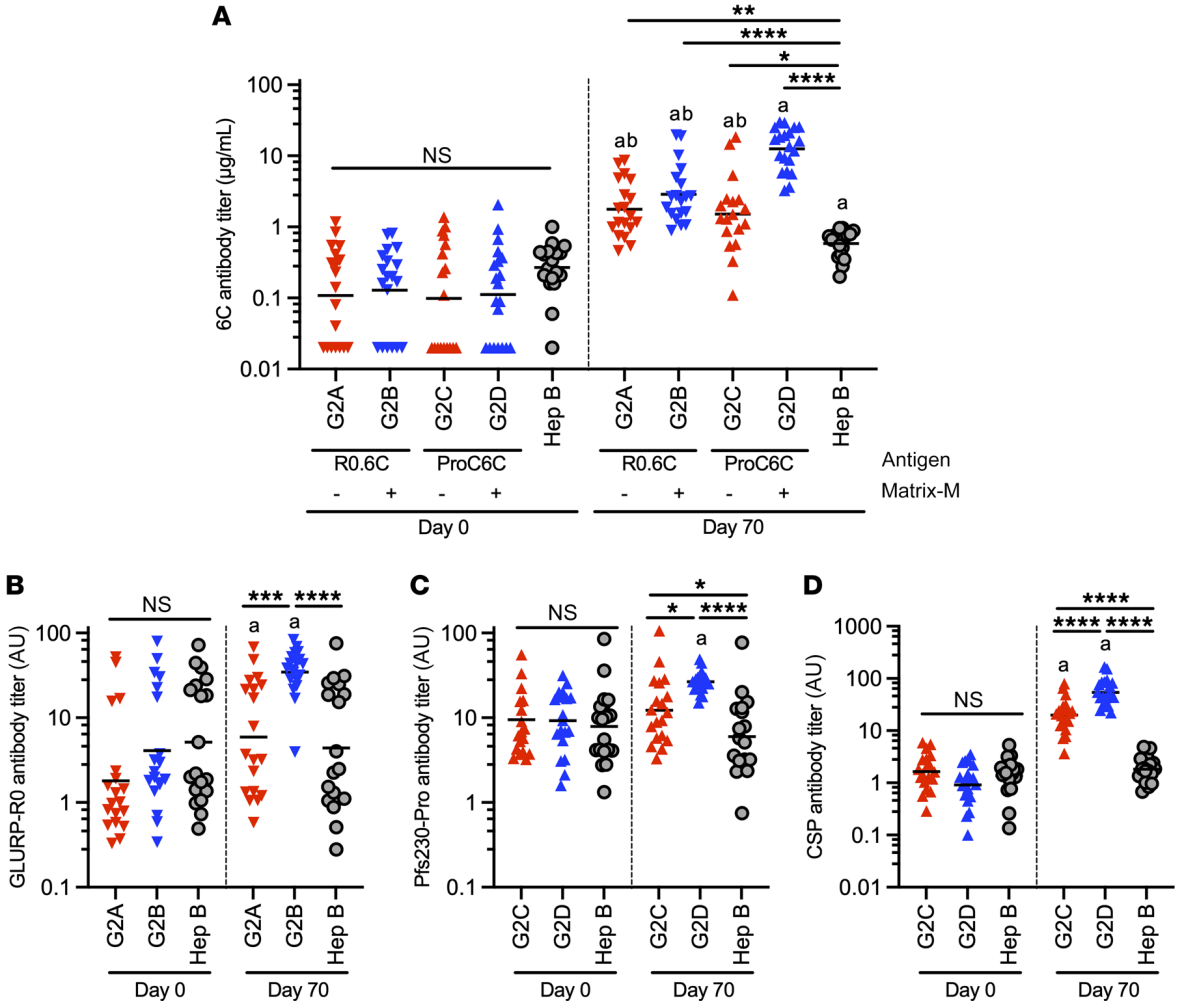
IgG levels against the vaccine constituent antigens. IgG antibody levels against Pfs48/45-6C (**A**), GLURP-R0 (**B**), Pfs230-Pro (**C**), and CSP (**D**) at baseline (D0) and 14 days after last vaccination (D70). Each symbol represents a sample; the horizontal line represents the geometric mean. Data are shown for populations receiving either R0.6C, ProC6C, or hepatitis vaccine as indicated. Antibody levels are given as TB31F equivalence (μg/mL) for Pfs48/45 IgG and in arbitrary units (AU) for GLURP-R0, CSP, and Pfs230-Pro IgG. ^a^Significantly higher than D0 by paired *t* test. ^b^Significantly lower than G2D (*P* < 0.0001) by Tukey’s multiple-comparison test. NS, not significant by 1-way ANOVA test. *P < 0.05, **P < 0.01, ***P < 0.001, ****P < 0.0001 by Tukey’s multiple-comparison test.

Antibody responses against the R0.6C and ProC6C constituent antigens, including the common Pfs48/45-6C domain, were measured on D0 and D70 in the respective R0.6C and ProC6C vaccine groups. As expected, the majority of individuals had a detectable level of antibodies against each of constituent antigens at the baseline (D0), while there was no significant difference among groups (*P* > 0.1 by 1-way ANOVA using log-transformed ELISA titers; Figure 3). R0.6C and ProC6C vaccinations elicited a significant increase in geometric mean Pfs48/45-6C IgG levels (*P* < 0.0001, paired *t* test; Figure 3A). An increase, significant but of low magnitude, was also observed in the Hep B control group (*P* < 0.0001, paired *t* test; Figure 3A) and was likely the result of natural exposure. At D70 there was a significant difference among study groups (*P* < 0.0001, ANOVA; Figure 3) with ProC6C-AlOH/MM (G2D) having a higher geometric mean Pfs48/45-6C IgG concentration compared with all other groups. For the other constituent antigens, there were significant increases from D0 to D70 for all antigens in all R0.6C and ProC6C groups (*P* < 0.0001, paired *t* test; Figure 3, B–D), except for Pfs230-Pro IgG in the ProC6C-AlOH (G2C) group. At D70, all vaccine groups showed higher titers compared with Hep B, except for GLURP-R0 IgG level in R0.6C-AlOH (G2A).

### Vaccine antibody responses in relation to natural P. falciparum exposure.

Asexual- and sexual-stage parasite densities were determined in blood throughout the study at each study visit (Supplemental Figures 3 and 4). All baseline infections were asymptomatic, with no fever or any other symptom of malaria. There were no significant differences of anti-R0.6C and anti-ProC6C titers at baseline (D0) or D70 with respect to parasite presence at the respective time point (Supplemental Figure 3). Next, to determine whether concurrent malaria infections impacted the magnitude of D70 antibody, we counted the number of visits (a total of 6 visits from D0 to D70) at which an individual tested parasite positive. There was an average of 1.27 positive visits per person (Supplemental Figure 4A), and there was no difference in number of positive visits across the 4 study groups receiving the malaria vaccines (*P* = 0.3383, χ^2^ test). The fold increase (D70/D0) in anti-R0.6C and anti-ProC6C antibody responses was not affected by the number of parasite-positive visits (*P* > 0.5 by Spearman’s rank test; Supplemental Figure 4B).

Finally, we investigated the impact of natural malaria exposure measured as baseline antibody levels (D0) on the magnitude of D70 antibody levels using Spearman’s rank correlation. For R0.6C-immunized individuals we found a significant positive correlation between D0 and D70 anti-R0.6C IgG responses (*P* = 0.0169) in the AlOH group (G2A) and anti-GLURP IgG responses (*P* = 0.0327 and 0.0081) in the AlOH and AlOH/MM (G2A and G2B) groups, respectively (Supplemental Figure 5A). For ProC6C-immunized volunteers, baseline antibody levels had a significant impact on Pfs230-Pro (*P* = 0.0002) and CSP (*P* = 0.0001) D70 antibody levels in the AlOH group (G2C), while there was no similar effect in the Matrix-M group (G2D) (Supplemental Figure 5B).

### Functional activity of sera in the standard membrane feeding assay.

**Figure 4:**
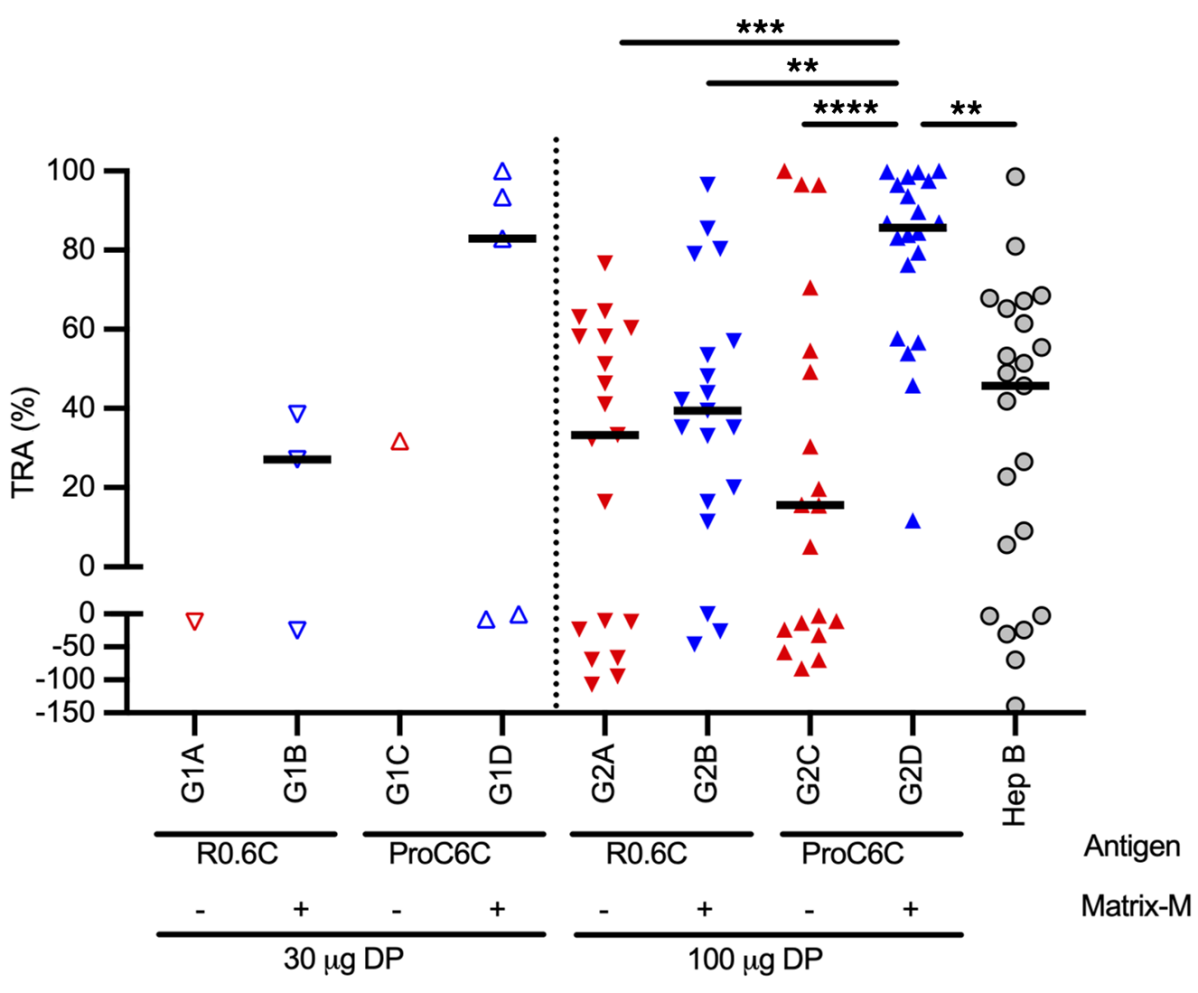
Biological activity of antibodies. The biological activity (functionality) for each group is plotted for each volunteer at D70 from purified IgG at 15 mg/mL in the SMFA (with human complement) as transmission-reducing activity (TRA). Red symbols, groups receiving AlOH adjuvant alone; blue symbols, groups receiving AlOH/MM adjuvant. Control groups receiving Euvax B vaccine (Hep B, G1E and G2E) are combined and reported (gray circles). Cohort 1 (low dose, 30 μg protein) is indicated by open symbols and cohort 2 (high dose, 100 μg protein) by filled symbols. The median for each group is indicated by a line. DP, drug product (either R0.6C-AlOH or ProC6C-AlOH). Statistical significance is indicated among high-dose and control groups by Kruskal-Wallis test with a Dunn-Bonferroni adjustment for multiple comparisons; **P < 0.01, ***P < 0.001, ****P < 0.0001.

The standard membrane feeding assay (SMFA), which is the gold standard for assessing transmission blockage (10, 18, 19), was used to assess the functionality of antibodies elicited after vaccination. IgG was purified from D70 serum samples from 121 individuals representing all vaccine groups that completed the trial. Each individual data point represents percentage inhibition in oocyst density (transmission-reducing activity [TRA]) with purified IgG (15 mg/mL) from a single individual volunteer (Figure 4). The 15 mg/mL test concentration was chosen as it is the average physiological concentration of total IgGs in African American adults (20). When all purified IgGs from the high-dose cohort (100 μg) were tested, 13 of 20 individuals had high TRA (>80% TRA) and 5 others had substantial TRA (54%–79% TRA) in the ProC6C-AlOH/MM group (G2D). The level of TRA in the ProC6C-AlOH/MM group was significantly higher than those in the other 3 high-dose groups (*P* = 0.0002 to R0.6C-AlOH, *P* = 0.0051 to R0.6C-AlOH/MM, and *P* = 0.0001 to ProC6C-AlOH) and that in the control group (Hep B, *P* = 0.0015). In contrast, there were no significant differences among the other 3 high-dose groups and the control group (*P* > 0.9999 for all comparisons). For the low-dose cohort (30 μg), while all 5 IgGs from the ProC6C-AlOH/MM group (G1D) were tested by SMFA, only randomly selected samples (1 to 3 out of 5 per group) from the other low-dose groups were tested by SMFA. Three of five IgGs in the 30 μg ProC6C-AlOH/MM group showed >80% TRA. Because of the small sample size, statistical analysis was not performed in the low-dose groups.

**Figure 5:**
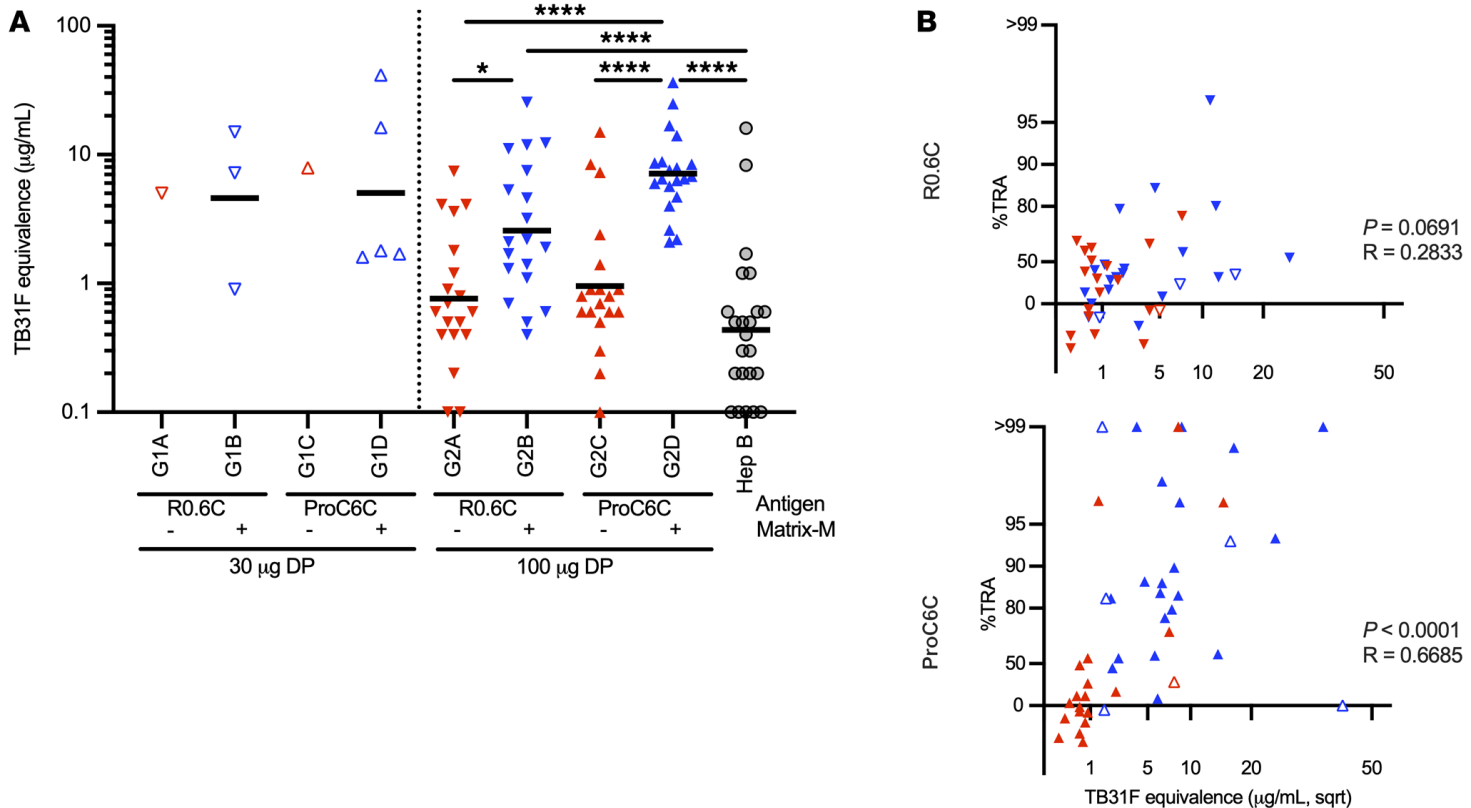
Biological and antibody correlation. Purified IgG used above for analysis in the SMFA was also evaluated in ELISA to the Pfs48/45-6C. (**A**) Arbitrary units (AU) for 6C titers are reported for D70 IgGs using the Pfs48/45 mAb TB31F as a standard. Red symbols, groups receiving AlOH adjuvant alone; blue symbols, groups receiving AlOH/MM adjuvant. Cohort 1 (low dose, 30 μg protein) is indicated by open symbols and cohort 2 (high dose, 100 μg protein) by filled symbols. Control groups receiving Euvax B vaccine (Hep B, G1E and G2E) are combined and reported (gray circles). The median for each group is indicated by a line. Statistical significance is indicated between groups by Kruskal-Wallis test with a Dunn-Bonferroni adjustment for multiple comparisons; *P < 0.05, ****P < 0.0001. (**B**) The biological activity is plotted in log of mean oocyst ratio (LMR) between control and test IgGs (*y* axis) respective to square root (sqrt) of D70 antibody levels of Pfs48/45-6C (*x* axis). For ease of comprehension, the *y* axis shows corresponding percent TRA values instead of LMR. The R0.6C groups (all individuals) are plotted in the top panel and ProC6C groups (all individuals) in the bottom panel using the same symbols as in **A**. The Spearman’s rank *P* value and correlation coefficient (*R*) for all individuals in each panel are shown. Antibody correlation for Pfs230-Pro is provided in Supplemental Figure 5.

Next, we measured specific antibodies against the Pfs48/45-6C domain in the purified IgGs used for functional SMFA activity assessment. The GMT of anti-6C IgG concentration ranged from 0.4 to 7.9 μg/mL, and there were marked differences between groups (Figure 5A). The GMT was 2.5 μg/mL (95% CI, 1.4 to 4.3), 7.1 μg/mL (95% CI, 5.1 to 10.0), and 0.4 μg/mL (95% CI, 0.2 to 0.8) in the R0.6C-AlOH/MM (G2B), ProC6C-AlOH/MM (G2D), and Hep B control (G2E) groups, respectively. In this analysis, the 100 μg ProC6C-AlOH/MM formulation elicited significantly higher titers compared with R0.6C-AlOH (*P* < 0.0001), R0.6C-AlOH/MM (*P* < 0.0001), and ProC6C-AlOH (*P* = 0.0020) and the Hep B comparator (*P* < 0.0001).

For ProC6C-immunized individuals, levels of Pfs48/45-6C–specific antibodies significantly correlated (*P* < 0.0001, Spearman’s coefficient *R* = 0.6685) with the TRA, suggesting the notion that the functional activity was due to vaccine-specific antibodies (Figure 5B). On the other hand, there was no significant correlation (*P* = 0.0688) between Pfs48/45-6C responses and TRA in R0.6C-immunized individuals (Figure 5B). Similarly, Pfs230-Pro–specific antibodies were significantly correlated to TRA in ProC6C-immunized volunteers (*P* = 0.0165, Spearman’s coefficient *R* = 0.3557) but not in those immunized with R0.6C (Supplemental Figure 6), as the immunogen is not present in R0.6C. Thus, ProC6C is effective in eliciting functional transmission-blocking antibodies, including those against Pfs48/45-6C and the Pfs230-Pro domain, in malaria-exposed individuals.

**Figure 6:**
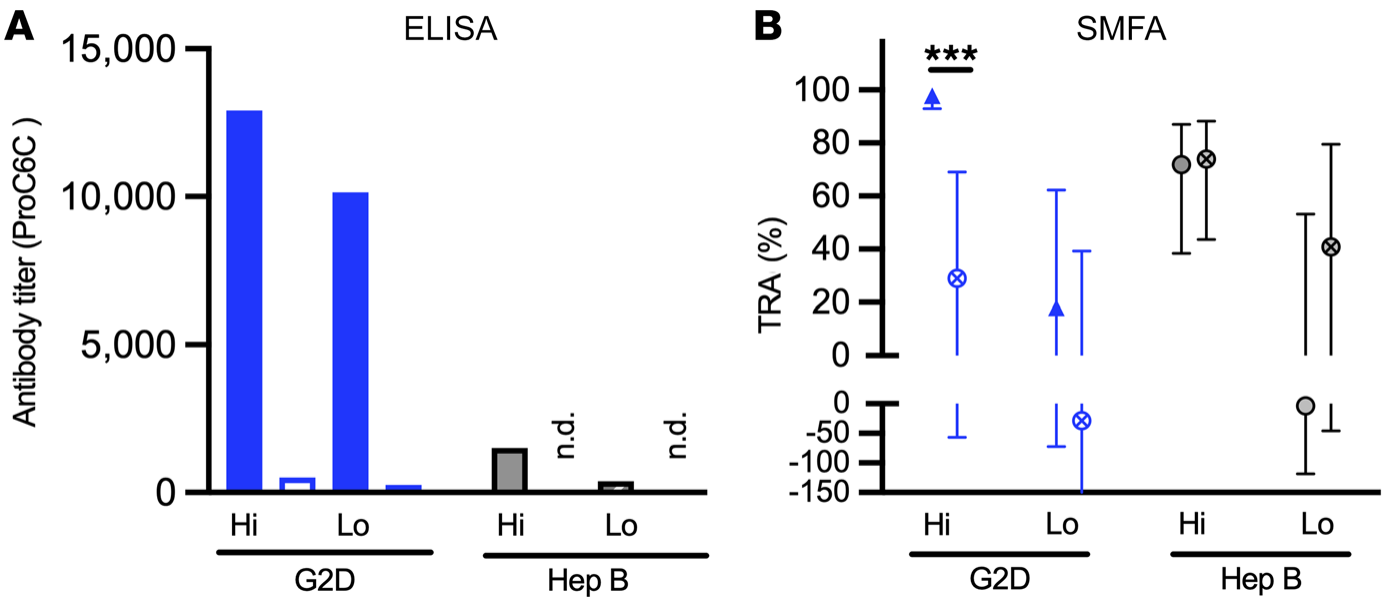
ProC6C-specific antibodies were depleted from pooled IgG. From G2D (100 μg ProC6C-AlOH/MM) and Hep B (G2E) groups, 2 pooled IgGs per group were generated based on individual TRA level. G2D-hi pooled IgG contained individual IgGs that showed >95 TRA (*n* = 6), G2D-lo with <80 TRA (*n* = 5), Hep B–hi with >80 TRA (*n* = 2), and Hep B–lo with <50 TRA (*n* = 5). From the 4 original pooled IgGs, anti-ProC6C-specific antibodies were depleted. (**A**) Antibody titers (ELISA units) of the original (filled bars) and depleted (open bars) IgGs were determined by ELISA. “ND” indicates that ELISA units were too low to be determined for the depleted IgGs. (**B**) All IgGs were tested at 15 mg/mL by SMFA. Observed TRA (circles) and the 95% CI (error bars) are shown. The 95% CI and statistical significance (***P < 0.001) were determined by the assay-specific zero-inflated negative binomial model (19).

To determine the relative role of vaccine-induced antibodies for the TRA observed with IgG from volunteers vaccinated with 100 μg ProC6C-AlOH/MM (G2D), vaccine-specific antibodies were depleted from vaccinated volunteers (G2D) and Hep B controls (G2E), and the resulting IgG preparations were tested in the SMFA. For the depletions, 4 pools were generated based on the reactivity of the individual IgGs: (a) G2D-hi (*n* = 6, >95% TRA), (b) G2D-lo (*n* = 5, <80% TRA), (c) Hep B–hi (*n* = 2, >80% TRA), and (d) Hep B–lo (*n* = 5, <50% TRA). Anti-ProC6C-specific antibodies were efficiently depleted, as each post-depletion pool showed less than 5% of anti-ProC6C antibody level compared with the pre-depletion pool as judged by ELISA (Figure 6A). When the pre- and post-depletion IgGs were tested in the SMFA, we found that G2D-Hi IgG lost activity after the depletion (changed from 98% to 29% TRA), and the difference was significant (*P* < 0.001) (Figure 6B). On the other hand, Hep B–hi IgG maintained similar activity before (72% TRA) and after (74% TRA) depletion (*P* > 0.999). As expected, the G2D-lo and Hep B–lo IgGs showed no marked inhibition in the pre- and post-depletion pools. Taken together, these results provide strong support for the idea that anti-ProC6C antibodies induced by the vaccination led to higher functional activity in the G2D group over the preexisting immunity induced by natural infections.

## Discussion

In this first-in-human clinical trial of two TBV candidates, we tested an expression platform that allows the production of chimeric vaccines based on antigens from different developmental stages of *P*. *falciparum*. Two chimeric proteins (R0.6C and ProC6C) were formulated on aluminum hydroxide (Alhydrogel [AlOH] adjuvant) and evaluated with and without the saponin-based adjuvant Matrix-M (21). We found that both the ProC6C and R0.6C vaccines were well tolerated and induced IgG antibodies against the vaccine antigens and their respective constituent antigens. Antibody responses peaked 2 weeks after the third vaccination. A strength of the present study is that two TBV candidates, with two adjuvants, were evaluated in a single randomized, comparator-controlled clinical trial in the target population — malaria-exposed individuals. Overall, 97 of 100 (97%) of the participants received all 3 vaccinations. Both vaccine formulations showed an acceptable safety profile in this population of Burkinabé adults. There were no serious adverse events, unexpected reactions, or safety concerns considered to be related to the vaccines during the course of the trial.

An important outcome of the present study is the finding that the immune-stimulatory activity of Matrix-M adjuvant was much stronger in humans than previously observed in mice (8, 9, 13, 14, 22, 23). This confirms prior findings that preclinical models are not always indicative of a human response (24) and supports the evaluation of multiple adjuvants in humans during vaccine development. We found that the Matrix-M adjuvant with AlOH enhanced peak antibody responses 4.1-fold (*P* = 0.032) and 6.2-fold (*P* < 0.001) compared with the AlOH adjuvant alone in volunteers immunized with 100 μg of R0.6C and ProC6C, respectively, supporting the immune-stimulating activity of the Matrix-M adjuvant (21). It is believed that the Matrix-M adjuvant recruits immune cells (mainly dendritic cells, monocytes, and neutrophils) to the site of injection and that it facilitates drainages of antigen and immune cells to the draining lymph nodes (discussed in detail in ref. 21). We propose that AlOH serves as a vehicle providing depot formation at the site of injection and/or provides a multimeric presentation of the antigen, thereby enhancing the immune-potentiating effects of the Matrix-M adjuvant. The potent adjuvanticity of Matrix-M was not unexpected, as other malaria vaccines formulated with this adjuvant induced high levels of protective antibodies in humans (17, 25). However, the analysis reported herein extensively expands these observations by comparing 2 different vaccine antigens and by using Matrix-M as an immune modulator supplement to vaccines formulated on AlOH. The stability of the AlOH drug products with Matrix-M was supported by our unpublished observations, demonstrating stability for at least 24 hours at room temperature, and a detailed Pharmacy Manual (see supplemental material) developed for their handling.

Importantly, this side-by-side comparative study demonstrated that IgG from 13 of 20 individuals immunized with 100 μg ProC6C-AlOH/MM (and 3 of 5 in the low-dose group) reduced transmission of parasites to mosquitoes by more than 80% in ex vivo SMFA at physiological antibody concentrations (15 mg/mL), and the TRA level was significantly higher than that in the control group. In contrast, there was no significant difference between any of the other vaccine formulations and the control group. As expected in a phase I study conducted in an endemic population, IgG from the Hep B control group also showed SMFA activities with a median TRA of 46%. This TRA is most likely induced by naturally occurring malarial IgG antibodies and was similar to that observed in the comparator group of a phase I trial conducted in Malian adults (26). Furthermore, due to the nature of the SMFA, there is greater uncertainty regarding low TRA values such as those observed in the Hep B group (19, 27). Considering these inherent challenges with the SMFA and the fact that the depletion of ProC6C-specific IgG almost completely abolished the SMFA activity, our results strongly suggest that vaccine-induced anti-ProC6C IgG antibodies were responsible for the higher TRA level in the 100 μg ProC6C-AlOH/MM group.

Since the two TBVs share the Pfs48/45-6C domain, specific antibody responses were also measured against the 6C domain, allowing for a direct comparison of vaccine-specific Pfs48/45 antibody responses in the D0 and D70 sera and the IgG samples tested by SMFA. We found that within the high-dose cohort, ProC6C was superior in generating Pfs48/45-specific antibodies and that the Matrix-M formulations elicited the highest levels of specific antibodies, presumably explaining why IgG from these volunteers promoted higher levels of functional activity in the SMFA. Indeed, there was a correlation between the SMFA activity and 6C-specific IgG levels. Recently the humanized mAb TB31F, which targets the conformational epitope I in the Pfs48/45-6C domain, was tested in a phase I study (28). This study showed that approximately 2.1 μg/mL of TB31F was required to promote 80% TRA. The geometric mean of anti-6C IgG concentration was 2.5 and 7.1 in the R0.6C-AlOH/MM and ProC6C-AlOH/MM groups, respectively, suggesting that the higher quantity of anti-6C IgG in ProC6C-vaccinated individuals could explain the higher frequency of samples with greater than 80 TRA from this group. However, as the R0.6C and ProC6C vaccines most likely elicit polyclonal responses not restricted to Pfs48/45-6C epitope I, it is difficult to extrapolate direct comparisons. Future experiments such as competition ELISA with TB31F are merited to investigate the proportion of 6C responses that target epitope I elicited by R0.6C and ProC6C vaccines. The Pfs230-Pro IgG concentration also correlated with the SMFA activity, but the correlation was less strong, suggesting that Pfs48/45-specific IgG is the main contributor to the functional activity observed here. The level of SMFA activity suggested that ProC6C-AlOH/MM elicits high levels of functional antibodies as compared with Pfs25-based vaccines and comparable levels to Pfs230-based vaccines (26).

At 6 months after immunization, the Pfs48/45 antibody titers were still higher than the controls in the 30 and 100 μg ProC6C-AlOH/MM (G1D and G2D), 100 μg ProC6C-AlOH/MM (G2B), and 100 μg R0.6C-AlOH/MM (G2B) groups. Since effective interruption of the spread of the parasite in the population most likely requires high levels of long-lasting TB antibodies, it was of interest to investigate whether natural malaria exposure modulates vaccine-specific antibody responses. We found that R0.6C and ProC6C vaccine-specific responses were boosted by preexisting immunity against their respective constituent antigens, GLURP, Pfs230, and CSP. This finding contrasts with prior findings that exposure to *P*. *falciparum* might diminish subsequent boosting by vaccination (29). Somewhat surprisingly, there was no boosting effect of concomitant *P*. *falciparum* infections on vaccine-specific responses, even though all constituent antigens elicit strong antibody responses in naturally exposed populations (reviewed in refs. 7, 30).

While the primary objective of this study was to investigate the TB properties of two TBVs, the CSP sequence in the ProC6C vaccine candidate also elicited anti-CSP IgG antibodies. The epitope specificity and functional activity of these CSP antibodies will be further investigated and compared with levels elicited by other CSP-based vaccines. Combining CSP and the sexual-stage antigens Pfs230 and Pfs48/45 may thus be attractive since it could reduce the risk of infections and onward transmission to other individuals via mosquitoes simultaneously.

Taken together, this first-in-human phase I clinical study supports the selection and further development of ProC6C as a promising transmission-blocking vaccine candidate and warrants further interrogation of the study samples to determine the CSP-based immune response elicited by this chimeric antigen.

## Methods

### Sex as a biological variable.

This clinical trial protocol included both males and females for enrollment. Sex was not considered a biological variable in the results reported here.

### Study population.

The study was conducted in the Sabou health district (SHD) area located about 100 km west of Ouagadougou. The SHD covers an area of 449 km^2^ in the region of Boulkiemdé and has 112,485 inhabitants, most of whom live in small rural villages in houses made of mud or cement walls and thatched or metal roofs. The population is stable and is mainly composed of the Mossi ethnic group; farming is the main activity. Demographic data (ethnicity only) were collected but are not reported here, as ethnicity is not considered a biological variable in the results presented. The SHD includes 20 peripheral heath facilities, which represent the primary point of contact with the health system. The climate consists of a single rainy season from May to October followed by a long dry season. The entomological inoculation rate was estimated at 31.4 infective bites per person per year in a study conducted in the neighboring Saponé Department, and the incidence rate was 2.2 (95% CI, 1.9 to 2.5) episodes per child-year at risk (31). This defines the highly seasonal malaria transmission with most malaria episodes experienced during or immediately following the rainy season.

### Study participants.

Study participants were healthy adults, aged 20–45 years, residing in the study area and available to follow-up. Participants were eligible if they had provided written informed consent. They had no evidence of acute or chronic illness or hematological, hepatic, or renal pathology, no history of malignancy of any organ system (other than localized basal cell carcinoma of the skin), treated or untreated, within the past 5 years, and no history of autoimmune disease. Other specific exclusion criteria included prior receipt of an investigational malaria vaccine, recent or planned used of an investigational drug, vaccine, immunoglobulin, or any blood product, confirmed or suspected immunodeficiency history, surgical splenectomy, history of anaphylaxis, known severe hypersensitivity to any of the vaccine components (adjuvant, antigen, or excipient), and participation in any other clinical study involving an investigational product in the 30 days prior to the start of the study or during the study period. The full list of inclusion and exclusion criteria is given in the Study Protocol. The full Study Protocol is available in the supplemental material. All disease episodes were treated according to standard of care in Burkina Faso. Malaria cases were managed according to the National Malaria Control Program guidelines.

### Study design and procedures.

The study, TBVax1 (Figure 1), was a randomized, staggered, adjuvant-selection, dose-escalation phase I clinical trial. Randomization was done in randomly permuted blocks using R Statistical Software with randomizr and blockrand packages. Within each dose level cohort (30 μg or 100 μg protein), the participants (*n* = 5 or *n* = 20, respectively) were randomized to receive either study vaccine (R0.6C-AlOH or ProC6C-AlOH; Statens Serum Institut) alone or supplemented with Matrix-M adjuvant (Novavax) or a placebo Hep B vaccine, Euvax B (LG Chem, South Korea). The R0.6C-AlOH and ProC6C-AlOH vaccines were administered in a fractional dose fashion to achieve 30 μg or 100 μg protein in either a 0.15 mL or a 0.5 mL volume. The vaccines, when combined with Matrix-M, resulted in either a 15 μg or a 50 μg dose of Matrix-M respective to the 30 or 100 μg protein dose and resulted in a total volume of administration of 0.19 mL or 0.63 mL. Upon enrollment and before vaccination, no medication or treatment for malaria was administered. At the conclusion of the study, all study participants were given antimalarial medication. All vaccine doses were given as intramuscular injections into the deltoid muscle, alternately in the left and right arms. A series of 3 immunizations was given on D0, D28, and D56. For each cohort, allocation to a treatment number was based on the order in which the individual presented for vaccination. An independent pharmacist prepared the syringes with masking tape, to maintain blinding. The vaccinator was not involved in other activities in the trial. Following the primary vaccination of cohort 1 (30 μg study vaccine) and DSMB review, cohort 2 (100 μg study vaccine) was initiated. All volunteers were monitored by daily home visits for 7 days after each vaccination by a team of qualified nurses under the supervision of the study physician. If necessary, a volunteer was referred to the study clinic for assessment by the study physician. All study participants were encouraged to attend the study clinic if they felt unwell. The study medical staff were available 24 hours 7 days a week. Unsolicited AEs were recorded until 1 month after each vaccination. Serious AEs and malaria episodes were monitored throughout the study duration. Clinical or symptomatic malaria for this study is defined as the presence of asexual *P*. *falciparum* parasites at any parasitemia with temperature of ≥37.5°C and/or one or more of the following symptoms: headache, myalgia, arthralgia, malaise, nausea, dizziness, or abdominal pain. The primary endpoint of the trial was the number and grade of AEs and serious AEs possibly, likely, or definitely related to vaccination. Severity of AEs was graded as mild (grade 1), moderate (grade 2), severe (grade 3), or potentially life-threatening (grade 4). The secondary endpoints were vaccine-specific IgG concentrations and functional activity evaluated in the SMFA. Results reported follow the 2010 Consolidated Standards of Reporting Trials (CONSORT) guidelines as applicable (32).

### Study vaccines.

The R0.6C vaccine candidate consists of the GLURP-R0 domain genetically fused to the Pfs48/45-6C domain (14), and the ProC6C vaccine candidate consists of the Pfs230-Pro domain genetically fused to the 6C domain through a spacer sequence derived from *P*. *falciparum* CSP (13). Vaccine drug substances were manufactured in the *Lactococcus lactis* expression system according to current good manufacturing practice guidelines at Statens Serum Institut using a conventional batch process as previously described in detail (13, 14) at the 30 L scale. Drug substances were formulated to drug product by dilution to 200 μg/mL protein in 10 mM HEPES, 2.5% glucose, 0.5 mM EDTA, 155 mM NaCl and absorbed to 1.6 mg/mL Alhydrogel (Brenntag, Denmark) at a fill volume of 0.8 mL in a 2 mL borosilicate glass vial. The drug products were aseptically vialed at Baccinex (Switzerland) and stored at 2°C to 8°C. Vaccine drug products were maintained according to an International Conference on Harmonization stability protocol for the duration of the study.

The Matrix-M adjuvant (21) was supplied by Novavax. A single lot of the adjuvant was used for the duration of the study. Matrix-M was supplied at a concentration of 0.375 mg/mL and stored at 2°C to 8°C. For groups receiving Matrix-M, 0.210 mL of Matrix-M was withdrawn and mixed with the vaccine drug product vial (either R0.C6-AlOH or ProC6C-AlOH) and administered within 6 hours after admixture.

### Clinical laboratory evaluations.

Two milliliters of blood was collected by venipuncture into EDTA tubes for hematology and 3 mL of blood into serum separator tubes (SSTs) for biochemistry. Full blood counts and biochemistry were done using calibrated automatic analyzers. *P*. *falciparum* parasitemia was assessed using 2 independent reads of Giemsa-stained thick blood smears at ×100 magnification followed by a third read in case of discordance (disagreement on positivity or a >2-fold difference in parasitemia). The limit of detection for asexual parasitemia was 100 trophozoites/μL. Parasite density was calculated as number of asexual parasites/μL of blood assuming a mean normal leukocyte count of 8,000/μL. The inherent threshold of detection by light microscopy was 8 gametocytes/μL of blood.

### Immunogenicity laboratory evaluations.

Ten milliliters of blood was collected by venipuncture into SSTs for immunogenicity. Antigen-specific IgG antibody levels were determined by ELISA as previously described (8). In brief, 96-well plates (Nunc MaxiSorp) were coated with 0.5 μg/well of Pfs48/45-6C (33), R0.6C (12), ProC6C (13), Pf230-Pro, PfCSP4/38 (34), or GLURP-R0 (35) as appropriate. Serum from volunteers was analyzed at indicated concentrations (1:100 through 1:24,300). Antibody concentrations were calculated through regression analysis using TB31F (28) monoclonal antibody (serially diluted 0.2 μg/mL through 0.002 μg/mL) as a reference for antigens, which included Pfs48/45-6C, mAb311 (36) for PfCSP4/38, and a pool of Liberian plasma for Pfs230-Pro and GLURP-R0. Antigen-specific antibodies were detected using horseradish peroxidase–conjugated polyclonal rabbit anti-human IgG (Agilent, Denmark), diluted 1:3,000, followed by 3,3′, 5,5′-tetramethylbenzidine dihydrochloride (TMB) substrate and stopped by addition of 100 μL H_2_SO_4_. Data were processed using Quant Assay for Windows (version 0.7.1.4) and GraphPad Prism version 8.3.0.

### Biological activity laboratory evaluations.

A second 10 mL of blood was collected by venipuncture into SSTs for functional antibody determination with SMFA. The biological activity of samples from volunteers was evaluated at 15 mg/mL purified IgG. The assay has been qualified (19), and was conducted at the National Institute of Allergy and Infectious Diseases (NIAID). The standardized methodology for performing the SMFA has been described previously (19). In brief, 16- to 18-day-old gametocyte cultures of the *P*. *falciparum* NF54 line were mixed with purified IgGs from individual study volunteers at indicated concentrations and fed to *Anopheles stephensi* mosquitoes. All feeding experiments were performed with human complement, and 20 mosquitoes per group were examined 8 days after the feeding experiment for oocyst counts.

### ProC6C-specific antibody depletion.

From the G2D (100 μg ProC6C-AlOH/MM) group, 2 pools of D70 total IgG were prepared based on the individual TRA level. The high (hi) pool contained IgGs that showed >95 TRA (*n* = 6), and the low (lo) pool contained <80 TRA (*n* = 5). Similarly, 2 more pooled D70 IgGs were prepared from the Hep B (G2E) group; the hi pool contained >80%TRA (*n* = 2), and the lo pool <50 TRA (*n* = 5). Anti-ProC6C antibodies were depleted from the pooled IgGs using a ProC6C recombinant protein immobilized column as described elsewhere (37). Anti-ProC6C antibody level in the original and depleted IgGs was determined by ELISA.

### Statistics.

For the safety analysis, data from all individuals who received at least 1 dose and for whom safety data were available were included. All analyses were descriptive with data presented by dose, overall/dose, and overall/individual. Results were summarized by the study group. The percentages of individuals with at least 1 local AE (solicited or unsolicited), with at least 1 general AE (solicited or unsolicited), and with any AE during the solicited follow-up period were tabulated. The same calculations were performed for AEs rated as grade 3. The percentage of individuals reporting each individual solicited local and general AE during the solicited follow-up period was tabulated. The same tabulation was performed for grade 3 AEs and for AEs with relationship to vaccine administration.

The limit of detection (LOD) for R06C, ProC6C, and 6C ELISA was 0.033, 0.169, and 0.200, respectively. Any values less than the LOD were assigned as half of the LOD values (0.016, 0.08, and 0.100, respectively) for the analysis. For the comparisons of ELISA data among different groups at the same time point, 1-way ANOVA followed by Tukey’s multiple-comparison test was performed using log-transformed ELISA titers. One-tailed *t* test was also performed. The fold increase from D0 to D70 (D70 titer/D0 titer) and fold decrease from D70 to D180 (D70 titer/D180 titer) were calculated individually, and the difference among groups was evaluated by 1-way ANOVA followed by Tukey’s multiple-comparison test. For SMFA results (percentage inhibition in oocyst density, TRA), statistical analysis was completed via Kruskal-Wallis test followed by Dunn’s multiple-comparison test. The correlation between 2 continuous values was assessed by Spearman’s rank test, and a χ^2^ test was used to determine the impact of parasite-positive visits on fold increase in titers from D0 to D70. All statistical tests were performed using GraphPad Prism version 8.3.0, and *P* values less than 0.05 were considered significant.

### Study approval.

The clinical trial protocol and associated documents were reviewed and approved by the Burkina Faso Ministry of Health Ethical Committee for Biomedical Research (approval reference 2021-02-034). Regulatory approval (approval reference 2022-02041) was given in Burkina Faso by the National Regulatory Authority (Comité Technique pour les Essais Cliniques). All study participants gave documented informed consent before any study procedures were performed. The trial was conducted according to the principles of the Declaration of Helsinki and International Conference on Harmonization Good Clinical Practice (GCP) guidelines. An independent DSMB and local safety monitors provided safety oversight, and GCP compliance was independently monitored by an external organization (ClinaPharm, Cotonou, Benin). The clinical trial was registered in the Pan-African Clinical Trials Registry (https://pactr.samrc.ac.za) under ID no. PACTR202201848463189.

### Data availability.

Data used in the compilation of figures presented in this article are supplied in the Supporting Data Values file. Data are available upon reasonable request.

## Author contributions

ABT, JLP, and M Theisen drafted the manuscript with input from KM, MN, JMR, and IS. All authors read and approved the manuscript. ABT and SBS were responsible for the overall conduct of the clinical trial, protocol development, and approvals. JLP assisted in design, manufacture, and preclinical and clinical development of R0.6C and ProC6C antigens, including formulation development and pharmacy handling. M Theisen and JLP assisted in clinical trial protocol development and training of pharmacy staff. MN, BA, JLP, M Theisen, and KM performed serological and statistical analysis and preparation of figures. MN collated and maintained study databases for serological analysis. SE and EAO assisted in serological analysis and specimen management. KM and CAL performed functional analysis of trial specimens, statistical analysis, and preparation of figures. CAL reviewed and provided input on the manuscript. SKS assisted in design, manufacture, and preclinical development of R0.6C and ProC6C, including preparation of proteins used. KLB, CC, and JMR provided critical advice on formulation and adjuvant development and clinical trial protocol development and supplied Matrix-M adjuvant and supporting data. Alphonse Ouedraogo, BIS, ECB, AB, JBBY, NH, AK, AD, Amidou Ouedraogo, and INO participated in the conduct of the clinical trial and clinical trial responsibilities. M Tienta served as data manager for TBVax1. IS served as study sponsor, including protocol development, DSMB management, and oversight of study databases. M Theisen conceived the development of R0.6C and ProC6C as principal investigator for the preclinical development and oversaw the manufacture of drug substances and products.

## Supplementary Material

Supplemental data

ICMJE disclosure forms

Supporting data values

## Figures and Tables

**Table 6:**
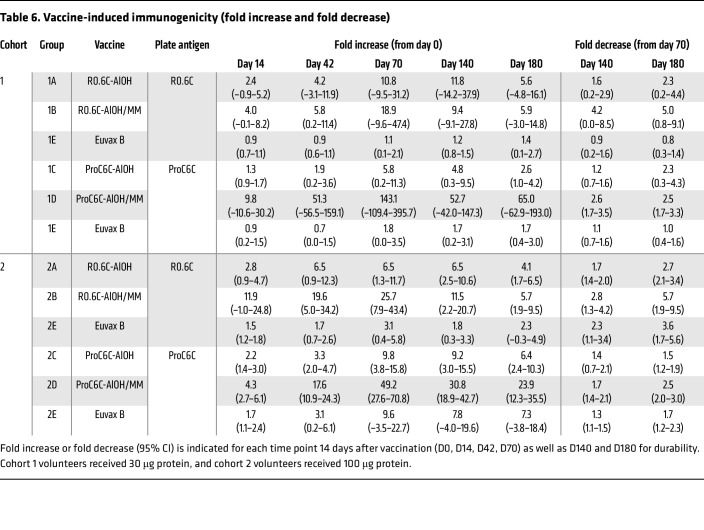
Vaccine-induced immunogenicity (fold increase and fold decrease)

**Table 5:**
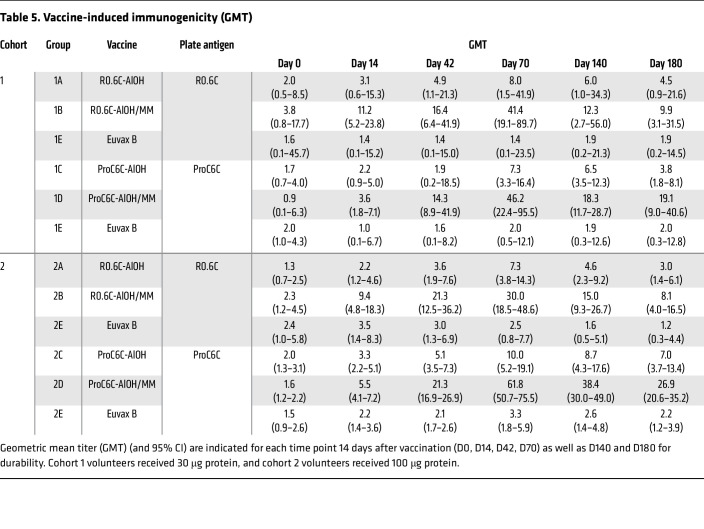
Vaccine-induced immunogenicity (GMT)

**Table 4:**
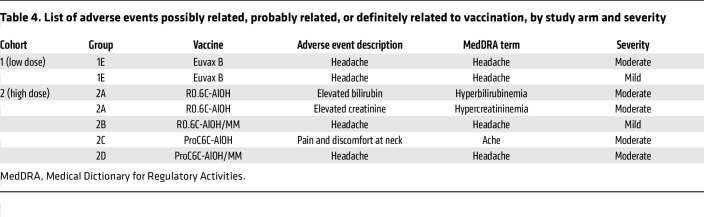
List of adverse events possibly related, probably related, or definitely related to vaccination, by study arm and severity

**Table 3:**
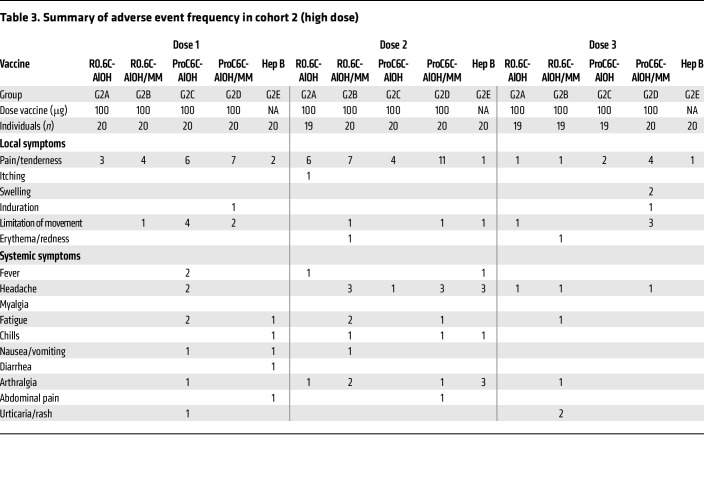
Summary of adverse event frequency in cohort 2 (high dose)

**Table 2:**
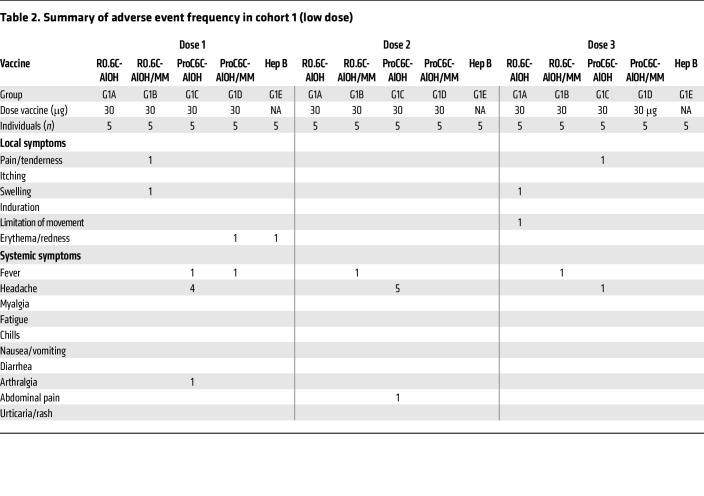
Summary of adverse event frequency in cohort 1 (low dose)

**Table 1:**
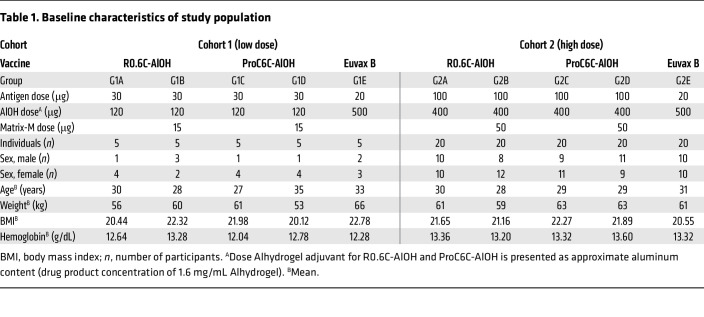
Baseline characteristics of study population

## Version 1 01/30/2024

In-Press Preview

## Version 2 04/01/2024

Electronic publication
