# Supplementary material for: A randomized first-in-human phase I trial of differentially adjuvanted Pfs48/45 malaria vaccines in Burkinabé adults
Source: J Clin Invest. 2024 Apr 1;134(7):e175707. doi: 10.1172/JCI175707 (PMC10977980; doi:10.1172/JCI175707)
Supplement: Supplemental data [file jci-134-175707-s122.pdf]

Supplemental Information

A randomized first-in-human Phase 1 trial of differentially adjuvanted Pfs48/45 malaria vaccines in Burkinabé adults

This supplemental contains the following items:

1. Supplemental Tables
2. Supplemental Figures
3. Study Protocol
4. Pharmacy Manual

Supplemental Table S1: Unsolicited adverse events by study cohorts and vaccination groups

Cohort 1 (Low Dose)

	Group/Study Vaccine					
	1A	1B	1C	1D	1E	
**Description**	R0.6C-AlOH	R0.6C-AlOH/MM	ProC6C-AlOH	ProC6C-AlOH/MM	Hepatitis B	**Total Events**
Uncomplicated Malaria	3	2	1	1	2	9
Headache	1	-	2	1	2	6
Bronchitis	1	1	1	-	-	3
Rhinitis	-	-	-	2	-	2
Gastroduodenal ulcer	-	1	1	-	-	2
Low Respiratory Tract Infection	1	-	-	-	-	1
Pityriasis versicolor	-	-	1	-	-	1
Dengue	1	-	-	-	-	1
Diffuse pain	-	-	1	1	-	2
Dysphagia	-	-	1	-	-	1
Epigastralgia	-	-	1	-	-	1
Fatigue	-	-	-	1	-	1
Generalized pruritis	-	-	-	1	-	1
Toothpain	-	-	1	-	-	1
Vaginal mycosis	-	-	1	-	-	1
**Total**	**7**	**4**	**11**	**7**	**4**	**33**

Cohort 2 (High Dose)

	Group/Study Vaccine					
	2A	2B	2C	2D	2E	
**Description**	R0.6C-AlOH	R0.6C-AlOH/MM	ProC6C-AlOH	ProC6C-AlOH/MM	Hepatitis B	**Total Events**
Uncomplicated Malaria	11	5	2	7	7	32
Bronchitis	1	5	2	4	1	13
Rhinitis	3	4	1	1	-	9
Headache	1	2	1	2	-	6
Gastroduodenal ulcer	-	-	1	-	-	1
Abdominal hernia	1	-	-	-	-	1
Abdominal pain	1	-	-	-	-	1
Arthralgia	-	-	-	1	-	1
Chills	-	-	-	-	1	1
Cough	-	-	-	1	1	2
Diffuse pain	-	-	1	-	-	1
Elevated bilirubin	1	1	-	-	-	2
Elevated creatinine	1	-	-	-	-	1
Epigastralgia	-	-	-	1	1	2
Furunculosis	-	-	1	-	-	1
Gastroenteritis	-	-	-	1	1	2
Lumbago	-	-	-	1	-	1
Neck pain	-	-	1	-	-	1
Panarias	1	-	-	-	1	2
Parasitosis	-	-	1	-	-	1
Toothpain	-	1	-	-	-	1
Torticollis	-	2	-	-	-	2
Trauma left cheek	-	1	-	-	-	1
**Total**	**21**	**21**	**11**	**19**	**13**	**85**

Supplemental Figure S1. Total number of unsolicited adverse events (AEs) by study cohort and vaccine (A) and incidence of AEs by vaccine group (B). The incidence was calculated as the cumulative number of AE recorded in the vaccine group divided by the total number of doses administered in this group.

A

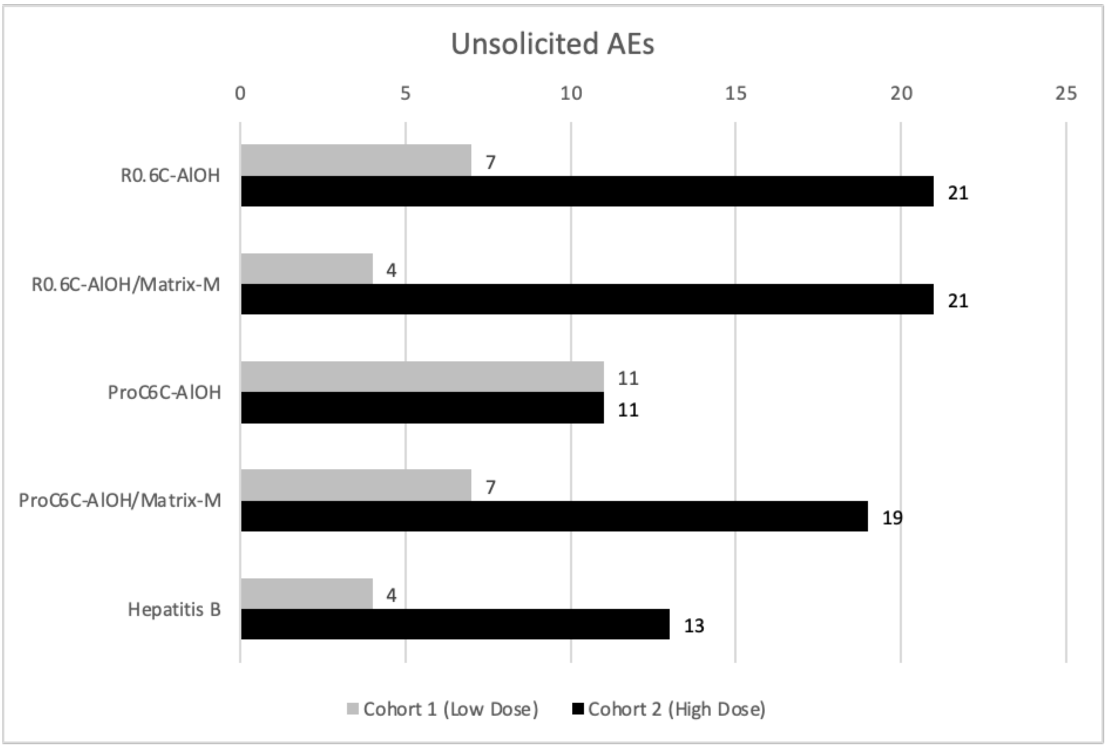

B

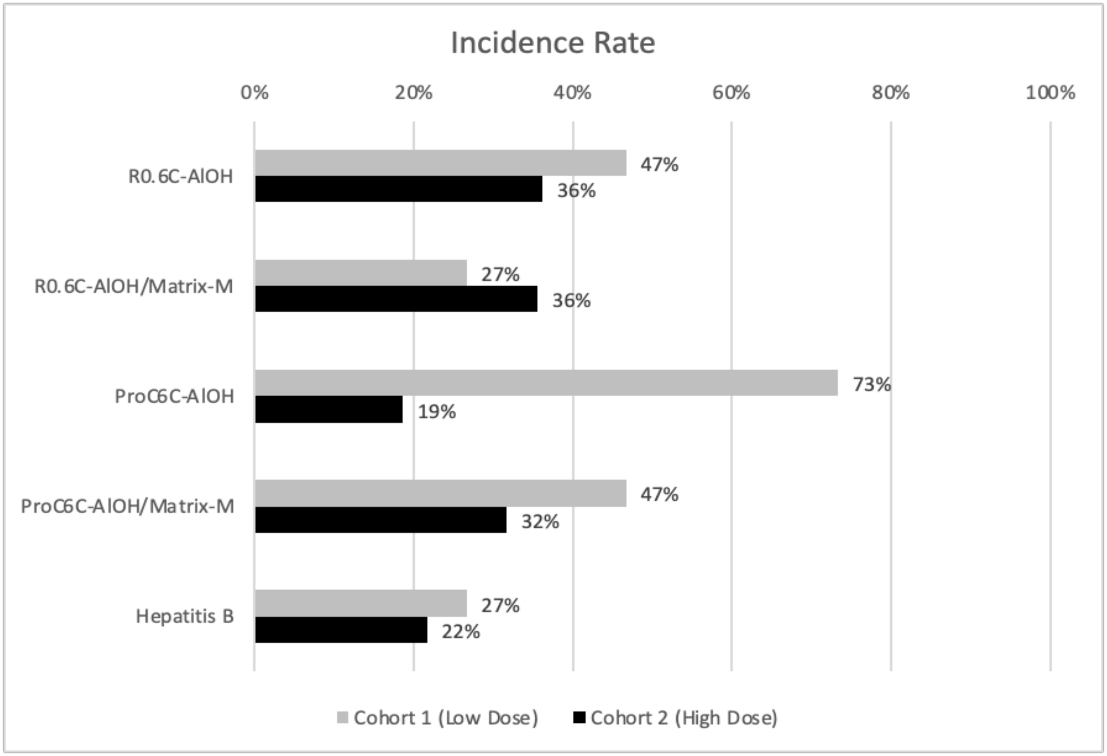

Supplemental Figure S2. Vaccine induced immunogenicity (Cohort 1). The immunogenicity to the vaccine immunogens (R0.6C or ProC6C) is evaluated at each study timepoint (D0, 14, 42, 70, 140, and 180). Groups containing the -AlOH adjuvant alone (geometric mean, red line) and -AlOH/MM adjuvant (geometric mean, blue line) are plotted independently. Cohort 1 volunteers received 30 µg protein. The control group received Euvax B vaccine (Hep B, G1E) and plotted to each vaccine immunogen (geometric mean, black line). Individual volunteers are plotted by gray lines for each group. GMT and fold increase/decease are indicated in Table 5 and 6 respectively.

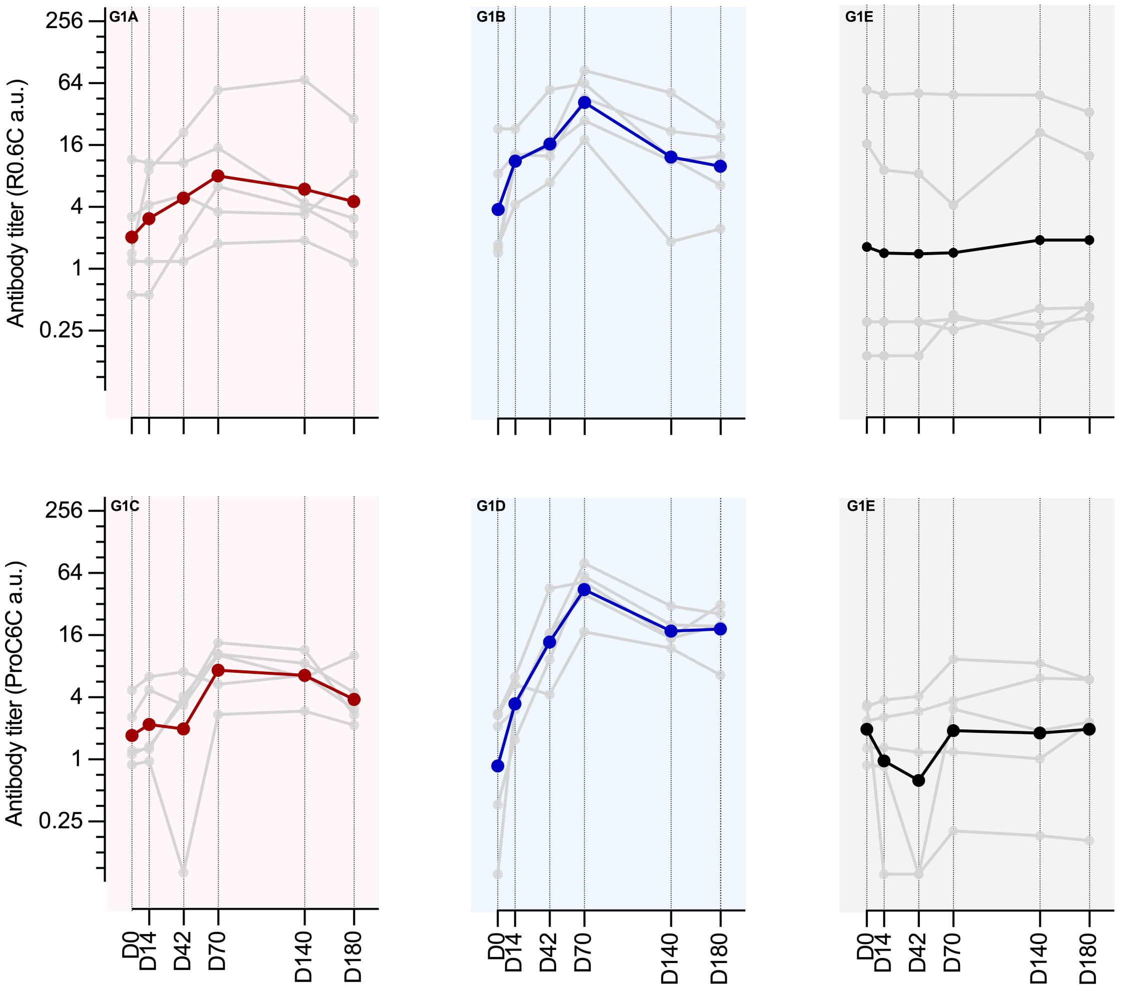

Supplementary Figure S3. Effect of parasite positivity on antibody titer. Antibody levels to vaccine immunogen in individuals with (+) or without (-) parasites determined at time of blood collection (D0 or D70). Parasite presence (-) means no asexual and gametocyte parasites. Parasite presence (+) means either asexual (+), gametocyte (+) or both (+). Geometric mean indicated by horizontal line. There were no significant differences at either timepoint as determined by unpaired t test. The mean and range of asexual and gametocyte parasites present (+) are noted for each group. 2A: Asexual D0 (2813; 484-7722) and D70 (343; 115-718). Gametocytes D0 (0;0) and D70 (16; 4-28). 2B: Asexual D0 (355; 177-577) and D70 (514; 118-1254). No Gametocytes present. 2C: Asexual D0 (383; 147-775) and D70 (171; 140-230). No gametocytes present. 2D: Asexual D0 (290; 141-325) and D70 (1083; 1083-1083). Gametocytes D0 (4;4-4) and D70 (54; 8-124).

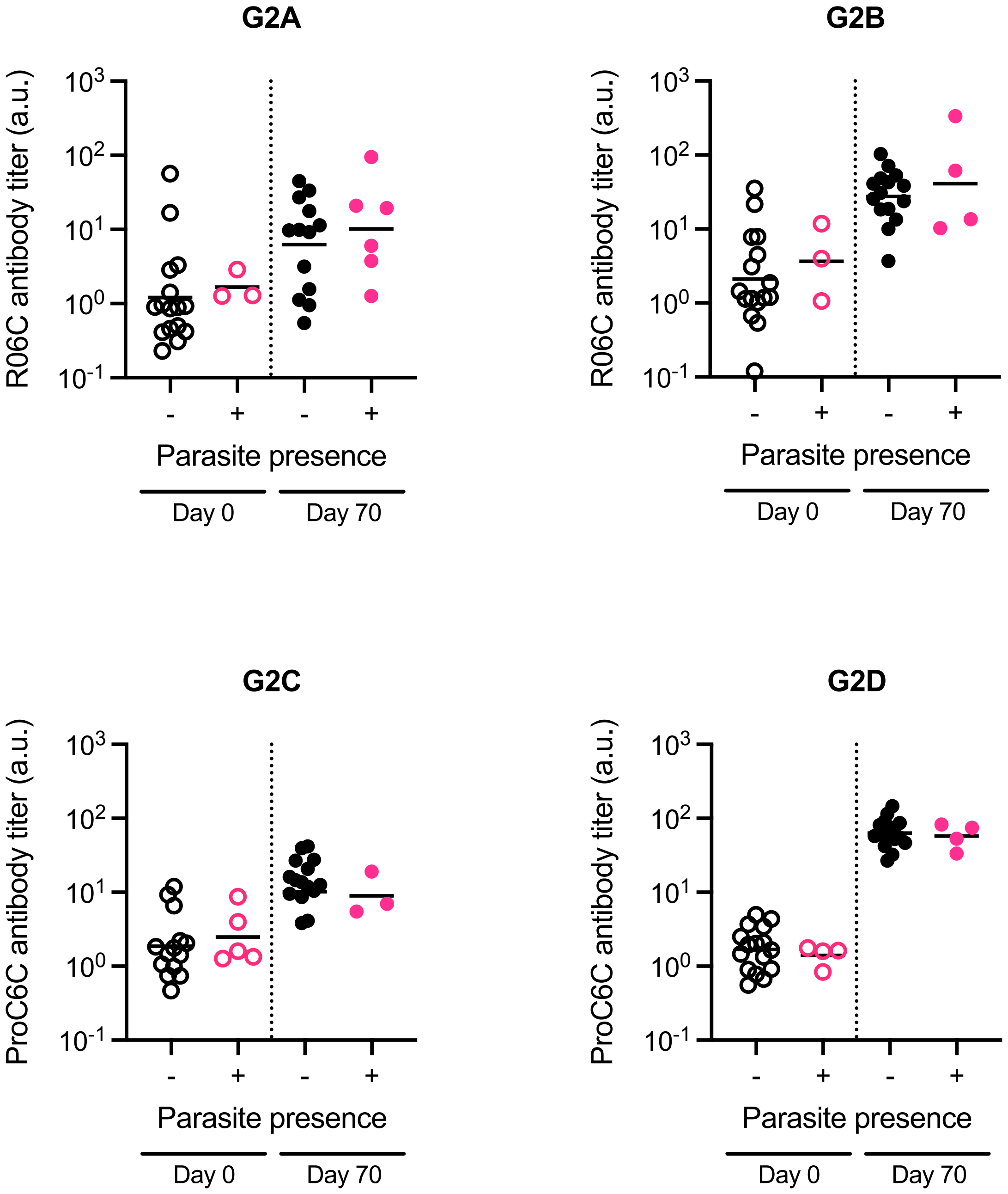

Supplementary Figure S4. Effect concurrent infection on vaccine antibody responses. (A) Number of individuals positive for parasites at study visits up through D70 (n=6 visits). No parasites at any study visit (0, gray); one positive study visit (1, pink), two positive study visits (2, green), three positive study visits (3, purple); and four positive study visits (4, yellow). There was a median of 1.27 positive visits per individual across the the four vaccine groups (G2A, G2B, G2C and G2D). **(B)** Fold increase (D70/D0) of R0.6C or ProC6C antibody (ab) as compared to number (#) of concurrent parasite positive visits from D0 to D70. Open circles indicate individuals with horizontal line median.

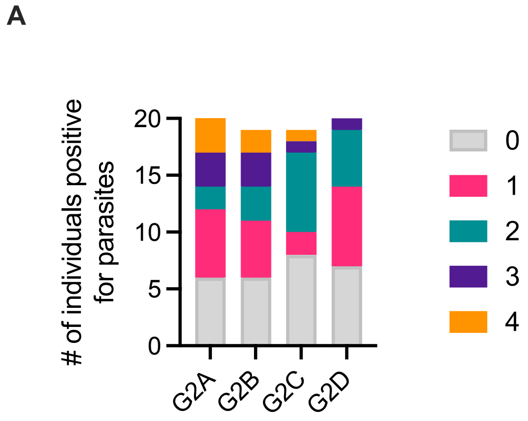

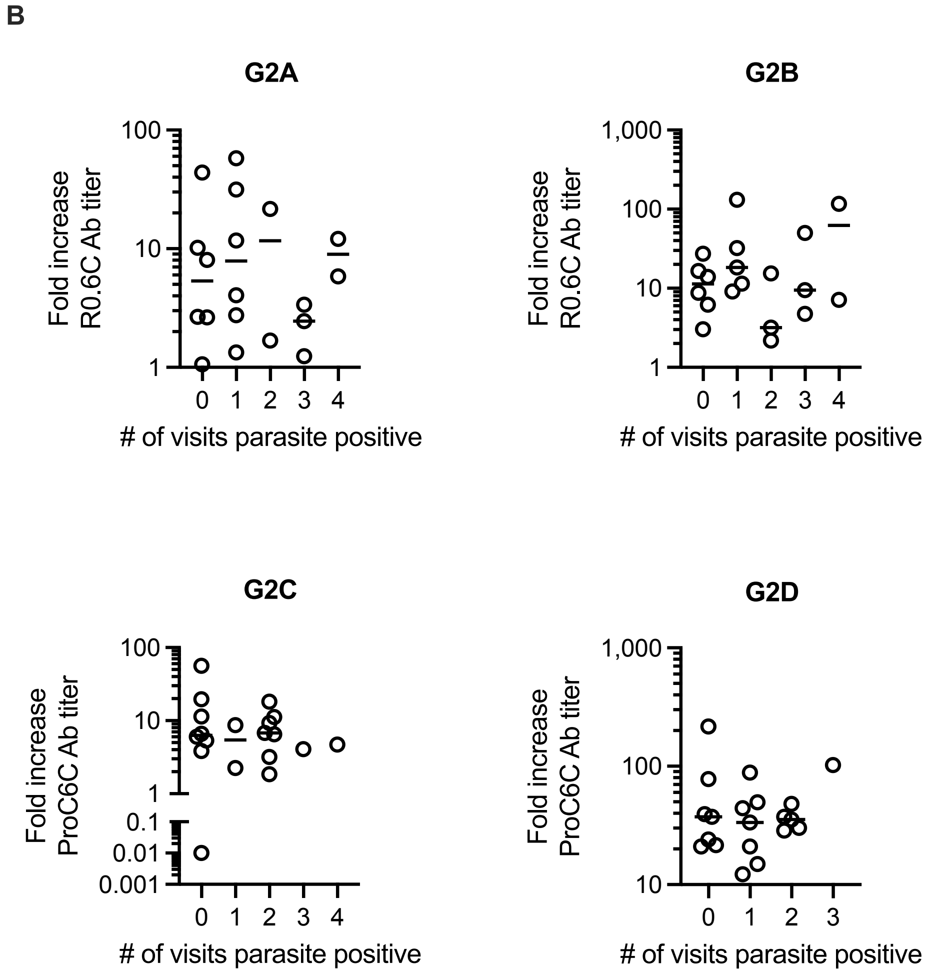

Supplementary Figure S5. Antibody correlation between antibody levels at baseline(D0) and D70 . Correlation of specific antibody levels in volunteers receiving **(A)** R0.6C vaccines (G2A, G2B) and **(B)** ProC6C vaccines (G2C, G2D). Spearman rank *P* values are shown for each group (Red or Blue) symbol and *P* value.

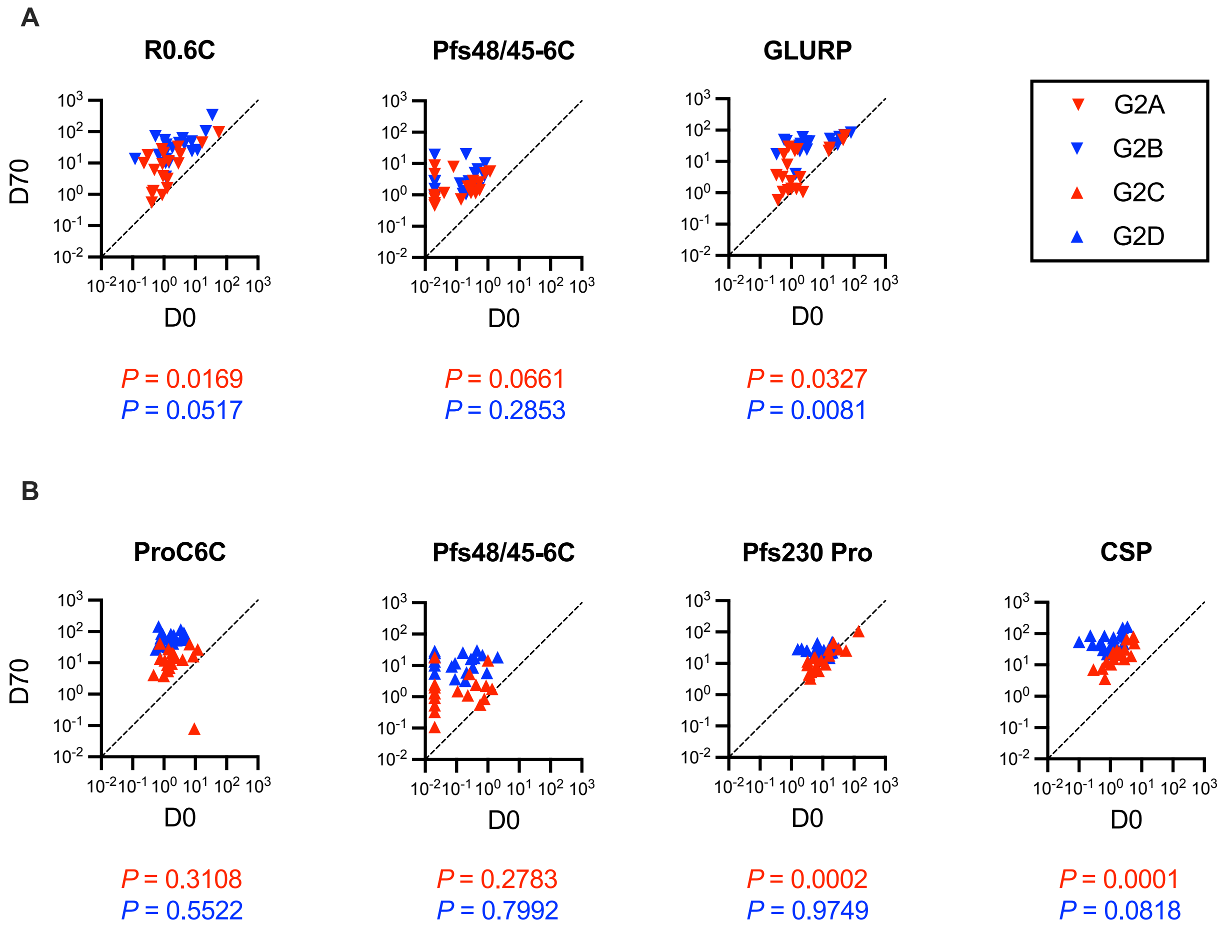

Supplemental Figure S6. Biological and antibody correlation (Pfs230-Pro). Purified IgG used for analysis in the SMFA was also evaluated in ELISA to Pfs230-Pro antigens. The biological activity is plotted in log of mean oocyst ratio (LMR) between control and test IgGs (y-axis) respective to square root (sqrt) of D70 antibody levels of Pfs230-Pro (x-axis). For ease of comprehension, y-axis shows corresponding %TRA values, instead of LMR. The R0.6C groups (all individuals) are plotted in the left panel and ProC6C groups (all individuals) plotted in the right panel using the same symbols used in Figure 5. The Spearman rank p value and correlation coefficient (r) for all individuals in each panel are shown. Groups receiving Matrix-M adjuvant (blue symbols) or -AlOH adjuvant alone (red symbols). Cohort 1 (low dose, 30 µg protein) are indicated by open symbols and Cohort 2 (high dose, 100 µg protein) by closed symbols.

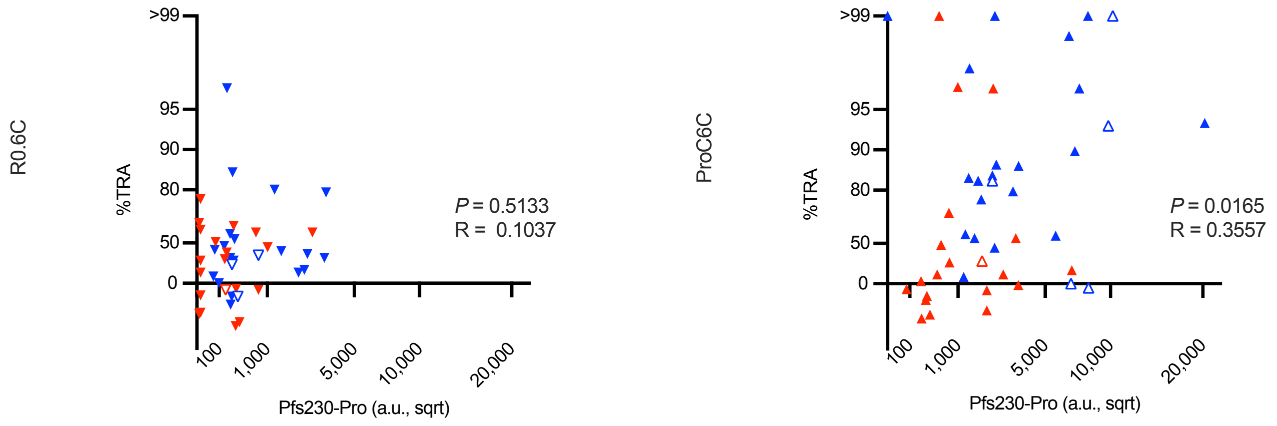

Phase 1 Dose Escalating, Double-Blind, Randomised Comparator Controlled Trial of the Safety and Immunogenicity of Different Adjuvant Formulations of R0.6C and ProC6C transmission blocking vaccines candidates against *Plasmodium falciparum* in Adults in Burkina Faso (TBVax1).

Conducted by:
Groupe de Recherche Action en Santé (GRAS), Burkina Faso
and
Serum Statens Institute (SSI), Denmark

Investigational New Drug (IND) Sponsor: University of Sciences, Technics and Technologies of Bamako (USTTB), Mali through PfTBV EDCTP RIA2018SV-2311: 2019-2024

IND Number: To be provided

Version: Amendment 3.0
March 16th, 2022

CONFIDENTIAL

Team Roster

Study Staff Roster

**Center :**	Groupe de Recherche Action en Santé (GRAS)
**Principal Investigator:**	Dr. Alfred B. TIONO, MD, PhD<br>Groupe de Recherche Action en Santé (GRAS<br>Tel: (+226) 25 35 56 90<br>Email : a.tiono@gras.bf
**Clinical Associate Investigators**	Dr. Sodiomon B. SIRIMA<br>Dr. Alphonse OUEDRAOGO<br>Dr. Jean SAWADOGO<br>Dr Ben Idriss SOULAMA<br>Dr Maurice San OUATTARA
**Laboratory Associaties Investigators:**	Dr. Issa N. OUEDRAOGO<br>Dr. Amidou DIARRA<br>Dr. Edith BOUGOUMA
**Data Management:**	Mr. Amidou OUEDRAOGO<br>Mr. Benjamin SOMBIE
**Participating sites**	Groupe de Recherche Action en Santé (GRAS<br>District Sanitaire de Sabou<br>Centre Medical Saint Maximilien Kolbe (CMAMK)
**Laboratories:**	LMIV/NIAID/NIH<br>5640 Fishers Lane, Twinbrook I Rockville, Maryland 20852<br><br>Serum Statens Institute (SSI),<br>Copenhagen, Denmark<br><br>Groupe de Recherche Action en Santé (GRAS)<br>Ouagadougou

Centre Medical Saint Maximilien Kolbe (CMAMK)

Institutional Review Board: Comité d'Ethique de Recherche en Santé, (CERS) Burkina Faso

Center: Serum Statens Institute (SSI),
Copenhagen, Denmark

Principal Investigator: Prof. Michael Theisen

Associate Investigators Dr. Jordan Plieskatt

Safety Oversight

Data and Safety Monitoring Board (DSMB):
PfTBV EDCTP USTTB

Independent Safety Monitor:
To be named

Clinical Trial Site
District Sanitaire de Sabou
Centre Medical Saint Maximilien Kolbe (CMAMK), West Africa and surrounding villages
Burkina Faso, West Africa

Laboratories
Clinical Laboratories:
CMAK/GRAS Clinical Laboratory
GRAS
BP 06 Ouagadougou 01
Burkina Faso

Collaborating Laboratories:
LMIV/NIAID/NIH
BG 29B
9000 Rockville Pike
Bethesda, MD 20814, USA

PATH's Center for Vaccine Innovation and Access
Direct: +1 202.540.4383
Web: www.path.org | www.malariavaccine.org
455 Massachusetts Avenue NW, Suite 1000
Washington, DC 20001 USA
Main: +1 202.822.0033 Fax: +1 202.457.1466

MRTC/DEAP Clinical Laboratory
FMPOS/USTTB
BP 1805, Point G
Bamako, Mali

PROTOCOL SIGNATURE PAGE

The signatures below constitute the approval of this protocol and the attachments, and provide the necessary assurances that this trial will be conducted according to all stipulations of the protocol, including all statements regarding confidentiality, and according to local legal and regulatory requirements and applicable US federal regulations and ICH guidelines.

Site Principal Investigator:

Signed: ______________________________ Date: ______________________________

Alfred B. Tiono MD PhD
Senior associate researcher, GRAS

TABLE OF CONTENTS

Study Staff Roster ... 2
List of tables ... 8
List of figures ... 8
1. INTRODUCTION AND RATIONALE ... 14
1.1 Introduction ... 14
1.2 Rationale for a transmission blocking vaccine ... 15
2. Preclinical studies with R0.6C ... 15
2.1.1 Summary of preclinical studies with R0.6C ... 15
2.1.2 Immunogenicity of R0.6C/AlOH and R0.6C/AlOH + Matrix-M adjuvant ... 16
3. Preclinical studies with ProC6C ... 21
3.1 Summary of preclinical studies ... 21
3.2 Immunogenicity of ProC6C/AlOH and ProC6C/AlOH + Matrix-M adjuvant ... 22
3.2.1 ProC6C/AlOH in mice ... 22
4. MATRIX M ... 23
4.1 Summary of Clinical Experience with Matrix-M ... 24
5. OBJECTIVES ... 27
5.1 Primary Objective: ... 27
5.2 Secondary objectives; ... 27
5.3 Exploratory Objectives: ... 27
6. STUDY DESIGN ... 27
7. STUDY POPULATION ... 29
7.1 Site ... 29
7.2 Population ... 29
7.3 Inclusion criteria ... 30
7.4 Exclusion criteria ... 30
7.5 Sample size assumptions ... 31
7.6 Withdrawal of individual subjects ... 32
7.7 Replacement of individual subjects after withdrawal ... 33
7.8 Follow-up of participants withdrawn from treatment ... 33
7.9 Premature termination of the study ... 33
8. INVESTIGATIONAL PRODUCTS ... 33
8.1 The malaria vaccine candidates ... 33
8.1.1 Name and description of investigational product(s) ... 33
8.1.2 Storage of Investigational Medicinal Product ... 35
8.1.3 Preparation and labelling of Investigational Medicinal Products ... 35
8.1.4 Dosages, dosage modifications and method of administration ... 37
8.1.5 Shipment and Receipt ... 38
8.1.6 Accountability and Return of Product ... 38
8.2 The Hepatis B vaccine ... 39
8.3 Indications for deferral of vaccination ... 39
8.4 Absolute contraindication for subsequent vaccination ... 39

8.5 Concomitant medication/vaccination 40
9. METHODS 40
9.1 Community sensitisation 40
9.2 Randomisation, blinding and treatment allocation 41
9.3 Study procedures 41
9.3.1 General procedures 41
9.3.2 Specific procedures 44
9.4 Provision of care to the trial participants 51
9.4.1 Malaria case management 51
9.4.2 Pregnancy management 51
9.5 Blinding and code Breaking Procedure 51
10. SAFETY REPORTING 52
10.1 Adverse events (AEs) 52
10.1.1 Adverse event data collection and recording 52
10.1.2 Assessment of causality 55
10.1.3 Treatment of adverse events 56
10.1.4 Serious adverse events (SAEs) 56
10.1.5 Suspected unexpected serious adverse reactions (SUSARs) 57
10.2 Annual safety report 57
10.3 Follow-up of adverse events 58
10.4 Data Safety Monitoring Board (DSMB) 58
10.4.1 Local safety monitor (LSM) 58
10.4.2 Safety Meetings 58
10.4.3 Safety Reports 58
10.4.4 Dose progression 59
10.4.5 Safety holding rules 59
11. TRIAL ENDPOINTS 61
11.1 Primary endpoints: 61
11.2 Secondary endpoints: 61
11.3 Exploratory Endpoints: 61
12. DATA MANAGEMENT 61
12.1 Data Management 61
12.2 Data Capture Methods 61
13. STATISTICAL ANALYSIS 62
13.1 Primary study parameter 62
13.2 Secondary study parameter(s) 63
14. ETHICAL CONSIDERATIONS 63
14.1 Regulation statement 63
14.2 Recruitment and consent 63
14.3 Benefits and risks assessment 64
14.4 Compensation for injury 64
14.5 Incentives 64
15. ADMINISTRATIVE ASPECTS, MONITORING AND PUBLICATION 65

15.1 Handling and storage of data and documents 65
15.2 Monitoring and Quality Assurance 65
15.3 Amendments 66
15.4 Annual progress report 66
15.5 Temporary halt and (prematurely) end of study report 67
15.6 Public disclosure and publication policy 67
15.7 Timeframe and duration of the study 68
15.7.1 Timeline 68
15.7.2 Gantt chart 68
Appendix A. Toxicity Tables 69
16. REFERENCES 71

List of tables

Table 1: Summary of Preclinical Animal Studies for R0.6C candidate Vaccines 16
Table 2:: Design for rat Study of R0.6C/AlOH 17
Table 3: Summary of Transmission-Blocking activity of sera from rat immunized with R0.6C/AlOH 18
Table 4: Design for Mouse Study of R0.6C/AlOH 20
Table 5: Summary of Preclinical Animal Studies for ProC6C candidate Vaccines 21
Table 6: Design for Mouse Study of ProC6C/AlOH 22
Table 7. Key Clinical Experience of Matrix-M with Various Vaccine Antigens. 25
Table 7: . Subjects of each cohort 28
Table 8: Event detection probabilities for groups of size 20 32

List of figures

Figure 1: Schematic design of the study 12
Figure 2: Functional activity and immune recognition of R0.6C/AlOH in rats 19
Figure 3: Functional activity and immune recognition of R0.6C/AlOH with or without Matrix-M adjuvant in mice 20
Figure 4: Functional activity and immune recognition of ProC6C/AlOH with or without Matrix-M adjuvant in mice 23
Figure 5: Schematic design of the study 28
Figure 6: Schematic of R0.6C reconstitution and dosing 35
Figure 7: Schematic of ProC6C reconstitution and dosing 36

LIST OF ABBREVIATIONS AND RELEVANT DEFINITIONS

AE	Adverse Event
AR	Adverse Reaction
CA	Competent Authority
CERS	Comité d'Ethique de Recherche en Santé
CV	Curriculum Vitae
DSMB	Data Safety Monitoring Board
ELISA	Enzyme-linked immunosorbent assay
EPD	Electronic Patient Dossier
EU	European Union
EudraCT	European drug regulatory affairs Clinical Trials
GCP	Good Clinical Practice
GDPR	General Data Protection Regulation;
GP	General Practitioner
IB	Investigator's Brochure
IC	Informed Consent
IM	Intramuscular(ly)
IMP	Investigational Medicinal Product
IMPD	Investigational Medicinal Product Dossier
PBMC	Peripheral Blood Mononuclear Cells
(S)AE	(Serious) Adverse Event
SMFA	Standard Membrane Feeding Assay
SMC	Safety Monitoring Committee
SPC	Summary of Product Characteristics
Sponsor	The sponsor is the party that commissions the organisation or performance of the research, for example a pharmaceutical company, academic hospital, scientific organisation or investigator. A party that provides funding for a study but does not commission it is not regarded as the sponsor, but referred to as a subsidising party.
SUSAR	Suspected Unexpected Serious Adverse Reaction
TBV	Transmission blocking vaccine
TBA	Transmission blocking activity
TRA	Transmission reducing activity

SUMMARY

**Title**	Phase 1 Dose Escalating, Double-Blind, Randomised Comparator Controlled Trial of the Safety and Immunogenicity of Different Adjuvant Formulations of R0.6C and ProC6C transmission blocking vaccines candidates against *Plasmodium falciparum* in Adults in Burkina Faso.
**Short Title**	Phase 1 R0.6C and ProC6C transmission blocking vaccines candidates trial, Burkina Faso
**Study Acronym**	TBVax1
**Population**	Healthy Burkinabè adults
**Study Site**	Sabou health district area, Burkina Faso
**Accrual Ceiling**	250
**Total Enrolment**	125
**Study Duration**	Start Date: February 2022<br>End Date: December 2022
**Accrual Period**	Approximately 30 days
**Rationale**	Malaria, a disease caused by *Plasmodium* parasites, is one of the most important infectious diseases worldwide. After a period of success in global malaria control, progress has stalled in 2015-2018. The availability of a transmission blocking vaccine would be a critical step to move towards malaria elimination [1, 2]. The R0.6C fusion protein, consisting of the N-terminal region of Glutamate Rich Protein GLURP (R0) and the 6-cysteine C-terminal fragment of the well-established Pfs48/45 antigen (6C). The ProC6C-AlOH Vaccine is composed of ProC6C recombinant protein (0.2 mg/ml of the recombinant protein together with 1.6 mg/ml Alhydrogel®. The vaccine is supplied in sterile 2 mL Type I glass vials. Each vial contains a total volume of 0.8 mL.<br>Both are lead candidates for a transmission blocking vaccine.
**Objectives**	**Primary Objective**<br>1) To evaluate the safety and reactogenicity of R0.6C and ProC6C immunizations in healthy malaria-exposed volunteers in three doses adjuvant combinations.<br><br>**Secondary objective**<br>1) To assess the dynamics of transmission reducing activity in the standard membrane feeding assay of sera collected during and after R0.6C and ProC6C immunizations in three doses adjuvant combinations<br>2) To assess the dynamics of anti-6C antibody quantities during and after R0.6C and ProC6C immunizations in three doses adjuvant combinations<br><br>**Exploratory Objectives:**

	1) To estimate antibody levels against the immunogens and constituent antigens (e.g. R0, Pfs4845-6C, Pfs230-Pro, CSP) at all timepoints and dose adjuvant combinations.<br>2) To explore parasite, host genetics and functional antibody responses to R0.6C and ProC6C<br>3) To assess the protective efficacy against *P. falciparum* malaria infection<br>4) To assess the protective efficacy against clinical malaria episodes
**Endpoints**	**Primary endpoints:**<br>1) Incidence of serious adverse events and solicited grade 3 local and systemic adverse events (AEs) possibly, probably or definitely related to the vaccine candidate in the period from first vaccinations up to 1 month after the last immunization.<br><br>**Secondary endpoints:**<br>2) The TRA at other timepoints (2 weeks after first, second and third immunizations and 4 months post third vaccination) compared to baseline (D0) in each of the three dose-adjuvant groups.<br>3) The anti-6C antibody quantity in volunteer sera collected two weeks after each dose and at 4 months post dose 3) compared to baseline (D0) in each of the three dose-adjuvant combinations, as determined by ELISA.<br><br>**Exploratory Endpoints:**<br>4) The anti-6C antibody decay rate following R0.6C and ProC6C immunization for each of the three dose-adjuvant combinations.<br><br>5) The difference in peak anti-6C antibody quantity between the two dose groups (30 or 100µg R0.6C and 30 or 100µg ProC6C).<br><br>6) Time to *P. falciparum* malaria infection by PCR<br>7) Incidence of clinical malaria episodes defined as fever (temperature ≥38.0 by any route and/or History of fever within the last 48 hours & positive malaria smear (>0, 500, 2500, ≥5,000)
**Description of Study Design**	The study is a first-in-human phase Ib, double blind randomized controlled, dose escalation study which will include healthy, malaria exposed adults (males and females), aged 20 – 45 years.<br>Each of the study arms will receive three intramuscular vaccinations on days 0, 28, 56 with R0.6C or ProC6C adsorbed to AlOH alone, or combined with an additional adjuvant Matrix-M.<br>Dose escalation with the two adjuvant arms will be staggered starting with the lower dose group. The high dose cohorts will only start after cumulative safety

from the first two doses in the low dose cohorts have been reviewed by the DSMB. Within each dose level, enrolment of participants will be staggered. The schematic design of the study is presented in the figure below:

Figure 1: Schematic design of the study

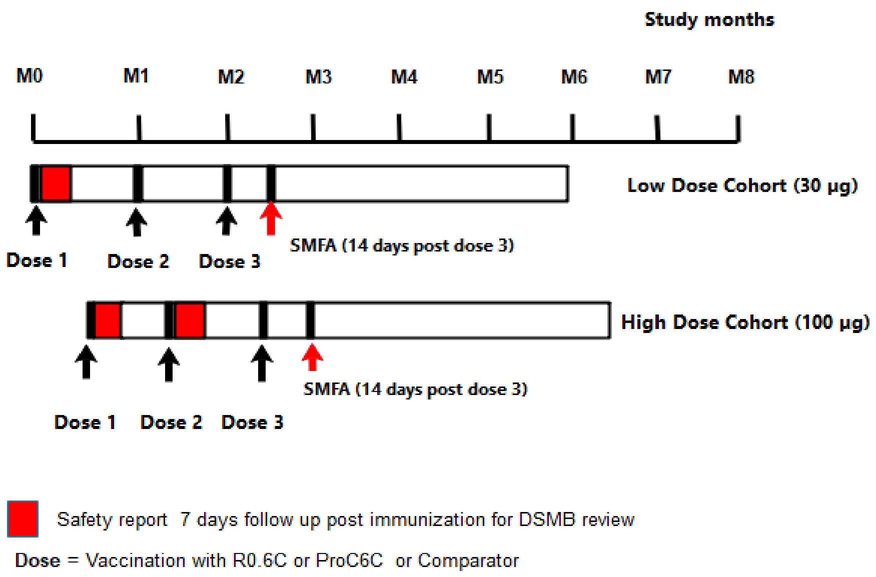

Cohort	Group	Number of volunteer	Candidate vaccine dose and adjuvant combination
Cohort 1 (low dose)	1A	n=5	3x 30µg R0.6C-AlOH
	1B	n=5	3x 30µg R0.6C-AlOH+Matrix-M
	1C	n=5	3x 30µg ProC6C-AlOH
	1D	n=5	3x 30µg ProC6C-AlOH+Matrix-M
	1E	n=5	3x Hepatitis B vaccine
Cohort 2 (high dose)	2A	n=20	3x 100µg R0.6C-AlO
	2B	n=20	3x 100µg R0.6C-AlOH+Matrix-M
	2C	n=20	3x 100µg ProC6C-AlOH
	2D	n=20	3x 100µg ProC6C-AlOH+Matrix-M
	2E	n=20	3x Hepatitis B vaccine

GO/No GO decision making

The dose escalation GO/No GO criteria will be based on safety criteria: The No GO stopping rule will be: any SAE (Serious Adverse Event) possibly, probably or definitely related to vaccination or 50% or more of subjects having a Grade 3 adverse reaction persisting at Grade 3 for > 48 hours within 7 days following each vaccine dose (day of vaccination and 7 subsequent days). The threshold of 50% corresponds to a consensus decision

	in accordance with the African Malaria Network Trust (AMANET) Malaria vaccine guidelines and will be applied for the start of the vaccination of the high dose cohort.<br>For the dose escalation to high dose cohort, a safety report will be prepared after the first vaccination of cohort 1 on the cumulative safety data collected within 7 days after the first vaccination of the low dose cohort. The blinded safety report will be directly submitted to the DSMB for their recommendation to the sponsor.<br>If any vaccinee experiences an SAE related to vaccination, then all the immunisations of the subjects in this cohort will be stopped. The vaccination will only be resumed upon the decision of the sponsor according to the recommendation of the DSMB.

1. INTRODUCTION AND RATIONALE

1.1 Introduction

Malaria is one of the most devastating infectious diseases worldwide. After a period of success in global malaria control, progress has stalled. Data from 2015-2018 highlight that no significant progress in reducing global malaria cases was made in this period. There were an estimated 228 million cases and 405 000 related deaths in 2018, mainly in children below five years of age [3]. In addition to the this burden of morbidity and mortality, this disease forms a profound economic burden for the affected countries, many of which are in any case characterised as Low and Middle Income Countries (LMIC) [4]. The urgency of the situation is further emphasized by the waning effectiveness of currently registered anti-malarials due to fast emergence and spread of resistance and the absence of a highly effective vaccine [1, 5].

Human malaria is caused by protozoa of the genus Plasmodium: *P. falciparum*, *P. vivax*, *P. ovale*, *P. malariae*, and *P. knowlesi*, of which *P. falciparum* is responsible for the greatest global burden of morbidity and mortality. Parasites (sporozoite stage) are injected into the skin by an infected female Anopheles mosquito when taking a blood meal. After penetration of the skin capillaries, sporozoites migrate to the liver, where they develop and multiply in liver cells before release into the blood (merozoite stage) and invading red blood cells for further maturation and multiplication. The cyclical proliferation of asexual stages within the human red blood cells is responsible for the occurrence of clinical symptoms. A small fraction of asexual stages commits to enter sexual development and ultimately form mature male and female gametocytes. These male and female gametocytes do not cause clinical pathology or symptoms, but can be taken up by a new Anopheline vector, in whose midgut they transform into male and female gametes, which fuse to form a zygote that in turn becomes motile and elongated (ookinete). This ookinete invades the mosquito midgut wall, developing into an oocyst which in turn releases new sporozoites. This ultimately renders the mosquito infectious upon its next bite, leading to onward transmission of malaria infections.
By interrupting transmission of malaria parasites, a reduction in the number of secondary infections in the community is expected. Malaria transmission blocking vaccines (TBVs) and transmission-blocking drugs aim to interrupt transmission to or the development of parasites in the mosquito vector by an intervention applied to the human intermediate host [5]. Deployment of transmission-blocking drugs and TBVs will be an efficient complementary element in an integrated program of anti-malarial interventions. With the implementation of TBVs we will be able to reduce the malaria burden, contain drug resistance and to move towards malaria elimination [2, 6].

The sexual stage Pfs48/45 antigen is a well-established lead candidate for a *P. falciparum* transmission blocking vaccine because of its critical role in parasite fertilisation. Male gametes lacking Pfs48/45 are unable to bind female gametes in the mosquito midgut, thus preventing ookinete, oocyst and ultimately sporozoite development. The R0.6C fusion protein is a chimera consisting of the 6-cysteine C-terminal fragment of PfS48/45 (6C) coupled to the N-terminal region of asexual stage Glutamate Rich Protein GLURP (R0) produced in *Lactococcus lactis*. Immunisation with R0.6C in rodents induces functional antibodies against the 6C subunit [7].

Anti-6C antibodies (Abs) are ingested during the blood meal and can bind male sexual forms in the mosquito gut, preventing their fertilisation of female gametes and thus ookinete and oocyst development. Sera of vaccinated animals were able to reduce transmission in the standard membrane feeding assay (SMFA) with cultured gametocytes. Anti-6C antibody titres were further increased by immunising with R0.6C adjuvanted with Alhydrogel or Alhydrogel and Matrix-M.

The SMFA is the most widely used assay to determine the TRA and an accepted surrogate of TRA for public health interventions [5]. Laboratory reared Anopheles mosquitoes are fed through membrane feeders with in vitro cultured gametocytes to which serum or purified anti-R0.6C antibodies in various concentrations are added. Subsequently, the oocyst prevalence in mosquitoes are compared between the experimental and control group by means of microscopy of the mosquito gut, parasite DNA detection or immune-assays [5, 8]. The SMFA can also be used to determine the transmission blocking activity (TBA) by comparing the number of infected mosquitoes between the experimental and control group.

Because the mode of action of TBVs is so explicit, there is a highly informative endpoint in the SMFA that is a clear indicator of later public health impact. For other vaccines, there is no such clear in vitro assay. The TRA measured in the SMFA assay around which there is confidence that blocking activity is present was set by consensus at 80% reduction in oocyst intensity, however even TBVs with a TRA of less than 80% could eliminate plasmodium at low transmission levels over consecutive transmission seasons [9].

1.2 Rationale for a transmission blocking vaccine

The renewed focus on malaria elimination has increased the priority of research towards development of interventions to block malaria transmission [9, 10]. By interrupting transmission of malaria parasites in mosquito vectors, a reduction in the number of secondary infections in the community is expected with an overall reduction in disease and mortality [11]. TBVs aim to actively induce specific and functional antibodies. Such antibodies are taken up by the mosquito during the blood meal and prevent parasite development in the mosquito gut and hence onward transmission.

The lead TBV candidate R0.6C has shown good transmission reducing efficacy in rodent models. Also, a monoclonal antibody TB31F against the same Pfs48/46 6C region has shown excellent safety and transmission reducing activity in humans in a recent clinical trial (van der Boor et al, manuscript in preparation). Efficacious TBVs would be a critical step on the path towards malaria elimination and their safety and preliminary efficacy data can be acquired in malaria-endemic countries volunteers.

2. Preclinical studies with R0.6C

2.1.1 Summary of preclinical studies with R0.6C

Preclinical studies focused on safety, immunogenicity, and functional activity of the R0.6C

vaccine candidate. **Table 1** summarizes the preclinical studies conducted in various animal species.

Table 1: Summary of Preclinical Animal Studies for R0.6C candidate Vaccines

Animal Species	Study Protocol	Material	Purpose	Dose, Route, & Regimen	Summary
Rat	Radboud UMC under approval number 2016-0020 [12]	Small scale lab-produced R0.6C	Perform dose-titration studies within anticipated dose range to demonstrate that R0.6C elicit TB antibodies and to provide data for potency evaluation.	0.03, 1.2, 4.7, 9.4, 18.9, and 37.7µg of R0.6C adjuvanted on Alhydrogel, 3 SC injections on D0, D14, and D28. Terminal bleed on SD43.	Doses are within the anticipated range for potency evaluation. Pfs48/45-6C antibody titers are associated with TRA.
Rat	MVI-01-2015/2016 under P 20-204 [7]	Up-scaled lab-produced R0.6C	Perform dose-titration studies to demonstrate that up-scaled R0.6C product elicit TB antibodies.	2.5, 10, 25µg of R0.6C adjuvanted in Montanide ISA720, 3 SC injections on D0, D21, and D42. Terminal bleed on D63.	Doses are within the anticipated range for potency evaluation. Pfs48/45-6C antibody titers are associated with TRA.
Mice	Radboud UMC under approval number 2016-0020	Up-scaled R0.6C reference material	Perform two studies within anticipated dose ranges and at the intended route of administration to confirm immunogenicity of DP formulations.	0.4, 2, 10µg of R0.6C adjuvanted on Alhydrogel or Alhydrogel + Matrix-M, 2 IM injections on D0, and D28. Terminal bleed on D63.	Both R0.6C/AlOH and R0.6C/AlOH + Matrix-M elicit high levels of functional antibodies when injected twice by the intended route of immunization.
Rabbits	CRL Study no. 78813	Tox Grade Material	Toxicity of repeated dose of R0.6C/AlOH and R0.6C/AlOH + Matrix-M	A total of 4 doses by IM, with 100 µg R0.6 with or without 50 µg Matrix-M	No systemic toxicological changes. Local test item related histopathology changes were observed at the injection sites and consisted of an up to marked focal inflammatory response. Partial recovery was recorded when comparing the site dosed 3 days prior to necropsy with the site dosed 46 days prior to necropsy

2.1.2 Immunogenicity of R0.6C/AlOH and R0.6C/AlOH + Matrix-M adjuvant

2.1.2.1 R0.6C/AlOH in rats

The preliminary study used preclinical R0.6C/Alhydrogel vaccines to determine the appropriate dose response range in rats for the products. To determine the appropriate dosing range, a

preliminary study was conducted with six groups of rats, 5 rats per group, with each animals receiving 3 SC injections on Study Days 0, 14, and 28 according to Table 9 with research grade R0.6C adjuvanted on Alhydrogel.

Table 2:: Design for rat Study of R0.6C/AlOH

Group	Number of Rats per Group	Antigen	Dose level (µg/50µL)	Adjuvant
1	5	R0.6C	0.03	Alhydrogel®
2	5		1.2	
3	5		4.7	
4	5		9.4	
5	5		18.9	
6	5		37.7	

Six groups of 5 rats were immunized on days 0, 14, and 28 by subcutaneous injection (SC) with 50 µL of the indicated formulation.

The rats were also observed for general health. The rats were terminated on Day 43, and anti-Pfs48/45 titers as well as TRA were determined. Figure 5 shows the TRA and anti-Pfs48/45 IgG levels measured by ELISA on plates coated with a gametocyte extract. In general, R0.6C elicited high levels of gametocyte-specific antibodies. At the optimal dose of 9.4 µg R0.6C, 3 out of 5 animals showed ≥90% TRA and one animal showed ≥85% TRA. Sera which exhibits a high level of TRA also has a low mosquito infection rate **(Table 3).**

There was a statistically significant correlation (Spearman rank, $p < 0.001$, $r = 0.5652$) between biological activity in SMFA and anti-gametocyte antibody levels.

Table 3: Summary of Transmission-Blocking activity of sera from rat immunized with R0.6C/AlOH

Dose		GCT	SMFA		
µg	Rat	Titre	% infected (n/N)	Mean oocysts	% TRA (95% CI)
37.7	1	644	90 (18/20)	11	58 (37.1-72.2)
	2	362	100 (20/20)	17	37 (8.1-57.0)
	3	235	100 (20/20)	16	39 (9.9-58.5)
	4	907	50 (10/20)	1.1	96 (92.5-97.7)
	5	581	55 (11/20)	1.9	93 (87.7-95.8)
18.9	1	195	100 (20/20)	19	26 (-8.7-49.4)
	2	122	100 (20/20)	15	44 (18.8-60.9)
	3	194	10 (2/20)	0.1	100 (98.4-99.9)
	4	200	95 (19/20)	24	10 (-36.1-41.0)
	5	360	95 (19/29)	22	18 (-23.3-45.1)
9.4	1	197	75 (15/20)	3.7	86 (77.3-91.7)
	2	171	85 (17/20)	8	70 (51.7-81.2)
	3	650	10 (2/20)	0.2	99 (97.8-99.7)
	4	468	90 (18/20)	2.6	90 (84.1-94)
	5	258	70 (14/20)	1.4	95 (91.2-97.1)
4.7	1	257	90 (18/20)	22.6	15 (-42.4-49.4)
	2	357	86 (18/22)	10.5	60 (38.1-74.4)
	3	138	80 (16/20)	16.4	38 (-2.7-62.9)
	4	175	95 (19/20)	15.2	43 (14.6-61.9)
	5	309	80 (16/20)	2.2	92 (96.8-95.0)
1.2	1	129	100 (20/20)	19.7	0 (-133.6- -2.8)
	2	123	95 (19/20)	12.3	1 (-59.1-38.6)
	3	527	90 (18/20)	14.7	0 (-59.1-38.6)
	4	209	95 (19/20)	16.6	0 (-96.2-28.3)
	5	511	75 (15/20)	5.2	58 (22.4-77)
0.3	1	130	100 (17/17)	19.4	0 (-144.2- -1.5)
	2	163	80 (16/20)	9.4	24 (-30.7-56.0)
	3	95	100 (19/19)	22.2	0 (-180.4- -14.0)
	4	41	93 (13/14)	13.4	0 (-96.3-40.8)
	5	17	100 (8/8)	19.8	0 (-197.5-14.6)

Individual final bleed (1:9 diluted) were tested in the SMFA. Seven days after membrane feed, the number of infected mosquitoes and oocyst density were determined. Rat: Rat serum from the R0.6Cc-immunized group; GCT: antibody titer in the gametocyte ELISA; % Infected (n/N): percentage of infected mosquitoes, n/N total infected mosquitoes/total dissected

mosquitoes; %TRA: percentage of Transmission Reducing Activity compared with the mean of the S0 samples, IC: interquartile rang; P-value: sample of the control S0 (pre-bleed serum) was used for calculation.

Figure 2: Functional activity and immune recognition of R0.6C/AlOH in rats

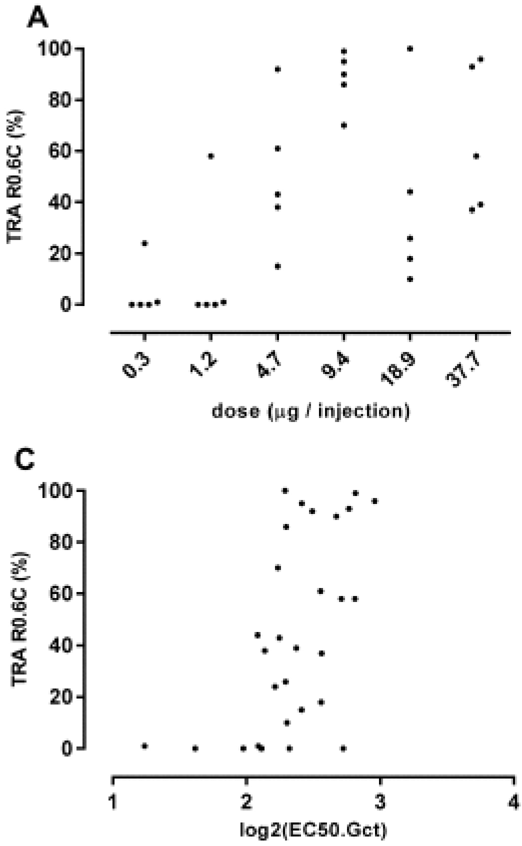

Figure 2 Functional activity and immune recognition of vaccination antigens. (**A**) Individual sera from groups of rats (*n* = 5) immunized with different doses of R0.6C in $Al(OH)_3$ were assessed for functional activity in the SMFA. (**B**) Recognition of gametocytes (●) by ELISA. Horizontal lines represent median values. (**C**) The relationship between functional activity and antibody level for individual rats is shown.

2.1.2.2 R0.6C/AlOH in Mice

Formulations were prepared to confirm immunogenicity of DP formulations. Outbreed CD-1 mice were immunized with research grade R0.6C and sera were taken on day 42 for immune analysis. Mice were immunized intramuscularly in the right thigh with 50 µl vaccine, two times on days 0 and 28 according to **Table 4.** Alhydrogel formulations contained 75 micrograms of Alhydrogel and were mixed by pipetting for 5 minutes. Matrix-MTM adjuvant (Novavax AB, Uppsala, Sweden) formulations contained 5 micrograms of Matrix-M adjuvant per injection and were mixed by pipetting for a short period of time. 70% Montanide ISA720 (Seppic, France) formulations were prepared following the manufacturer’s instructions.

Formulations that contained both Alhydrogel and Matrix-M adjuvant were prepared by first adsorbing R0.6C to Alhydrogel as described above and then adding Matrix-M adjuvant. Fourteen days after the second immunization, mice were sacrificed, and serum was collected for ELISA and SMFA analysis. **Figure 3** shows the anti-Pfs48/45 ELISA titers in individual rats and their association with TRA.

Table 4: Design for Mouse Study of R0.6C/AlOH

<table>
<tr><th>Group</th><th>Number of Mice per Group</th><th>Antigen</th><th>Dose level (µg/50µL)</th><th>Adjuvant</th></tr>
<tr><td>1</td><td>5</td><td>R0.6C</td><td>10</td><td>Montanide ISA720</td></tr>
<tr><td>2</td><td>5</td><td rowspan="3">R0.6C</td><td>0.2</td><td rowspan="3">Alhydrogel®</td></tr>
<tr><td>3</td><td>5</td><td>4</td></tr>
<tr><td>4</td><td>5</td><td>10</td></tr>
<tr><td>5</td><td>5</td><td rowspan="3">R0.6C</td><td>0.2</td><td rowspan="3">Alhydrogel®
+ Matrix-M adjuvant</td></tr>
<tr><td>6</td><td>5</td><td>4</td></tr>
<tr><td>7</td><td>5</td><td>10</td></tr>
</table>

To confirm the immunogenicity of the R0.6C DP configurations, R0.6C with Matrix-M™ adjuvant, which is a saponin-based adjuvant composed of purified saponin from the tree Quillaja Saponaria Molina [13], was evaluated in mice. Outbred CD-1 mice were immunized intramuscularly on days 0 and 28 with different dosages of R0.6C adjuvanted in either Alhydrogel® or Matrix-M adjuvant. Two weeks after the last injection, mice were bled, and antibody titers and functionality of antibodies were assessed by ELISA and the SMFA, respectively. We investigated whether addition of Matrix-M adjuvant to an Alhydrogel formulation could enhance immunogenicity in mice. 6C-specific antibody titers were significantly higher when mice were immunized with Alhydrogel/Matrix-M and 2 or 10 µg R0.6C, compared to groups that received equal amounts of R0.6C with Alhydrogel only (Figure 3A). Interestingly, pooled sera from groups that received Alhydrogel + Matrix-M and 0.4, 2 or 10 µg R0.6C reduced transmission >80% (Figure 3B). Altogether we show that a formulation of R0.6C on Alhydrogel/Matrix-M induces high levels of transmission reducing antibodies.

Figure 3: Functional activity and immune recognition of R0.6C/AlOH with or without Matrix-M adjuvant in mice

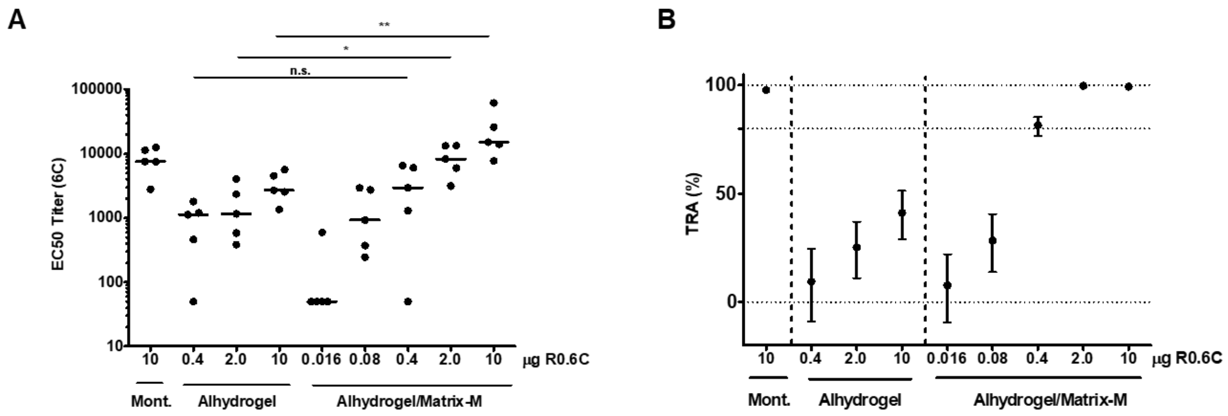

Figure 3 Immunogenicity of R0.6C vaccine formulations. Outbred CD-1 mice were immunized intramuscularly on days 0 and 28 with different dosages of R0.6C adjuvanted in Alhydrogel® with or without Matrix-M adjuvant. Two weeks after the last injection, mice were bled, and antibody titers and functionality of antibodies were assessed by ELISA and the SMFA, respectively. **A**) 6C-specific antibody titers for individual mice are shown as mid-point titers. Mid-point titers below 50 are reported as 50. Bars represent median values. Statistical difference between same-dose groups is determined by Mann-Whitney test, and reported p-values are two-sided (n.s. not significant, * $p<0.05$, ** $p<0.01$). B) Pooled sera were tested in SMFA at 1:9 dilution. Reported values and 95% confidence intervals (bars) are determined by General Linearized Mixed Models and used oocyst count data from two independent SMFA experiments with 20 mosquitoes per condition and experiment. Transmission reducing activity is calculated by comparing to a non-serum control included in each SMFA.

In summary, R0.6C /AlOH with and without Matrix-M adjuvant elicit high levels of vaccine-specific antibodies with the capacity to control parasite development in the SMFA. No toxicity in rats and mice was observed.

3. Preclinical studies with ProC6C

3.1 Summary of preclinical studies

Preclinical studies focused on safety, immunogenicity, and functional activity of the ProC6C vaccine candidate. **Table 5** summarizes the preclinical studies conducted in various animal species.

Table 5: Summary of Preclinical Animal Studies for ProC6C candidate Vaccines

Animal Species	Study Protocol	Material	Purpose	Dose, Route, & Regimen	Summary
Mice	Radboud UMC under approval number 2016-0020	Up-scaled ProC6C reference material	Perform two studies within anticipated dose ranges and at the intended route of administration to confirm immunogenicity of DP formulations.	0.4, 2, 10µg of ProC6C adjuvanted on Alhydrogel or Alhydrogel + Matrix-M, 2 IM injections on D0, and D28. Terminal bleed on D63.	Both ProC6C/AlOH and ProC6C/AlOH + Matrix-M elicit high levels of functional antibodies when injected twice by the intended route of immunization.

Rabbits	CRL Study no. 48599	Tox Grade Material	Toxicity of repeated dose of ProC6C/AlOH and ProC6C/AlOH + Matrix-M	A total of 4 doses by IM, with 100 µg R0.6 with or without 50 µg Matrix-M	No systemic toxicological changes. Local test item related histopathology changes were observed at the injection sites and consisted of an up to marked focal inflammatory response. Partial recovery was recorded when comparing the site dosed 3 days prior to necropsy with the site dosed 46 days prior to necropsy

3.2 Immunogenicity of ProC6C/AlOH and ProC6C/AlOH + Matrix-M adjuvant

3.2.1 ProC6C/AlOH in mice

Formulations were prepared to confirm immunogenicity of DP formulations. Outbreed CD-1 mice were immunized with research grade ProC6C and sera were taken on day 42 for immune analysis. Mice were immunized intramuscularly in the right thigh with 50 µl vaccine, two times on days 0 and 28 according to Table 6. Alhydrogel formulations contained 75 micrograms of Alhydrogel and were mixed by pipetting for 5 minutes. Matrix-MTM adjuvant (Novavax AB, Uppsala, Sweden) formulations contained 5 micrograms of Matrix-M adjuvant per injection and were mixed by pipetting for a short period of time. 70% Montanide ISA720 (Seppic, France) formulations were prepared following the manufacturer's instructions. Formulations that contained both Alhydrogel and Matrix-M adjuvant were prepared by first adsorbing ProC6C to Alhydrogel as described above and then adding Matrix-M adjuvant. Fourteen days after the second immunization, mice were sacrificed, and serum was collected for ELISA and SMFA analysis. Figure 4 shows the anti-Pfs48/45 ELISA titers in individual mice and their association with TRA.

Table 6: Design for Mouse Study of ProC6C/AlOH

Group	Number of Mice per Group	Antigen	Dose level (µg/50µL)	Adjuvant
2	5	ProC6C	0.2	Alhydrogel®
3	5		4	
4	5		10	
5	5	ProC6C	0.2	Matrix-M adjuvant
6	5		4	
7	5		10	
8	5	ProC6C	0.2	Alhydrogel® + Matrix-M adjuvant
9	5		4	
10	5		10	

To confirm the immunogenicity of the ProC6C DP configurations, ProC6C with Matrix-M™ adjuvant, which is a saponin-based adjuvant composed of purified saponin from the tree

Quillaja Saponaria Molina [13], was evaluated in mice. Outbred CD-1 mice were immunized intramuscularly on days 0 and 28 with different dosages of ProC6C adjuvanted in either Alhydrogel® or Matrix-M adjuvant. Two weeks after the last injection, mice were bled, and antibody titers and functionality of antibodies were assessed by ELISA and the SMFA, respectively. We investigated whether addition of Matrix-M adjuvant to an Alhydrogel formulation could enhance immunogenicity in mice. 6C-specific antibody titers were higher when mice were immunized with Alhydrogel/Matrix-M and 0.4, 2 or 10 µg ProC6C, compared to groups that received equal amounts of ProC6C with Matrix-M only (Figure 4A). Interestingly, pooled sera from groups that received Alhydrogel + Matrix-M and 0.4, 2 or 10 µg ProC6C reduced transmission >80% (Figure 4B). Altogether we show that a formulation of ProC6C on Alhydrogel/Matrix-M induces high levels of transmission reducing antibodies than ProC6C formulated on Alhydrogel, or Matrix-M only.

Figure 4: Functional activity and immune recognition of ProC6C/AlOH with or without Matrix-M adjuvant in mice

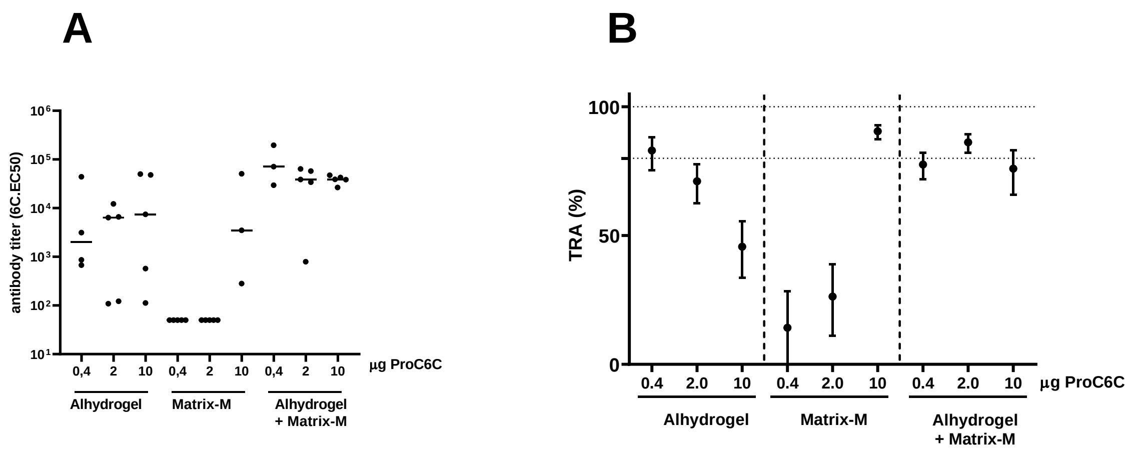

Figure 4 Immunogenicity of ProC6C vaccine formulations. Outbred CD-1 mice were immunized intramuscularly on days 0 and 28 with different dosages of ProC6C adjuvanted in Alhydrogel® with or without Matrix-M adjuvant and in Matrix-M only. Two weeks after the last injection, mice were bled, and antibody titers and functionality of antibodies were assessed by ELISA and the SMFA, respectively. **A**) 6C-specific antibody titers for individual mice are shown as mid-point titers. Mid-point titers below 50 are reported as 50. Bars represent median values. B) Pooled sera were tested in SMFA at 1:9 dilution. Reported values and 95% confidence intervals (bars) are determined by General Linearized Mixed Models and used oocyst count data from two independent SMFA experiments with 20 mosquitoes per condition and experiment. Transmission reducing activity is calculated by comparing to a non-serum control included in each SMFA.

In summary, ProC6C /AlOH with Matrix-M adjuvant consistently elicit high levels of vaccine-specific antibodies with the capacity to control parasite development in the SMFA. No toxicity in rats and mice was observed.

4. MATRIX M

Matrix-M is a saponin-based adjuvant manufactured by Novavax AB (Uppsala, Sweden). Although it is not yet fully understood how the Matrix-M adjuvant achieves its stimulatory effects, this adjuvant is known to transiently enhance the number of activated immune cells in

the draining lymph nodes which may in turn lead to increased uptake and presentation of vaccine antigens to elicit a competent immune response [14]. Specifically, it has been shown that there is an increase of CD169+ macrophages, as well as activated dendritic cells, to the draining lymph nodes after immunization with Matrix-M adjuvanted vaccines, which may help to increase antigen presentation [15]. Hence, CD169+ macrophages have previously been shown to have a role in transporting antigens to B lymphocytes by trapping them in the draining lymph node and to facilitate cross-presentation of the antigen to CD8+ T lymphocytes [16, 17]. This may lead to increased humoral and cellular immune responses, manifested by cross-reactive antibodies and multi-functional CD4+ T lymphocytes [18, 19]. Consequently, Matrix-M has been shown to contribute to antigen dose-sparing and increased duration of humoral and cellular vaccine responses.

4.1 Summary of Clinical Experience with Matrix-M

The Matrix-M adjuvant technology [20] is a promising technology which has been explored for various infectious diseases and has a good safety profile in humans [21]. To date, Matrix-M has been utilized in more than 25 clinical trials, including multiple Phase III trials (Table 7). In these studies, Matrix-M has been combined with multiple malaria vaccine candidates, influenza, COVID-19, and other disease indications. The most common dosage of Matrix-M has been 50 µg in adults, with a maximum dosage of 75 µg.

Datoo et al reported on a double-blind, randomized, controlled trial (NCT02925403) of a low-dose circumsporozoite protein-based vaccine, R21, with two different doses of adjuvant, Matrix-M (25 ug and 50 ug), in 450 children aged 5-17 months in Nanoro, Burkina Faso, a highly seasonal malaria transmission setting. Three vaccinations were administered at 4-week 39 intervals prior to the malaria season with a fourth dose one year later. R21/MM had a favorable safety profile and was well-tolerated. Vaccine efficacy (VE) was 74% (95% CI, 63-82) and 46 77% (95% CI, 67-84) in the low- and high-dose adjuvant groups, respectively. At 1 year, VE remained high at 47 77% (95% CI, 67-84) in the high-dose adjuvant group [22].

Keech et al [23] published the results of a randomized, placebo-controlled, phase 1–2 trial to evaluate the safety and immunogenicity of the rSARS-CoV-2 vaccine (in 5-µg and 25-µg doses, with or without Matrix-M1 adjuvant in 131 healthy adults (NCT04368988). The vaccine, NVX-CoV2373 appeared to be safe and was shown to elicit immune responses that exceeded levels in Covid-19 convalescent serum. The addition of Matrix-M resulted in enhanced immune responses, was antigen dose–sparing, and induced a T helper 1 (Th1) response.

Shinde et al [24] reported on rSARS-CoV-2 vaccine (containing 50 ug of Matrix-M) administered intramuscularly to adult subjects in South Africa (NCT04533399). Among 2,684 baseline seronegative participants (94% HIV-negative and 6% HIV-positive), predominantly mild-to-moderate Covid-19 developed in 15 participants in the vaccine group and in 29 in the placebo group (vaccine efficacy, 49.4%; 95% confidence interval [CI], 6.1 to 72.8). Among the vaccine recipients, the most common solicited systemic adverse events after the first dose

and second dose were headache, muscle pain, and fatigue. The mean duration of such events was slightly longer after the second dose but generally less than 3 days.

In yet another recent example (NCT01444482), an influenza vaccine containing Matrix-M was tested in a randomized, observer-blinded, active comparator-controlled trial during the 2019-2020 influenza season [25]. In brief, 2,654 clinically stable, community-dwelling adults ≥65 years of age were randomized to receive a single IM dose of either Matrix-M-adjuvanted quadrivalent nanoparticle influenza vaccine (qNIV) or a licensed inactivated influenza vaccine (IIV4). Local reactogenicity, primarily mild to moderate and transient pain, was higher in the qNIV group. qNIV was generally well tolerated and produced a qualitatively and quantitatively enhanced humoral and cellular immune response in older adults.

Table 7. Key Clinical Experience of Matrix-M with Various Vaccine Antigens.

NCT Number	Title	Conditions	Interventions	Characteristics	Population
NCT04201431	Safety, Immunogenicity and Efficacy of the Blood-stage Plasmodium Vivax Malaria Vaccine Candidate PvDBPII in Matrix M1	Malaria, Vivax	• Biological: PvDBPII/Matrix M1	Phase 1<br>Phase 2	18 Years to 45 Years (Adult)
NCT01669512	Adjuvanting Viral Vectored Malaria Vaccines With Matrix M	Malaria	• Biological: Low Dose Matrix M Regimen<br>• Biological: Standard Dose Matrix M Regimen	Phase 1	18 Years to 50 Years (Adult)
NCT04130282	VAC077: Safety and Immunogenicity of the Pfs25-IMX313/Matrix-M Vaccine	Malaria	• Biological: Pfs25-IMX313/ Matrix-M1	Phase 1	18 Years to 45 Years (Adult)
NCT04318002	Safety and Immunogenicity of RH5.1/ Matrix-M in Adults and Infants Living in Tanzania	Malaria	• Biological: RH5.1/Matrix-M	Phase 1	6 Months to 45 Years (Child, Adult)
NCT03896724	Safety, Immunogenicity and Efficacy of R21 Matrix-M in 5-17 Month Old Children in Nanoro, Burkina Faso	Malaria	• Biological: R21 adjuvanted with 25mcg Matrix-M<br>• Biological: R21 adjuvanted with 50mcg Matrix-M	Phase 1<br>Phase 2	5 Months to 17 Months (Child)
NCT04271306	Safety, Immunogenicity and ex Vivo Efficacy of Pfs25-IMX313/Matrix-M in Healthy Volunteers in Bagamoyo, Tanzania.	Malaria	• Biological: Pfs25-IMX313 (10ug)/Matrix-M (50ug)<br>• Biological: Pfs25-IMX313 (50ug)/Matrix-M (50ug)<br>• Biological: Pfs25-IMX313 (50ug)/Matrix-M (50ug) & Pfs25-IMX313 (10ug)/ Matrix-M (50ug)	Phase 1	5 Years to 45 Years (Child, Adult)
NCT02572388	A Study to Assess the Safety and Immunogenicity of the Malaria Vaccine, R21, Administered With and Without Matrix-M1	Malaria	• Biological: R21<br>• Biological: Matrix-M1	Phase 1	18 Years to 50 Years (Adult)
NCT02925403	A Study to Assess the Safety and Immunogenicity of the Malaria Vaccine, R21, With Matrix-M1 Adjuvant	Malaria	• Biological: R21/Matrix-M1<br>• Other: Saline	Phase 1<br>Phase 2	18 Years to 45 Years (Adult)
NCT01444482	Study of Parenterally Administrated Adjuvanted Seasonal Influenza Vaccine in Healthy Elderly Volunteers	Influenza	• Biological: Matrix M<br>• Biological: Seasonal influenza vaccine	Phase 1	65 Years to 75 Years (Older Adult)
NCT04611802	A Study Looking at the Efficacy, Immune Response, and Safety of a COVID-19 Vaccine in Adults at Risk for SARS-<br>CoV-2	COVID-19	• Biological: SARS-CoV-2 rS/ Matrix-M1 Adjuvant<br>• Other: Placebo	Phase 3	18 Years and older (Adult, Older Adult)
NCT04368988	Evaluation of the Safety and Immunogenicity of a SARS-CoV-2 rS Nanoparticle Vaccine With/Without Matrix-M Adjuvant	COVID-19	• Biological: SARS-CoV-2 rS - Phase 1<br>• Biological: SARS-CoV-2 rS/ Matrix-M Adjuvant, Days 0 and 21 - Phase 2	Phase 1<br>Phase 2	18 Years to 84 Years (Adult)

			• And more		
NCT04533399	A Study Looking at the Effectiveness and Safety of a COVID-19 Vaccine in South African Adults	COVID-19	• Biological: SARS-CoV-2 rS/ Matrix-M1 Adjuvant<br>• Other: Placebo	Phase 2	18 Years to 84 Years (Adult)
NCT03580824	A Study to Determine if a New Malaria Vaccine is Safe and Induces Immunity Among Kenyan Adults, Young Children and Infants	Malaria	• Biological: R21 in Matrix- M adjuvant vaccine	Phase 1<br>Phase 2	5 Months to 45 Years (Child, Adult)
NCT04583995	A Study Looking at the Effectiveness, Immune Response, and Safety of a COVID-19 Vaccine in Adults in the United Kingdom	COVID-19	• Biological: SARS-CoV-2 rS/ Matrix M1-Adjuvant<br>• Other: Placebo<br>• Biological: Licensed seasonal influenza vaccine	Phase 3	18 Years to 84 Years (Adult, Older Adult)
NCT02078674	A(H7N9) VLP Antigen Dose-Ranging Study With Matrix-M1™ Adjuvant	Influenza (Pandemic)	• Biological: Monovalent Avian Influenza VLP (H7N9)<br>• Biological: Matrix-M1™ adjuvant	Phase 1<br>Phase 2	18 Years to 64 Years (Adult)
NCT02300142	Rollover Trial for Placebo Subjects Previously Enrolled Into GEN-003-002 Study	Genital Herpes	• Biological: GEN-003 Vaccine (30-60µg of each antigen)<br>• Biological: Matrix-M2 Adjuvant (25-75µg)	Phase 2	18 Years to 50 Years (Adult)
NCT01667341	Safety and Immunogenicity Study of Therapeutic HSV-2 Vaccine	Genital Herpes	• Biological: GEN-003 with Matrix M-2<br>• Biological: GEN-003	Phase 1<br>Phase 2	18 Years to 50 Years (Adult)
NCT03026348	Safety and Immunogenicity Study to Evaluate Single- or Two-Dose Regimens Of RSV F Vaccine With and Without Aluminum Phosphate or Matrix-M1™ Adjuvants In Clinically-Stable Older Adults	Respiratory Syncytial Viruses	• Biological: RSV F Vaccine with Aluminum Phosphate Adjuvant<br>• Biological: RSV F Vaccine<br>• Biological: Matrix-M1 Adjuvant	Phase 2	60 Years to 80 Years (Adult, Older Adult)
NCT02114060	Dose Ranging Safety and Efficacy of Therapeutic HSV-2 Vaccine	Genital Herpes	• Biological: GEN-003 Vaccine (30µg of each antigen)<br>• Biological: Matrix-M2 Adjuvant (75µg)<br>• And more	Phase 2	18 Years to 50 Years (Adult)
NCT03947190	A Study to Determine if New Types of Malaria Vaccines Are Safe, Effective and Lead to Immunity in Kenyan Adults	Malaria	• Biological: R21/Matrix-M<br>• Biological: ChAd63/MVA ME-TRAP<br>• Biological: intradermal injection (ID) or direct venous injection (DVI) of PfSPZ Challenge	Phase 2	18 Years to 45 Years (Adult)
NCT03293498	Evaluation of the Safety and Immunogenicity of a Recombinant Trivalent Nanoparticle Influenza Vaccine With Matrix M-1 Adjuvant (NanoFlu)	Influenza	• Biological: NanoFlu<br>• Biological: Fluzone HD - Day 0<br>• Biological: Fluzone HD - Day 21<br>• Other: Saline - Day 21	Phase 1<br>Phase 2	60 Years and older (Adult, Older Adult)
NCT03658629	Phase 2 Dose and Formulation Confirmation of Quad-NIV in Older Adults	Influenza, Human	• Biological: NanoFlu (Quad-NIV)<br>• Other: Matrix-M Adjuvant<br>• Biological: Fluzone HD<br>• Biological:Flublok	Phase 2	65 Years and older (Older Adult)
NCT03970993	VAC 072-An Efficacy Study of R21/MM in Different Dose Schedules	Malaria	• Biological: R21 Matrix-M vaccination<br>• Biological: R21 Matrix-M vaccination and CHMI	Phase 1<br>Phase 2	18 Years to 45 Years (Adult)
NCT02370589	Study to Evaluate the Immunogenicity and Safety of an Ebola Virus (EBOV) Glycoprotein (GP) Vaccine in Healthy Subjects	Ebola	• Biological: Base Dose EBOV GP Vaccine<br>• Biological: 2-8x Base Dose EBOV GP Vaccine<br>• Biological: Matrix-M Adjuvant	Phase 1	18 Years to 50 Years (Adult)
NCT02515175	Evaluating New Formulation of Therapeutic HSV-2 Vaccine	Genital Herpes	• Biological: Matrix-M2<br>• Biological: GEN-003	Phase 2	18 Years to 50 Years (Adult)
NCT04120194	Phase 3 Pivotal Trial of NanoFlu™ in Older Adults	Influenza, Human	• Biological: NanoFlu<br>• Biological: Fluzone Quadrivalent	Phase 3	65 Years and older (Older Adult)

NCT04645147	Safety and Immunogenicity of an Epstein-Barr Virus (EBV) gp350-Ferritin Nanoparticle Vaccine in Healthy Adults With or Without EBV Infection	EBV	• Biological: EBV gp350- Ferritin Vaccine<br>• Other: Matrix-M1	Phase 1	18 Years to 29 Years (Adult)
NCT03146403	Maintenance Dose Study of GEN-003 in Subjects With Genital Herpes Infection	Genital Herpes	• Biological: GEN-003<br>• Biological: Matrix-M	Phase 2	Child, Adult, Older Adult
NCT02905019	A Safety and Efficacy Study of R21 +/- ChAd63/MVA ME-TRAP	Malaria	• Biological: R21 with Matrix- M1<br>• Biological: ChAd63 ME-TRAP<br>• Biological: MVA ME-TRAP	Phase 1<br>Phase 2	18 Years to 45 Years (Adult)

5. OBJECTIVES

5.1 Primary Objective:

1) To evaluate the safety and reactogenicity of R0.6C and ProC6C immunizations in healthy malaria-exposed volunteers in three doses adjuvant combinations.

5.2 Secondary objectives;

1) To assess the dynamics of transmission reducing activity in the standard membrane feeding assay of sera collected during and after R0.6C or ProC6C immunizations in three doses adjuvant combinations
2) To assess the dynamics of anti-6C antibody quantities during and after R0.6C or ProC6C immunizations in three doses adjuvant combinations

5.3 Exploratory Objectives:

1) To estimate antibody levels against the immunogens and constituent antigens (e.g. R0, Pfs4845-6C, Pfs230-Pro, CSP) at all timepoints and dose adjuvant combinations.
5) To explore parasite, host genetics and functional antibody responses to R0.6C and ProC6C
6) To assess the protective efficacy against *P. falciparum* malaria infection
7) To assess the protective efficacy against clinical malaria episodes

6. STUDY DESIGN

This is a first-in-human phase Ib, double blind, single site, randomized controlled, dose escalation study to determine the safety tolerability and immunogenicity of the R0.6C and ProC6C, adsorbed to AlOH alone, or combined with an additional adjuvant Matrix-M.

It is two parallel cohorts:

Cohort 1: 25 subjects (5 subjects receiving 30 µg of R0.6C vaccine adsorbed to AlOH alone; 5 subjects receiving 30 µg of R0.6C vaccine adsorbed to AlOH+Matrix-M, 5 subjects receiving 30 µg of ProC6C vaccine adsorbed to AlOH alone; 5 subjects receiving 30 µg of ProC6C vaccine adsorbed to AlOH+Matrix-M, and 5 subjects receiving Hepatitis B vaccine).

Cohort 2 ; 100 subjects (20 subjects receiving 100 µg of R0.6C vaccine adsorbed to AlOH alone; 20 subjects receiving 100 µg of R0.6C vaccine adsorbed to AlOH+Matrix-M, 20 subjects receiving 30 µg of ProC6C vaccine adsorbed to AlOH alone; 20 subjects receiving 30 µg of ProC6C vaccine adsorbed to AlOH+Matrix-M, and 20 subjects receiving Hepatitis B vaccine).

Immunization schedule will be on days 0, 28, 56 for all cohorts and provisionally as following for each cohort:

- Study days 0, 28 and 56 for cohort 1

- Study days 56, 84 and 112 for cohort 2

Dose escalation with the two adjuvant arms will be staggered starting with the lower dose cohort (30µg). The high dose (100µg) cohort will only start after cumulative safety from the first dose in the low dose cohort have been reviewed by the DSMB. Second and Third immunisation of the cohort 2 will start after the review of the cumulative safety data of 7 days after immunization 1 and 2 of the cohort 2 respectively. Within each dose level, enrolment of participants will be staggered

Administration of vaccines to volunteers and follow up will take place in the Centre Medical Saint Maximilien Kolbe at Sabou.

Randomization will be done for each cohort at the times of first vaccinations.

Route of inoculation will be intramuscular in the deltoid muscle on alternating sides.

Total trial duration is approximately 6 months for each subject.

Each subject will be observed for

- At least 60 minutes after vaccination to evaluate and treat any acute adverse events (AEs).
- Seven (7) day follow-up period for solicited local and systemic adverse events (day of vaccination plus 6 subsequent days).
- Eighty four (84) day follow-up period for unsolicited adverse events.
- Follow-up of serious adverse events (SAE's) for 6 months after the first dose of study vaccine (4 months after dose 3).

Venous blood samples will be collected to assess the functional transmission reducing activity and immunogenicity at pre-specified time points after the vaccinations as secondary and exploratory objectives (also see section 2).

Table 8: . Subjects of each cohort

**Cohort**	**Group**	**Number of volunteers**	**Candidate vaccine dose and adjuvant combination**
Cohort 1 (low dose)	1A	n=5	3x 30µg R0.6C-AlOH
	1B	n=5	3x 30µg R0.6C-AlOH+Matrix-M
	1C	n=5	3x 30µg ProC6C-AlOH
	1D	n=5	3x 30µg ProC6C-AlOH+Matrix-M
	1E	n=5	3x Hepatitis B vaccine
Cohort 2 (high dose)	2A	n=20	3x 100µg R0.6C-AlO
	2B	n=20	3x 100µg R0.6C-AlOH+Matrix-M
	2C	n=20	3x 100µg ProC6C-AlOH
	2D	n=20	3x 100µg ProC6C-AlOH+Matrix-M
	2E	n=20	3x Hepatitis B vaccine

Figure 5: Schematic design of the study

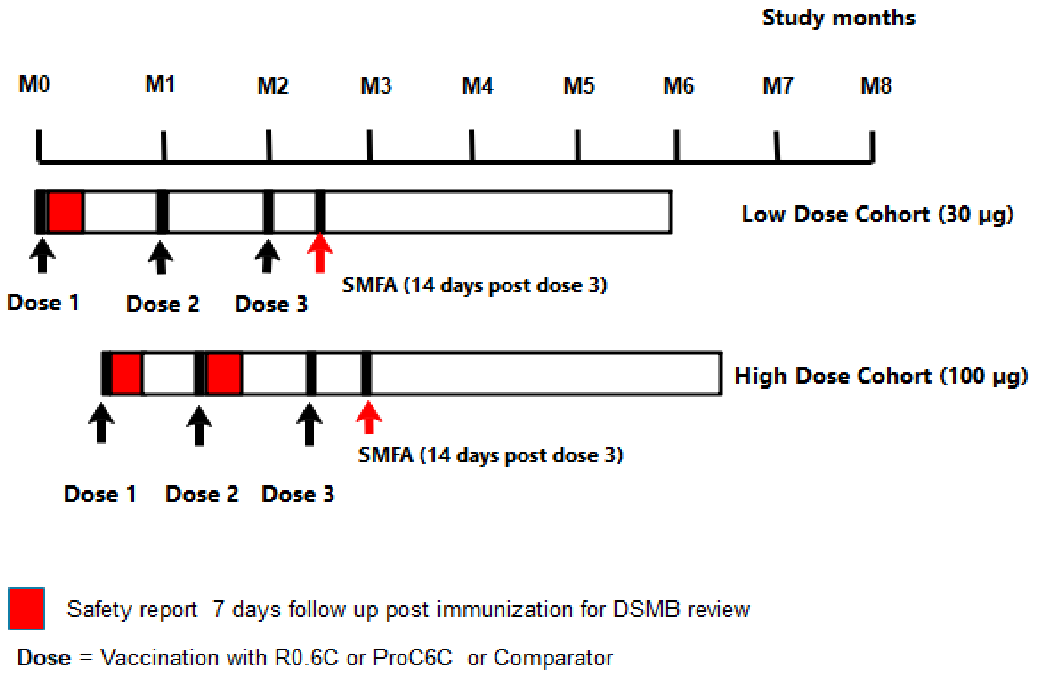

STUDY POPULATION

6.1 Site

The study will be conducted by the GRAS at the Sabou health district (SHD) area located about 100 kms directly to the west of Ouagadougou, the capital city of Burkina Faso. The SHD covers an area of 449 km^2 and is in the region of Boulkiemde with 112 485 inhabitants, according to the district health records, with most people living in small rural villages in houses made with mud or cement walls and thatched or metal roofs. The population is stable and mainly composed with the Mossi ethnic group. Farming is the main activity.

The study's field station is based at Sabou town which is the main town in the district. The SHD include 20 peripheral heath facilities which represent the primary point of contact with the health system for the population. It and is in a sudan sahelian eco-climatic zone. The climate consists of a single rainy season from May to October followed by a long dry season. This defines the highly seasonal malaria transmission with most malaria episodes experienced during or immediately following the rainy season.

6.2 Population

The study population will be comprised of adult male and female healthy subjects aged 20-45 at time of the first administration. A total of 125 subjects (cohort 1=25, cohort 2=100) will be enrolled to participate in the study. The investigator will ensure that all subjects being considered for the study meet the eligibility criteria described below. Subject eligibility is to be established and confirmed by checking all inclusion/exclusion criteria at both screening and inclusion (baseline). A relevant record of the eligibility criteria will be stored with the source documentation at the study site.

6.3 Inclusion criteria

In order to be eligible to participate in this study, a subject must meet all of the following criteria:

1. Subject must sign/thumbprint written informed consent to participate in the trial.
2. Subject is a male or non-pregnant and non-lactating female age ≥ 20 and ≤ 45 years and in good health.
3. Subject is able to understand planned study procedures and demonstrate comprehension of the protocol procedures
4. In the opinion of the investigator, the subject can and will comply with the requirements of the protocol.
5. Subjects are available to attend all study visits and are reachable by phone throughout the entire study period from day -28 until day 180 (end of study).
6. The subject will remain within reasonable travelling distance from the study site from day 0 until day 7 after each administration.
7. The subject agrees to refrain from blood donation throughout the study period.
8. Female subjects of non-childbearing potential may be enrolled in the study. Non-childbearing potential is defined as current bilateral tubal ligation or occlusion, hysterectomy, bilateral ovariectomy or post-menopause. All subjects of childbearing potential must agree to use continuous adequate contraception* until 2 months after completion of the study. Female subjects must agree not to breastfeed from 30 days prior to administration until 2 months after completion of the study. Female subject must have a negative pregnancy test at the inclusion visit.

**Acceptable forms of female contraception include: established use of oral, injected or implanted hormonal contraceptives; intrauterine device or intrauterine system; barrier methods (condoms or diaphragm with additional spermicide); male partner's sterilization (with appropriate post-vasectomy documentation of absence of sperm in the ejaculate); true abstinence when this is in line with the preferred and usual lifestyle of the subject. Periodic abstinence (e.g., calendar, ovulation, symptothermal, post-ovulation methods) and withdrawal are not acceptable methods of contraception. Adequate contraception does not apply to subjects of child bearing potential with partners of the same sex.*

6.4 Exclusion criteria

A potential subject who meets any of the following criteria will be excluded from participation in this study:

1. Acute or chronic disease at time of administration, clinically significant pulmonary, cardiovascular, hepatic, renal, neurological or immunological functional abnormality, as determined by medical history, physical examination or laboratory screening tests:
   a. Acute disease is defined as the presence of a moderate or severe illness with or without fever. For subjects with an illness on the day of immunization, the vaccination may be postponed up to 7 days.
   b. Fever is defined as temperature ≥ 38.0°C by any route (axillary, oral, tympanic or

forehead) according to the standard definition of the Brighton collaboration [26] .

c. Hemoglobin, white blood cell (WBC), absolute neutrophil count, or platelet levels outside the local laboratory–defined limits of normal. (Subjects may be included at the investigator's discretion for "not clinically significant" values outside of normal range and ≤ Grade 2.)

d. Alanine transaminase (ALT) or creatinine (Cr) level above the local laboratory–defined upper limit of normal. (Subjects may be included at the investigator's discretion for "not clinically significant" values outside of normal range and ≤ Grade 2.)

2. History of malignancy of any organ system (other than localized basal cell carcinoma of the skin), treated or untreated, within the past 5 years.
3. History of auto immune disease
4. Subjects with splenectomy
5. Chronic use of i) immunosuppressive drugs, ii) or other immune modifying drugs within three months prior to study onset (inhaled and topical corticosteroids and oral anti-histamines exempted) or expected use of such during the study period.
6. History of drug or alcohol abuse interfering with normal social function in the period of one year prior to study onset.
7. Known history of Human Immunodeficiency Virus (HIV) (No test will be done by the study)
8. Screening tests positive for Hepatitis B Virus (HBV), Hepatitis C Virus (HCV).
9. Use of any other investigational or non-registered product (drug or vaccine) during the study period.
10. Participation in any other clinical study involving an investigational product in the 30 days prior to the start of the study or during the study period.
11. Receipt of any other vaccination within 30 days prior to the first vaccination.
12. Any previous participation in any malaria (vaccine) study.
13. History of anaphylaxis or known severe hypersensitivity to any of the vaccine components (adjuvant or antigen or excipient)
14. Any other condition or situation that would, in the opinion of the investigator, place the subject at an unacceptable risk of injury or render the subject unable to meet the requirements of the protocol.

6.5 Sample size assumptions

The sample size is determined to enable broad initial estimates of the incidence of local and general side effects possibly related Grade 3 adverse events (AE) or serious adverse events (SAE). In this study, we are primarily concerned with serious adverse events, which theoretically could occur as a result of immunization. The rate of such serious adverse events is not initially known. As the number of subjects studied increases, as long as no such events are observed, confidence that the true rate of any SAEs is below a certain level grows

progressively. The tables below provide four different theoretical SAE rates, ranging from 1 in 100 (0.01) to 1 in 10 (0.1), and the associated probability of observing no serious adverse events or at least one serious adverse event in the study. For example, if the SAE rate is 8%, then the probability of observing at least one event in a group of size 18 (Pr ≥1/18) is 0.81, while the probability of observing no event in a group of size 18 (Pr 0/18) is 0.19.

Table 9: Event detection probabilities for groups of size 20

Event Rate	Pr 0/18	Pr ≥1/18
0.01	0.82	0.18
0.05	0.36	0.64
0.08	0.19	0.81
0.10	0.12	0.88

Event Rate: true rate at which an event occurs.
Pr 0/18: Given the event rate, probability that no events will be detected among 18 vaccinees.
Pr ≥1/18: Given the event rate, probability that one or more events will be detected among 18 vaccinees.

Therefore for cohort 2, considering a drop of rate of 10% we will recruit 20 participants per group i.e 80 participants in total who will receive the malaria vaccines candidates and 20 controls (100 participants in total for cohort 2).
No formal sample size calculation was performed for cohort 1. We will recruit 5 participants for each of the 5 arms i.e 25 participants in total for cohort 1.

6.6 Withdrawal of individual subjects

Subjects can leave the study at any time for any reason if they wish to do so without any penalty or loss of medical benefits. Subjects can also be withdrawn from the study procedures at the discretion of the clinical investigator or the local safety monitor for urgent medical reasons or if exclusion criteria are met. The following reasons may lead to withdrawal of individual subjects:

- Withdrawal of informed consent by volunteer;
- Any serious adverse event;
- Any adverse event that, according to clinical judgment of the investigator, is considered as a definite contraindication to proceeding with the study procedures;
- Immunosuppressant or other immune-modifying drugs administered chronically (i.e., more than 14 days in total) during the study period. Topical steroids are allowed;
- Immunoglobulin and/or any blood products administered during the study period;
- Pregnancy;
- Completely lost to follow-up;
- Ineligibility (arising during the study or retrospectively, having been overlooked at screening);
- If the investigator or safety monitor believes that continuation would be detrimental to the subject's well-being;
- Volunteer non-compliance with study requirements;
- Any other protocol deviation that results in a significant risk to the subject's safety.

For subjects lost to follow-up (i.e. those subjects whose status is unclear because they fail to appear for study visits without stating an intention to withdraw), extensive effort (i.e.

documented phone calls) will be undertaken to locate or recall the volunteer or at least to determine his or her health status. The investigator should show "due diligence" by documenting in the source documents steps taken to contact the subject. In case of premature withdrawal for any reason, the investigator will exert his/her best effort to:

- Update any ongoing AE/SAEs that remained ongoing at the time of the subject's last visit prior to withdrawal.
- Determine if the subject has had any reaction or AE since the last visit. Where possible, the investigator should visibly or physically assess any reported adverse reaction or AE and document whether it led to the withdrawal.
- Collect blood for biochemical and hematological clinical laboratory parameters.
- Review the subject's memory aid if it is still in use at the time of withdrawal.
- Document the reason for premature withdrawal on the CRF.

6.7 Replacement of individual subjects after withdrawal

If an assigned participant does not present on the day of first administration or elects to withdraw the consent on the day of administration, one of the reserve participants will replace the participant. After participants have received administration they cannot be replaced.

6.8 Follow-up of participants withdrawn from treatment

A subject may end his or her participation in the study and still be followed up for safety, unless the subject chooses to have complete withdrawal of the consent for further participation in any trial procedures. If a subject chooses to withdraw from the study, the investigator will make a reasonable effort to determine the reason for the subject's withdrawal and complete the study termination CRF.

6.9 Premature termination of the study

The study may be discontinued by the sponsor:

- On advice of the safety monitor
- On advice of the Data Safety Monitoring Board (DSMB)
- On advice of the clinical investigator
- On advice of the ethical committee/regulatory authority

The investigators, local safety monitor, DSMB, ethical committee, regulatory or Sponsor may decide to put the study on hold based on adverse events, pending discussion with the Sponsor, DSMB, ethical committee, regulatory authority, local safety monitor or investigators. Following discussion, it may be decided to terminate the study.

7. INVESTIGATIONAL PRODUCTS

7.1 The malaria vaccine candidates

7.1.1 Name and description of investigational product(s)

7.1.1.1 The R0.6C

The R0.6C fusion protein is a chimera consisting of the 6-cysteine C-terminal fragment of PfS48/45 (6C) coupled to the N-terminal region of asexual stage Glutamate Rich Protein

GLURP (R0) produced in Lactococcus lactis [7, 12, 27]. The carrier protein R0 helps to enhance the immune response against Pfs48/45 [28].

The recombinant R0.6C protein is formulated at 200 ug/m in 10 mM HEPES, 2.5% glucose, 0.5 mM EDTA, 155 mM NaCl and absorbed to 1.6 mg/mL Alhydrogel [Al(OH)3], at a fill volume of 0.8 mL in a 2 mL borosilicate glass vial and stored at 2-8°C.

7.1.1.2 The ProC6C

The ProC6C fusion protein is a chimera consisting of the 6-cysteine C-terminal fragment of PfS48/45 (6C) coupled to the Pro domain of Pfs230 produced in *Lactococcus lactis* (Singh et al. 2021, manuscript submitted). ProC6C is a recombinant hybrid protein derived from Plasmodium falciparum 3D7, Pfs230Pro (Pro) genetically coupled to *P. falciparum* Pfs48/45 (6C).The hybrid protein consists of the $Pro_{443–590}$ and $Pfs48/45_{291-428}$ regions joined by a linker region, $PfCSP_{105-140}$, and the vector-encoded amino acid residues A-E-R-S at the N-terminal end and a C-tag (E-P-E-A) in its C-terminus

The ProC6C-AlOH Vaccine is composed of ProC6C recombinant protein (0.2 mg/ml of the recombinant protein together with 1.6 mg/ml Alhydrogel®. The vaccine is supplied in sterile 2 mL Type I glass vials. Each vial contains a total volume of 0.8 mL

7.1.1.3 The adjuvant Matrix-M

Matrix-M™ is a saponin-based product produced using the matrix adjuvant technology patented by Isconova (now owned by Novovax). Matrix-M1 is a novel adjuvant designed to stimulate both humoral and cellular immune responses to vaccines. Matrix-M1 is formed from selected purified saponin fractions formulated separately into different Matrix particles, Matrix A and Matrix C. Matrix-M1 is constituted from a mixture of Matrix A and Matrix C in a ratio of 85:15.

Matrix M™ has previously been used to adjuvant an intranasal DNA vaccine, leading to improvements in local antibody responses, and increased expression of Th1 and Th2 cytokines. Matrix M™ significantly enhanced antibody responses to a commercial trivalent seasonal influenza vaccine. Mixed with a virosomal H9N2 avian influenza vaccine, Matrix-M1 induced enhanced antigen-specific humoral and CD8+ T cell response. Matrix M™ administered with an intramuscular H5N1 virosomal influenza vaccine induced a strong immediate and long-term humoral and cellular immune response and showed a dose-sparing potential.

Matrix-M is formulated as 750µL per vial at a concentration of 375 ug/mL.

7.1.2 Storage of Investigational Medicinal Product

Vials of R0.6C-AlOH and ProC6C-AlOH must be stored at 2ºC to 8ºC. Freezing destroys the integrity of aluminium hydroxide suspensions. Thus, any vials that have been frozen must not be used for administration to humans.

Vials of Matrix-M must also be stored at 2ºC to 8ºC. Any vials that have been frozen must not be used for administration to humans.

7.1.3 Preparation and labelling of Investigational Medicinal Products

For those receiving R0.6C: In cohort 1, a volume of 150µL of R0.6C/ AlOH will be aspired from the vial for the dose of 30µg R0.6C and a volume of 500µL will aspired for the dose of 100µg R0.6C (Cohort 2).

For those receiving ProC6C: In cohort 1, a volume of 150µL of ProC6C/ AlOH will be aspired from the vial for the dose of 30µg ProC6C and a volume of 500µL will aspired for the dose of 100µg ProC6C (Cohort 2).

For those receiving R0.6C/ AlOH adjuvanted with Matrix-M: 210 µL of Matrix-M will be added to each vial containing 800µL of R0.6C/ AlOH and mixed by inversion by hand ten-times. A volume of 190µL of the premixed R0.6C adjuvanted with AlOH + Matrix-M will be aspired for administration of the 30µg R0.6C (15 µg Matrix-M) (Cohort 1) and a volume of 630µL will be aspired for administration of the 100µg R0.6C dose (49 µg Matrix-M) (Cohort 2).

For those receiving ProC6C/ AlOH adjuvanted with Matrix-M: 210 µL of Matrix-M will be added to each vial containing 800µL of ProC6C/ AlOH and mixed by inversion by hand ten-times. A volume of 190µL of the premixed ProC6C adjuvanted with AlOH + Matrix-M will be aspired for administration of the 30µg ProC6C (15 µg Matrix-M) (Cohort 1) and a volume of 630µL will be aspired for administration of the 100µg ProC6C dose (49 µg Matrix-M) (Cohort 2).

Figure 6: Schematic of R0.6C reconstitution and dosing

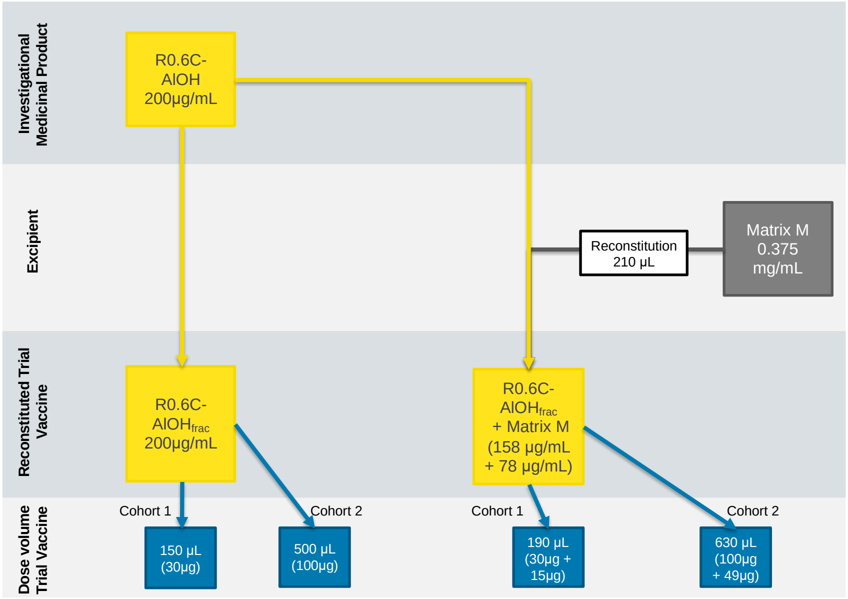

Figure 7: Schematic of ProC6C reconstitution and dosing

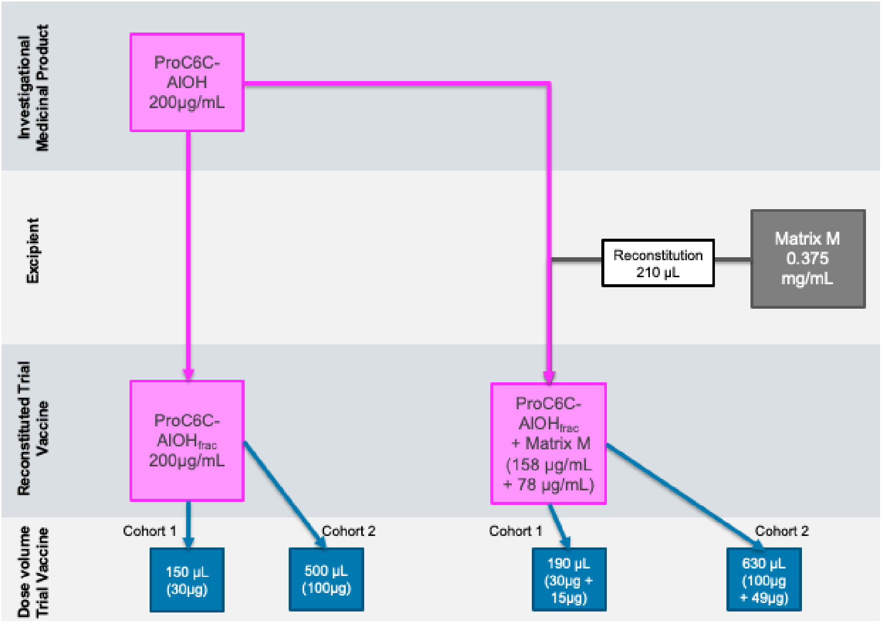

Example of label for R0.6C-AlOH:

200 µg R0.6C - 1.60 mg Al(OH)3/mL for<br>reconstitution and IM injection<br>Single Use according to Pharmacy Manual<br>For clinical trial use only<br>Store cold at 2°C to 8°C<br>For Clinical Trial Use Only<br>**Batch: xxxxxx**

Example of label for ProC6C-AlOH:

200 µg ProC6C - 1.60 mg Al(OH)3/mL for<br>reconstitution and IM injection<br>Single Use according to Pharmacy Manual<br>For clinical trial use only<br>**Store cold at 2° to 8°C**<br>**For Clinical Trial Use Only**<br>**Batch: xxxxxx**

Example of label for Matrix-M:

0.75mL / bottle Art. no: 30-110-614 03<br>Matrix-M1 Adjuvant 0.375 mg / mL<br>Mix with antigen, for IM use<br>Do not use after: 2022-05<br>Batch nr: M1-118 Manufacturer: Novavax AB<br>For Clinical Trial Use Only

7.1.4 Dosages, dosage modifications and method of administration

Volunteers will sequentially receive three administrations of R0.6C, ProC6C or Hepatitis B intramuscularly in the deltoid muscle on alternating sides on days 0, 28 and 56.

In the low dose cohort, participants will receive three times 30µg of R0.6C-AlOH or R0.6C-AlOH adjuvanted with 15 µg Matrix-M or 30µg of ProC6C-AlOH or ProC6C-AlOH adjuvanted with 15 µg Matrix-M.

In the high dose cohort, participants will receive 100µg of R0.6C-AlOH or R0.6C-AlOH adjuvanted with 49 µg Matrix-M or 100µg of ProC6C-AlOH or ProC6C-AlOH adjuvanted with 49 µg Matrix-M. In either cohort, controls will receive Hepatitis B vaccine.
Each enrolled subject will receive a total of three vaccinations. The first vaccination will take place on day 0, the second on day 28 (+/- 2 days) and the third on day 56 (+/- 2 days). The vaccination will be prepared by study pharmacist and will be administered intramuscularly in the deltoid muscle on alternating sides by the study nurse. The site of injection will be recorded on the case report form.

7.1.5 Shipment and Receipt

The vaccines will be shipped to the trial centre according to the pre-determined schedule which will be based on the estimated study start date, and only after the written Burkinabe ethics approval for the trial and import licenses has been received by the sponsor's representative. At the clinical trial site, a staff member (investigator, designated site staff or pharmacist) will be in charge of product management and will be responsible for receipt, storage and accountability of the products. The person in charge of investigational product management will be expected to return to the sponsor's representative a completed dispatch note, which will be attached to the package, as acknowledgement of receipt. The person in charge of product receipt will check that the cold chain was maintained during shipment. In case of a problem, (s)he should alert the clinical trial monitor (and sponsor's representative) immediately. The acknowledgement of receipt will be dated and signed by the person in charge of product management. A scanned copy is sent to the sponsor's representative.

7.1.6 Accountability and Return of Product

In accordance with all applicable regulatory requirements, the person in charge of product management must keep up to date the following inventory records:

- Receipt of the product at the clinical trial site
- Vaccine administered to each subject (including treatment numbers)
- Inventory of the product at the clinical trial site
- Log of any unused / expired investigational product.

These records should include dates, quantities, batch numbers, period of use/expiration dates (if applicable), and any unique code numbers assigned to the product and /or subjects.
These records should document adequately that:

- The subjects were provided the doses specified by the protocol
- All products provided are fully reconciled.

These records will be monitored and verified by the clinical trial monitor regularly, as part of the routine monitoring procedure. Unused and/or open products will be either returned to the sponsor representative or designee at the end of the vaccination period together with the form "Return of unused/or open products" or destroyed in accordance with the sponsor's representative's instructions.

7.2 The Hepatis B vaccine

ENGERIX-B [Hepatitis B Vaccine (Recombinant)] is a non-infectious recombinant DNA hepatitis B vaccine developed and manufactured by GlaxoSmithKline Biologicals. It contains purified surface antigen of the virus obtained by culturing genetically engineered Saccharomyces cerevisiae cells, which carry the surface antigen gene of the hepatitis B virus. The surface antigen expressed in Saccharomyces cerevisiae cells is purified by several physicochemical steps and formulated as a suspension of the antigen adsorbed on aluminium hydroxide.

Each 1-mL adult dose contains 20µg of hepatitis B surface antigen adsorbed on 0.5 mg aluminium as aluminium hydroxide.

The vaccine should be stored refrigerated between 2° and 8°C and should not be frozen. It should be discarded if product has been frozen.

In case of unavailability, another licensed hepatitis vaccine may be used.

7.3 Indications for deferral of vaccination

The following constitute contra-indications to administration of vaccine at that point in time. If any one of these occurs at the time scheduled for vaccination, the subject may be vaccinated at a later date or withdrawn, at the discretion of the PI. If necessary, medical care, including inpatient care, will be offered.

- Acute disease at the time of vaccination. Acute disease is defined as the presence of a moderate or severe illness with or without fever. All vaccines can be administered to persons with a minor illness (such as diarrhoea or mild upper respiratory infection without fever, i.e. temperature < 38.0 °C) at the discretion of the investigators. Details of any minor illness will be recorded in the CRF.
- Body temperature of ≥ 38.0°C at the time of vaccination.
- Receipt of another vaccination within two weeks of study vaccine.

7.4 Absolute contraindication for subsequent vaccination

A subject will be withdrawn from receiving further immunisations in the following situations:

- Occurrence of an acute allergic reaction (significant IgE-mediated events) or anaphylaxis
- Occurrence of any adverse event that according to clinical judgment of the investigator, is considered as a definite contra-indication to proceeding with the vaccinations.
- Occurrence of 2 or more Grade 3 adverse events in one volunteer, related to the immunisation and persisting at grade 3 for more than 48 hours at any day during the 28 days follow-up after an immunisation
- Signs of necrosis at the site of the injection
- Any confirmed or suspected immunosuppressive or immunodeficient condition, including human immunodeficiency virus (HIV) infection

- The use of concomitant, chronic medication (defined as more than 14 days) active on the immune system (steroids or other immunosuppressive agents) except topical and inhaled steroids
- Pregnancy during the trial period.

7.5 Concomitant medication/vaccination

At each study visit, the investigator should question the enrolled volunteer about any medication(s) taken.

All antipyretic, analgesic and antibiotic drugs, administered at any time during the period starting with administration of each dose and ending 28 days after each dose of vaccine are to be recorded with generic name of the medication (trade names are allowed for combination drugs), medical indication, total daily dose, route of administration, start and end dates of treatment.

Any immunoglobulin, blood products and any immune modifying drugs administered at any time during the study period are to be recorded with generic name of the medication (trade names are allowed for combination drugs only), medical indication, total daily dose, and route of administration, start and end dates of treatment.

Any vaccine not foreseen in the study protocol administered during the volunteer participation in the trial is to be recorded with the trade name, route of administration and date(s) of administration.

Any concomitant medication administered as prophylaxis in anticipation of reaction to the vaccination must be recorded in the CRF with generic name of the medication (trade names are allowed for combination drugs only), total daily dose, route of administration, start and end dates of treatment and coded as ‘Prophylactic’

8. METHODS

8.1 Community sensitisation

The trial team will hold local community meetings and explain the trial to potentially eligible adults. During these meetings the investigators will explain the following: the need for a vaccine (including a simple picture of the burden of malaria on the community in narrative detail); the current status of vaccine development (including the fact that this is likely to be a prolonged process); the trial screening and informed consent procedure; risks of vaccination and the unproven benefits of vaccination. It will be stressed that this is an experimental vaccine and is not designed to provide a direct protection to the vaccinee. It necessary to seek treatment for possible malaria even after vaccination and continue to use other protective measures such as bednets. It will be made clear that neither participants, nor investigators will know which vaccination regimen the subject has received until the end of the trial. It will be explained that a photograph will be taken if they are eligible to be enrolled in the trial, to aid identification.

After this meeting volunteering to participate in the study will be invited to the trial site for the screening visit. If the number of volunteers is large in relation to the size of the study sample, a public draw will be made to select the subjects to participate in the screening visit.

8.2 Randomisation, blinding and treatment allocation

This will be a double-blind trial to enable appropriate safety evaluation prior to proceeding to the next dose group. The time of vaccine administration will be documented in the case report form (CRF).

A randomisation list will be generated, and sequentially numbered opaque sealed envelopes will be made for the investigators to ensure treatment concealment.

At each cohort, randomization will be used for the allocation to one of the different adjuvant combinations or control vaccine. The volunteers will be allocated to one of the two adjuvant arms per group at random according to a randomization list prepared by an independent statistician. Stratification will not be performed. An independent pharmacist is responsible for performing the randomization and for assigning volunteers according to the randomization list. A pharmacist assistant, not involved in the assignment of volunteers, will check to see if randomization is done correctly.

Double-blinding in this context means that the vaccine recipient, all investigators and the study team responsible for the evaluation of safety, efficacy and immunogenicity endpoints will all be unaware of the vaccine receive. The vaccines will be different in terms of volume and appearance. Therefore, the contents of the syringe will be masked with an opaque label to ensure that participants are blinded. Unblinded study personnel will be responsible for vaccine preparation and administration. These unblinded personnel will not be involved in study-related assessments or have contact with subjects for data collection following study vaccination.

8.3 Study procedures

8.3.1 General procedures

8.3.1.1 Medical History

The trial clinician will review the medical history of potential study subjects during the screening visit. Particular attention will be paid to:

- Current or recent (within the previous two weeks) acute respiratory illness with or without fever, including symptoms associated with COVID-19;
- Recent receipt of vaccinations or other immune modulator therapy within the previous six months;
- Hypersensitivity of any kind;
- Clinically relevant history of cardiovascular, renal, gastrointestinal, haematological, dermatological, endocrine, neurological or immunological diseases;
- Known or suspected immunologic function impairment of any kind and/or known HIV infection, hepatitis B or hepatitis C infection;
- Mental illness;
- Tobacco, alcohol, or drug use;
- Medication use in the past 6 months;
- For women, pregnancy and contraceptive use and/or history of surgical sterility.

8.3.1.2 Physical examination

A complete physical examination will include the examination of general appearance, skin, neck, eyes, throat, lungs, heart, abdomen, back and extremities, and a routine vascular and neurological examination. Height (cm) and body weight (to the nearest 0.1 kilogram [kg] in indoor clothing, but without shoes) will also be measured, at screening only. Body mass index (BMI) will be calculated using the formula: BMI = Body weight (kg) / [Height (m)]2 and converted to an integer. During follow-up visits a focused physical examination will be performed if deemed necessary by the trial physician.

8.3.1.3 Vital signs

Vital signs including body temperature (degrees Celsius), blood pressure (BP, millimeters of mercury) and pulse measurements (beats per minute) will be recorded at the screening, inclusion and follow up visits. Additional measurements will take place at the discretion of the physician.

Systolic and diastolic BP will be measured while the subject is sitting, with back supported and both feet placed on the floor, using an automated validated device, with an appropriately sized cuff. In case the cuff sizes available are not large enough for the subject's arm circumference, or in case of doubt of accuracy of the automated device a sphygmomanometer with an appropriately sized cuff may be used.

If vital signs are out-of-range at screening or inclusion, the investigator may obtain two additional readings, so that a total of up to three consecutive assessments are made, with the subject seated quietly for approximately five minutes preceding each repeat assessment. At least the last reading must be within the normal range in order for the subject to qualify.

Temperature, blood pressure and pulse are measured as part of the physical examination.

8.3.1.4 Blood sampling and safety laboratory evaluations

During the study, blood samples will be drawn for safety and research purposes. The blood sampling schedule in the flowchart (section 8.3.2.15) shows the maximum amounts of blood that will be drawn. Following universal precautions, blood will be collected by venipuncture into vacutainer tubes. Blood specimens will be affixed with coded labels that link the specimen to the subject, specimen type, specimen collection date, and time-point. The cumulative blood draw for each subject over the entire course of study participation is maximally 230mL.

Biological safety parameters will be measured at the hospital laboratory. In the case where a laboratory assessment is outside the reference range, a decision regarding whether the result is of clinical significance or not shall be made by the investigator and shall be based, in part, upon the nature and degree of the observed abnormality. The assessment may first be repeated for confirmation. All abnormalities will be documented in the source documents, including clinical considerations.

Hemoglobin, hematocrit, red blood cell count, white blood cell count with differential (e.g. neutrophils, basophils, eosinophils, monocytes, lymphocytes) and platelet count; alkaline phosphatase, total bilirubin, gamma-glutamyltransferase (γGT), lactate dehydrogenase (LDH),

alanine aminotransferase (ALT), aspartate aminotransferase (AST), sodium, potassium, creatinine and urea will be measured at regular time points during the study. Glucose, triglycerides and cholesterol will be measured only at screening.

8.3.1.5 Pregnancy Test

A serum pregnancy or midstream urine sample will be obtained and assessed by commercially available hCG urine tests. This test will be done at screening and inclusion and before each vaccine dose administration.

8.3.1.6 Analysis of transmission reducing activity

Membrane-feeding assays demonstrate biologic activity of transmission-blocking antibody and are critical to selection of vaccine candidates. Samples for SMFA will be collected from all subjects. Sera and/or purified IgG prepared from each serum will be used in the SMFA.),
In a SMFA, test serum obtained from immunized subjects is mixed with parasites from a laboratory culture and the mixture is placed in a feeding cup covered with an artificial membrane. Pre-starved mosquitoes from a laboratory colony are allowed to feed through the membrane. A similar procedure is carried out on a malaria-naïve control serum at the same time using mosquitoes raised from the same laboratory colony. One week after the feed, mosquitoes are dissected, and midguts are stained with mercurochrome for the oocyst form of the parasite. The reduction of the proportion of oocyst-laden mosquitoes or the reduction of average oocyst numbers per mosquito compared to mosquitoes fed on the control group demonstrate biologic function of the antibody, and may be predictive of efficacy in the field. SMFA results have been shown to correlate with ELISA antibody titers against Pfs25 in several species. The SMFAs will be conducted at LMIV and Laboratory of Malaria and Vector Research (LMVR) in Rockville, Maryland.

8.3.1.7 Immunological assays: ELISAs

Blood samples will be taken to evaluate the immune response during and after R0.6C and ProC6C vaccinations against the 6C antigen. An ELISA assay will be used to measure circulating anti-6C antibody titres in the subjects' sera at defined time points as shown in section 8.3.2.15, (sub)class and functionality (e.g. complement binding) may also be determined. Exploratory objectives will measure circulating antibody titers via ELISA against the immunogen (e.g. R0.6C or ProC6C) as well as constituent antigens (R0, Pfs48/45-6C, Pfs230-Pro, CSP).

8.3.1.8 Case report forms and data collection

All data collected by the investigators is registered in case report forms. The investigator's notes are collected per subject in the subject file and are considered source data. Since all subjects will be healthy, there is no personal medical file for the study subjects, with exception of the medical file in case of adverse events/reactions resulting in a medical consultation or hospitalization. In this case the medical file will also be considered as source data. The diaries produced by the field workers (nurses) are also considered source data and will be kept as source documents.

8.3.1.9 COVID-19 related measures

With regards to the COVID-19 pandemic, the following measures will be followed. During the study period subjects will be instructed to call the trial physicians at any time if they experience severe symptoms or symptoms possibly related to COVID-19. SARS-CoV2 testing will be performed in accordance with national guidelines. Follow-up visits not requiring blood collection for safety endpoints may be carried out at home, instead of at the clinical research center for subjects with suspected or confirmed SARS-CoV2 infection, until such time as they are considered no longer (potentially) contagious.

8.3.2 Specific procedures

8.3.2.1 Screening visit (V1 D-28-0)

Participants who have consented will undergo the full screening procedure. The purpose of the screening visit is to provide and clarify study information to the volunteer and to answer any questions the subjects may have, prior to obtaining informed consent and determining whether interested subjects are eligible for participation.

Upon arrival for the screening visit and prior to any screening activities, study staff will review the informed consent process with the volunteer. The possibility of withdrawal from the study, at any time and without any declaration of the reason, will be explained to the subjects.

The investigator, or a person designated by the investigator, will fully inform the volunteer of all pertinent aspects of the study and individual consent will be documented by a signature. Subjects may only participate in screening if they have signed the informed consent form. Subjects who sign the informed consent will undergo screening. If certain elements of the screening procedures already indicate that a subject is ineligible for inclusion, screening procedures do not necessarily all have to be completed. The below activities will occur during the screening:

- Patient history: The subject will be further interviewed to collect demographic data, medical history including details of any chronic or recurrent medical and psychiatric conditions and use of adequate contraception;
- A physical examination including vital signs, height and weight will be performed;
- A urine specimen will be collected for a pregnancy test if the females subject is of childbearing potential;
- Blood specimens will be collected for routine clinical laboratory testing of biochemical and hematological parameters, as well as hepatitis B and hepatitis C serological screening

All subjects will be asked to supply a phone number of a partner or roommate who may be contacted in case of emergency. Concomitant medications is recorded at all study visits.

All results of the screening will be reviewed with the subject. Subjects are informed in person or by phone if they have satisfied all the inclusion criteria. If clinically significant abnormalities are identified during screening, subjects will be referred to their primary health provider or appropriate hospital. If identified during the study, subjects may be asked to

return to the study site for further evaluation, including clinical evaluation and repeat laboratory testing as warranted.

If clinically significant (laboratory) abnormalities are identified during the study, subjects may be asked to return to the study site for further evaluation, including clinical evaluation and repeat laboratory testing as warranted. The trial physician can decide to initiate any additional diagnostics (including safety laboratory evaluations) at all times. For unexpected laboratory abnormalities, the laboratory test will be repeated.

All significant abnormalities should be followed up until resolution or stabilization.

8.3.2.2 Inclusion/First vaccination visit (V2, D0)

Subjects meeting the eligibility criteria during screening (section 6.3 and 6.4) will be invited back for enrolment into the study at the inclusion visit. Baseline assessments will be taken on the inclusion day. For each subject, study start (day 0) will be defined as the day of first study vaccine administration. For subjects that do not show up for the inclusion visit, or subjects that do not continue to meet the eligibility criteria, alternate subjects may take their place. At study inclusion, the following activities will occur:

- Patient history will be repeated. Only subjects who still meet the inclusion criteria will be included to receive vaccination;
- Physical exam will be performed
- Blood specimens will be collected for routine clinical laboratory testing of biochemical, hematological parameters, SMFA, immunology (Elisa), and host genetics, blood smear & filter paper;
- A serum sample will be collected for a pregnancy test if the subject females is of childbearing potential;
- Subject will be informed that nurse will visit him/her during 7 days to record any local and systemic symptoms and medication use in diaries
- All subjects will be issued an emergency notification card that details their participation in the study and provides contact phone numbers of the investigators.
- Administration of vaccine
- Subjects will be observed for at least 1 hour after administration. A list of solicited local and systemic symptoms will be reviewed after this observation period. If the occurrence of an adverse event or use of medication is confirmed by the study physician, it is recorded in the CRF.
- After leaving the hospital, a nurse will visit the subject at home to record any adverse events for six days. Any AEs will be recorded in the diary card.

8.3.2.3 Second vaccination (V4, Day 28) and Third vaccination (V6, Day 56)

During these visits, the following will be performed:

- Medical history since the previous visit
- AE review
- Prior and concomitant therapy review
- Physical examination
- Contraindication review

- Serum/urine sample for a pregnancy test if the subject females is of childbearing potential;
- Blood specimens for Laboratory safety and serology (ELISA), SMFA, blood smear & filter paper
- Administration of vaccine
- Subject one hour surveillance.
- CRF completion

8.3.2.4 Contact visits post-vaccination : Home Visit (Day 1-6); Home Visit (Day 29-34) and Home Visit (Day 57-61)

Nurse will visit the subject to record local and systemic signs and symptoms in the diary card. The subject diary will be reviewed by the physician at each scheduled study visit at the health facility and used as a base for discussion of possible local and systemic adverse events or medication use.

8.3.2.5 Visits 14 days post each vaccination: Visit 3 (Day 14), Visit 5 (Day 42), and Visit 7 (Day 70)

During these visits, the following will be performed:

- Medical history since the previous visit
- Diary review
- AE review
- Concomitant therapy review
- Physical examination
- Blood sampling for laboratory safety serology (ELISA), SMFA, blood smear & filter paper
- The CRF will be updated

8.3.2.6 Visit 8 (Day 84,) 28 days post third-vaccination

During this visit, the following will be performed:

- Medical history since the previous visit
- AE review
- Concomitant therapy review
- Physical examination
- Blood sampling for laboratory safety blood smear & filter paper
- Antimalarial treatment to clear existing parasites
- The CRF will be updated

8.3.2.7 Visit 9 (Day 98)

During this visit, the following will be performed:

- Medical history since the previous visit
- AE review
- Concomitant therapy review
- Physical examination
- Blood sampling for blood smear & filter paper
- The CRF will be updated

8.3.2.8 Visit 10 (Day 112)

During this visit, the following will be performed:

- Medical history since the previous visit
- AE review
- Concomitant therapy review
- Physical examination
- Blood sampling for laboratory safety, blood smear & filter paper
- The CRF will be updated

.

8.3.2.9 Visit 11 (Day 126)

During this visit, the following will be performed:

- Medical history since the previous visit
- AE review
- Concomitant therapy review
- Physical examination
- Blood sampling for blood smear & filter paper
- The CRF will be updated

8.3.2.10 Visit 12 (Day 140)

During this visit, the following will be performed:

- Medical history since the previous visit
- AE review
- Concomitant therapy review
- Physical examination
- Blood sampling for laboratory safety, Serology, SMFA, blood smear & filter paper
- The CRF will be updated

8.3.2.11 Visit 13 (Day 154)

During this visit, the following will be performed:

- Medical history since the previous visit
- AE review
- Concomitant therapy review
- Physical examination
- Blood sampling for blood smear & filter paper
- The CRF will be updated

8.3.2.12 Visit 13 (Day 168)

During this visit, the following will be performed:

- Medical history since the previous visit
- AE review
- Concomitant therapy review
- Physical examination
- Blood sampling for blood smear & filter paper
- The CRF will be updated

8.3.2.13 Visit 12 (Day 180,) 4 months post third-vaccination

During the visit, the following will be performed:

- Medical history since the previous visit
- Concomitant therapy review
- Physical examination
- Blood sampling for: Immunology (ELISA), SMFA, Laboratory safety, blood smear & filter paper
- The CRF will be updated

8.3.2.14 Unscheduled visit

Subjects may need to present to the study centre for an unscheduled visit should they experience any AE that requires evaluation by the trial clinician. Data for any examinations performed on the subject at an unscheduled visit must be recorded in the CRF. If an unscheduled visit is performed, the procedures for the next following visit should not be made earlier than scheduled above.

8.3.2.15 Schedule of trial visits and measurements

**Trial Procedures**	**Collection Tube**	**Visit /Home visit**	**1 (Screening)**	**2**	**HV**	**3**	**4**	**HV**	**5**	**6**	**HV**	**7**	**8**	**9**	**10**	**9**	**Unscheduled**
		**Timelines Days**	**D-28-0**	**D0**	**D1-7**	**D14**	**D28**	**D29-35**	**D42**	**D56**	**D57-63**	**D70**	**D84**	**D112**	**D141**	**D180**	
		**Time window Days**		**0**			**±2D**			**±2D**			**±2D**	**±7D**	**±7D**	**±7D**	
Informed consent			●														
Eligibility criteria			●	●													
Demographic data and medical history			●														
Physical examination[1]			●	●	●	●	●	●	●	●	●	●	●	●	●	●	●
Height and weight			●														
Vital signs[2]			●	●	●	●	●	●	●	●	●	●	●	●	●	●	●
Concomitant medications			●	●	●	●	●	●	●	●	●	●	●	●	●	●	●
HBV, HCV (5mL)	SST		●														
Pregnancy test (Urines/Serum)[6]	SST/Urines container		●	●			●			●						●	
Contraindication Review				●			●			●							
**Vaccination/immediate surveillance**				☒			☒			☒							
Solicited local and systemic AEs (days 0–7)				●	●		●	●		●	●						
Unsolicited AEs				●	●	●	●	●	●	●	●	●	●				
SAEs				●	●	●	●	●	●	●	●	●	●	●	●	●	●
Blood smear & filter paper[7]				●		●	●		●	●		●	●	●	●	●	●
Complete blood count[3](2ml)	EDTA		●	●		●	●		●	●		●	●	●	●	●	●[7]
Biochemistry tests[4] (3ml)	SST		●	●		●	●		●	●		●	●	●	●	●	●[7]
Host genetic				●													
Serum samples (serology/ELISA) (10 ml)	SST			●		●	●		●	●		●			●	●	
SMFA[5] (10ml)	SST			●		●	●		●	●		●			●	●	
Daily blood volume (ml)			10	25		25	25		25	25		25	5	5	25	25	
Cumulative blood volume (ml)			10	35		60	85		110	135		160	175	180	205	230	

1. A physical examination including height and weight and vital parameters will be performed at the screening visit. On other visits, measurements may take place at the discretion of the physician.

2. Vital signs including body temperature, blood pressure and pulse will be recorded at the screening and inclusion visit. On other visits, measurements may take place at the discretion of the physician.
3. CBC test includes: hemoglobin, hematocrit, platelets, red blood cell count, MCV, white blood cell count + differentiation
4. Biochemistry test includes: creatinine, urea, sodium, potassium, bilirubin, yGT, AST, ALT and LDH. Additional at screening: cholesterol, triglyceride and glucose.
5. SMFA assays will be performed at 6 time points.
6. The serum can be taken in the 3ml dedicated to biochemistry..
7. Sample will be taken from haematology
8. Serum can be taken in the 3ml dedicated to biochemistry.

8.4 Provision of care to the trial participants

Study contact personnel will be available 24 hours a day at trial site clinic seven days a week, to attend consulting participants. Participants requiring inpatient care will be admitted to the hospital where study personnel will be posted. Laboratory and radiological investigations will be carried out when appropriate. If necessary, participants requiring more specialized care (treatment or diagnostic procedures) will be transported to a referral hospital. Treatment for medical conditions will be given according to the standard treatment regimens locally. Any expenses including transport incurred by the participants themselves for clinical care related to acute conditions will be borne by the trial according to the appropriate local arrangements. Long-term care for chronic conditions unrelated to study procedures will be delivered following local guidelines with no financial support from the trial.

8.4.1 Malaria case management

Clinical or symptomatic malaria for this study is defined as the presence of asexual *P. falciparum* parasites at any parasitemia with at least one of the following symptoms: temperature of ≥37.5°C and/or one or more of the following symptoms: headache, myalgia, arthralgia, malaise, nausea, dizziness, or abdominal pain. Clinical or symptomatic malaria will be reported as an AE. Trial subjects with uncomplicated or severe malaria will be treated according to SOPs and national guidelines.

8.4.2 Pregnancy management

Trial participants who become pregnant during the trial period after the first vaccination will not receive any subsequent vaccination but will not be withdrawn from the trial and will continue to be followed up. Female participants will be instructed to notify the investigators if they become pregnant at any time during the entire trial period. The investigator will collect pregnancy information on any woman participant, who becomes pregnant while participating in this study. The investigator will record pregnancy information on the pregnancy report form and submit it to the sponsor's representative within 24 hours after learning of a subject's pregnancy. The volunteer will also be followed to determine the outcome of the pregnancy, (whether full term or prematurely terminated). Information on the status of the mother and child will be forwarded to sponsor or its designee.

8.5 Blinding and code Breaking Procedure

The vaccines will be different in terms of volume and appearance. Therefore, the contents of the syringe will be masked with an opaque label to ensure that participants, are blinded. To ensure treatment concealment, allocation to the investigational vaccine or to the control group will be done using sequentially numbered opaque sealed envelopes which an independent pharmacist will prepare and seal, and then provide to the investigator. The trial pharmacist will only be allowed to open an envelope after ensuring that the participant before him has met all eligibility criteria and has been given a study ID number. For each participant, eligibility will

have to be counter checked and signed by a second person before allocation of study ID number. All envelopes will be retained to be checked by the clinical monitor.
Breaking the code will be done in case participant safety is compromised when allocation status is not known. This can be in an emergency situation that is potentially related to vaccine administration or the occurrence of a SAE where information on allocation status is important for proper clinical management.
The LSM who is independent from the study team will therefore be provided with code break envelopes for each study enrolled subject, associating each treatment number with a specific vaccine. These envelopes will be kept in a safe and locked place with no access for unauthorized personnel. A study-specific SOP (Unblinding SOP) that describes unblinding shall be in place before first vaccination.

9. SAFETY REPORTING

9.1 Adverse events (AEs)

Adverse events are defined as any undesirable experience occurring to a subject during the study, whether or not considered related to the investigational product. All adverse events reported spontaneously by the subject or observed by the investigator or his staff will be recorded. Abnormal laboratory findings (e.g. clinical chemistry or hematology) or other abnormal assessments that are judged by the investigator to be clinically significant will be recorded as AEs (or SAEs if they meet the definition). The investigator will exercise his or her medical and scientific judgment in deciding whether an abnormal laboratory finding, or any other abnormal assessment is clinically significant. If there are any severe complaints, the volunteer will be evaluated immediately by a qualified clinician using the appropriate clinical assessments according to standard hospital care.

9.1.1 Adverse event data collection and recording

Adverse events will be collected at all follow-up visits, using the memory aid booklets as a basis, and whenever a subject reports signs or symptoms to the trial physician between visits.
After administration of each dose of vaccine, the following systemic signs and symptoms will be solicited until 7 days after administration:
Fever, headache, myalgia, fatigue, chills, nausea/vomiting, diarrhea, abdominal pain, myalgia, arthralgia, urticaria and rash. The time points that these symptoms are solicited are indicated in section 8.3.2.15.
After the administration of each dose of vaccine, the following local signs and symptoms will be solicited until 7 days after administration:
Pain/tenderness, itching, swelling, induration, limitation of arm movement and erythema/redness at injection site. The time points that these symptoms are solicited are indicated in section 8.3.2.15.

If known, the trial clinician will record the diagnosis (i.e., disease or syndrome) rather than component signs, symptoms and laboratory values. If the signs and symptoms are considered unrelated to an encountered syndrome or disease they should be recorded as individual AEs. If a primary AE is recorded, events occurring secondary to the primary event should be described

in the narrative description of the case (e.g. primary AE = Orthostatic hypotension; secondary event may be fainting, head trauma, etc.). In case of hospitalizations for surgical or diagnostic procedures, the pre-existing condition should be recorded as the SAE, not the procedure itself. All adverse events/reactions (solicited and unsolicited) will be accurately documented in the case report form by the investigators. For each event/reaction the following details will be recorded:

1. Description of the event/reactions (MedDRA terminology will be included in the CRF)
2. Duration (date and time of occurrence and end date and time)
3. Intensity (see explanation directly below)
4. Relationship with the intervention (see section 9.1.2)
5. Action taken, including treatment

Grading for swelling/induration

Grade	**Diameter [mm]**
0	0
1	< 20
2	20 – 50
3	> 50

Grading for erythema/redness

Grade	**Diameter [mm]**
0	0
1	< 50
2	50 – 100
3	> 100

The presence and severity of local pain/tenderness, itching, swelling, limitation of arm movement at the site of vaccination will be determined using the following scale:

**Grade**	**Description**
**0**	No pain at all
**1**	Painful on touch, no restriction in movement of limb
**2**	Painful when limb is moved
**3**	Unable to use limb due to pain

The intensity of the solicited systemic symptoms will be evaluated as in the table below:

Symptôme	Grade	Description
Fever	0	<37.5ºC
	1	37.5 - ≤38.0ºC
	2	>38.0 - ≤39ºC
	3	>39ºC
Chills	0	None
	1	Chills that are easily tolerated
	2	Chills that interfere with daily activity
	3	Chills that prevent daily activity
Nausea/vomiting	0	None
	1	Nausea/vomiting that is easily tolerated
	2	Nausea/vomiting that interferes with daily activity
	3	Nausea/vomiting that prevents daily activity
Headache	0	None
	1	Headache that is easily tolerated
	2	Headache that interferes with daily activity
	3	Headache that prevents daily activity
Malaise	0	None
	1	Malaise that is easily tolerated
	2	Malaise that interferes with daily activity
	3	Malaise that prevents daily activity
Myalgia	0	None
	1	Myalgia that is easily tolerated
	2	Myalgia that interferes with daily activity
	3	Myalgia that prevents daily activity
Joint pain	0	None
	1	Joint pain that is easily tolerated
	2	Joint pain that interferes with daily activity
	3	Joint pain that prevents daily activity
Fatigue	0	None
	1	Fatigue that is easily tolerated
	2	Fatigue that interferes with daily activity
	3	Fatigue that prevents daily activity
Diarrhea	0	None
	1	diarrhea that is easily tolerated
	2	diarrhea that interferes with daily activity
	3	diarrhea that prevents daily activity
abdominal pain	0	None
	1	abdominal pain that is easily tolerated
	2	abdominal pain that interferes with daily activity
	3	abdominal pain that prevents daily activity

Urticaria	0	None
	1	Urticaria that is easily tolerated
	2	Urticaria that interferes with daily activity
	3	Urticaria that prevents daily activity
Rash	0	None
	1	Rash that is easily tolerated
	2	Rash that interferes with daily activity
	3	Rash that prevents daily activity

The intensity of the unsolicited systemic symptoms will be ranked as (1) mild, (2) moderate, (3) or severe, according to the following scale:

Mild (grade 1): awareness of symptoms that are easily tolerated and do not interfere with usual daily activity

Moderate (grade 2): discomfort that interferes with or limits usual daily activity

Severe (grade 3): disabling, with subsequent inability to perform usual daily activity, resulting in absence or required bed rest

If an adverse event changes in intensity during the specified reporting period, a new description of the adverse event will be added. Interrupted AEs are registered as one AE if the interruption is <24 hours. When an AE/SAE occurs, it is the responsibility of the investigators to review all documentation (e.g. hospital progress notes, laboratory, and diagnostics reports) related to the event. The investigators will then record all relevant information regarding an AE/SAE on the CRF or SAE Report Form, respectively.

9.1.2 Assessment of causality

The investigators should assess the relationship between study procedures and the occurrence of each AE/SAE. The investigators will use clinical judgment to determine the relationship. Alternative causes, such as natural history or the underlying diseases, concomitant therapy, other risk factors and the temporal relationship of the event will be considered and investigated. The relationship of the adverse event with the study procedures will be categorized as:

Definitely: administration of the IP is the cause, another aetiology causing the adverse event is not known.

Probable: administration of the IP is the most likely cause: however, there are alternative reasonable explanations, even though less likely.

Possible: there is a potential association between the event and administration of the IP, however, there is an alternative aetiology that is more likely.

Unlikely: a relationship to the administration of IP is unlikely, however, it cannot be ruled out.

Not related: a relationship to the administration of the IP cannot be reasonably established; another aetiology is known to have caused the adverse event or is highly likely to have caused is.

When a regulatory authority requests a binary classification (related vs. unrelated), definitely, probably and possibly related are considered to be “related”, while not related and unlikely related are considered to be “unrelated”. Thus, an intervention-related AE refers to an AE for which there is a possible, probable or definite relationship to the study intervention. The investigator will use clinical judgment to determine the relationship.

The degree of certainty with which an AE can be attributed to administration of the study vaccine will be determined by how well the event can be understood in terms of one or more of the following:

- The event being temporally related with vaccination or reproduced on re-vaccination.
- A reaction of similar nature having previously been observed with this type of vaccine and/or formulation.
- The event having been reported in the literature for similar types of vaccines.
- Whether or not there is another identifiable cause.

All local (injection site) reactions will be considered causally related to vaccination. All malaria cases will be reported as not related to vaccination.

Causality assessment will be provided by the Principal Investigator or designee and reviewed by the sponsor. The sponsor may make a separate and final determination on the “reasonable possibility” that the event was “related” or “unrelated” to the study agent, in keeping with applicable (US FDA) guidance on sponsor IND safety reporting.

9.1.3 Treatment of adverse events

Treatment of any adverse event will be provided by the investigators. The applied measures will be recorded in the CRF of the participant. The recording of adverse events is an important aspect of study documentation. It is the responsibility of the investigators to document all adverse events according to the detailed guidelines set out. The participants will be instructed to contact the investigators immediately if they manifest any signs or symptoms they perceive as serious.

9.1.4 Serious adverse events (SAEs)

A serious adverse event is any untoward medical occurrence or effect that:

- results in death;
- is life threatening (at the time of the event)*;
- requires hospitalisation or prolongation of existing inpatients’ hospitalisation;
- results in persistent or significant disability or incapacity;

- is a congenital anomaly or birth defect; or
- Important medical events that may not result in death, be life-threatening, or require hospitalizations may be considered serious when, based upon appropriate medical judgment they may jeopardize the patient or subject and may require medical or surgical intervention to prevent one of the outcomes listed in this definition. Examples of such medical events include allergic bronchospasm requiring intensive treatment in an emergency room or at home, blood dyscrasias or convulsions that do not result in inpatient hospitalization, or the development of drug dependency or drug abuse.

An elective hospital admission will not be considered as a serious adverse event.

* Life-threatening AE. An AE is considered “life-threatening” if, in the view of either the investigator or sponsor, its occurrence places the patient or subject at immediate risk of death. It does not include an AE, had it occurred in a more severe form, might have caused death.

The investigator will report all SAEs to the sponsor without undue delay after obtaining knowledge of the events (within 24 hours).

9.1.5 Suspected unexpected serious adverse reactions (SUSARs)

Adverse reactions are all untoward and unintended responses to an investigational product related to any dose administered. Unexpected adverse reactions are SUSARs if the following three conditions are met:

1. the event must be serious (see chapter 9.1.4);
2. there must be a certain degree of probability that the event is a harmful and an undesirable reaction to the medicinal product under investigation, regardless of the administered dose;
3. the adverse reaction must be unexpected, that is to say, the nature and severity of the adverse reaction are not in agreement with the product information as recorded in:
   - Summary of Product Characteristics (SPC) for an authorised medicinal product;
   - Investigator’s Brochure for an unauthorised medicinal product.

The sponsor will report expedited all SUSARs to the competent authorities, according to the requirements. The expedited reporting will occur not later than 15 days after the sponsor has first knowledge of the adverse reactions. For fatal or life threatening cases the term will be maximal 7 days for a preliminary report with another 8 days for completion of the report..

9.2 Annual safety report

In addition to the expedited reporting of SUSARs, the sponsor will submit, once a year throughout the clinical trial, a safety report to the ethical committees and competent authority. This safety report consists of:

- a list of all suspected (unexpected or expected) serious adverse reactions, along with an aggregated summary table of all reported serious adverse reactions, ordered by organ system, per study;

– a report concerning the safety of the subjects, consisting of a complete safety analysis and an evaluation of the balance between the efficacy and the harmfulness of the medicine under investigation.

9.3 Follow-up of adverse events

All AEs will be followed until they have abated, or until a stable situation has been reached. Depending on the event, follow up may require additional tests or medical procedures as indicated, and/or referral to the general physician or a medical specialist.
SAEs need to be reported till end of study, as defined in the protocol. After termination of the trial, the investigator should assure that the subject is referred for medical follow-up, as appropriate.

9.4 Data Safety Monitoring Board (DSMB)

An independent DSMB composed of at least three independent individuals will be appointed. The DSMB will include a local safety monitor, and two experts nominated by the PI. The DSMB will be established for the purpose of monitoring the study and to provide independent, non-binding advice on safety and ethics. The responsibilities and procedures of the DSMB members are defined in the DSMB Charter.
The advice(s) of the DSMB will only be sent to the sponsor of the study.

9.4.1 Local safety monitor (LSM)

For this study, a local safety monitor will be appointed, who will be involved in the review of severe and serious adverse events and volunteer safety. He/she is independent of the sponsor and the investigator. The local safety monitor is notified of all grade 3 adverse events probably or definitely related to R0.6C administration and persisting at grade 3 for >48 hours.

9.4.2 Safety Meetings

The DSMB will review safety data at pre-defined time points throughout the study, specifically: upon completion of ≥7 days of post-administration follow-up in all subjects in a given dose-group and prior to commencing the first administration in the subsequent dose group. The chair of the DSMB will determine for each time point whether a meeting will be held, or whether a recommendation from all members may be formalized through e-mail. The frequency of these reviews may be adapted upon DSMB recommendation if deemed necessary. In addition, safety data for all participants will be assessed by the DSMB at the end of the study. An ad hoc DSMB meeting may be convened at any time or at the request of the PI or local safety monitor to review safety data from subjects and/or groups who meet any of the holding rules as specified in the protocol (9.5.4) and DSMB charter.

9.4.3 Safety Reports

Safety reports will be prepared by the clinical investigator for review by the committee prior to each review. These reports will provide at a minimum the following information:

- Accrual data and subject status data with regard to completion of/discontinuation from the study.

- Summaries of solicited AEs, classified by severity.
- Unsolicited AEs (including SAEs), categorized by MedDRA coding, severity and relatedness to study vaccine.
- Safety laboratory test results outside of normal institution reference ranges and considered clinically significant, classified by severity grading scale (irrespective of whether assessed as AEs).
- Any new or updated AEs that have met the holding rules.

The DSMB will review the safety data within 2 working days. The DSMB will summarize their recommendations to the study Sponsor as to whether there are safety concerns and whether the study should continue without change, be modified, or be terminated. If at any time a decision is made to permanently discontinue administration of study vaccines administrations in all subjects, the Sponsor will notify the competent authorities.

9.4.4 Dose progression

Progression to the high dose cohort will depend in all cases upon a positive review by the DSMB of cumulative safety data up to 7 days post vaccination of the second dose of the lower dose cohort. The data to be reviewed before each dose group includes the safety data (solicited and unsolicited AEs through day 7 post administration) and clinically significant laboratory tests collected at 0 and 7 days following administration for all subjects in the specified dose group. The DSMB will review the AEs (local, general, and laboratory) and will make recommendations regarding the initiation of the high dose cohort.

9.4.5 Safety holding rules

The study may be placed on safety hold at any time for the following reasons:

- On advice of the safety monitor;
- On advice of the Principal/Clinical investigators;
- On advice of the DSMB;
- If holding rules are met (see below).
  - Any SAE (Serious Adverse Event) possibly, probably or definitely related to vaccination
  - 50% or more of subjects having a Grade 3 adverse reaction persisting at Grade 3 for > 48 hours within 7 days following each vaccine dose (day of vaccination and 7 subsequent days). The threshold of 50% corresponds to a consensus decision in accordance with the African Malaria Network Trust (AMANET) Malaria vaccine guidelines and will be applied for the start of the vaccination of the high dose cohort.

If any vaccinee experiences an SAE related to vaccination, then all the immunisations of the subjects in this cohort will be stopped. The vaccination will only be resumed upon the decision of the sponsor according to the recommendation of the DSMB.

The study site member first aware of the event meeting the holding rule will notify the Principal Investigator and the Local Safety Monitor. The PI will alert the appropriate parties. The DSMB

will be notified within 24 hours. An ad hoc DSMB review will be performed. The following considerations must be discussed:

- Relationship of the AE or SAE to the study product
- Relationship of the AE or SAE to Malaria vaccines dose
- Relationship of the AE or SAE to (one of) the adjuvants
- If appropriate, additional screening or laboratory testing is provided to other subjects to identify subjects who may develop similar symptoms
- If any study related SAE is not listed on the current informed consent form (ICF), the PI will revise the ICF and subjects will be asked to provide consent on the new ICF.

10. TRIAL ENDPOINTS

10.1 Primary endpoints:

1) Incidence of serious adverse events and solicited grade 3 local and systemic adverse events (AEs) possibly, probably or definitely related to the vaccine in the period from first vaccination up to 1 month after the last immunization.

10.2 Secondary endpoints:

1) The TRA at other timepoints (2 weeks after the first, second and second immunizations) and 4 months post third vaccination compared to baseline (D0) in each of the three dose-adjuvant groups.
2) The anti-6C antibody quantity in volunteer sera collected two weeks after each dose and 4 months post dose 3 compared to baseline in each of the three dose-adjuvant combinations, as determined by ELISA.

10.3 Exploratory Endpoints:

1) The antibody decay rate following immunization (R0.6C or ProC6C) for each of the three dose-adjuvant combinations against the immunogens or constituent antigens.
2) The difference in peak anti-6C antibody quantity between the two dose groups (30 or 100µg R0.6C and 30 or 100µg ProC6C).
3) Time to *P. falciparum* malaria infection by PCR
4) Incidence of clinical malaria episodes defined as fever (temperature ≥38.0 by any route and/or History of fever within the last 48 hours & positive malaria smear (>0, 500, 2500, ≥5,000)

11. DATA MANAGEMENT

11.1 Data Management

The Principal Investigator will be responsible for receiving, entering, cleaning, querying, analysing and storing all data that accrues from the trial. Responsibility for this may be delegated to the data manager. The data will be entered into the subjects' CRFs. Data will be subsequently transferred to an electronic database for analysis. If any changes to the protocol are necessary during the study a formal amendment will be presented to the sponsor's representative prior to submission to the relevant ethical and regulatory agencies for approval unless to eliminate an immediate hazard(s) to study participants in which case no prior ethics approval is needed. Any unforeseen and unavoidable deviations from the protocol will be documented and filed as a protocol deviation in the trial file, with explanation.

11.2 Data Capture Methods

Data capture will be on paper CRFs. The CRFs will be considered source documents as healthy volunteers will not have hospital case notes. Adverse events will be tabulated in an electronic database for descriptive analysis. Immunological data will be transferred to an electronic database for analysis without any volunteer identifier apart from the unique volunteer number.

12. STATISTICAL ANALYSIS

All data will be collected and verified prior to analysis. All data will be analysed in accordance with the Statistical Analysis Plan. Final analyses of all data will occur after study completion and final verification of data according to GCP. All data analyses will be conducted using IBM SPSS, R, SAS or Graphpad using the latest version available. Detailed statistical procedures, listings, table shells, and figures will be provided in a SAP prior to analysis. The SAP will be finalized before study close-out and database lock. The following key statistical components will be considered and a detailed description will be documented in the SAP:

- Primary and secondary endpoints and how they will be measured
- Statistical methods and tests that will be used to analyze the endpoints
- Strategy that will be used if the statistical test assumptions are not satisfied
- Indication of whether the comparisons will be using one-tailed or two-tailed *t*-test (with justification of the choice) and the level of significance to be used
- Identification of whether any adjustments to the significance level or the overall P-value will be made to account for any planned or unplanned subgroup analyses or multiple testing
- Specification of potential adjusted analyses and a statement with which covariates or factors will be included
- Planned exploratory analyses and justification of their importance
- Any subgroup effects with biological justification and support from within and outside the study

12.1 Primary study parameter

The number of subjects enrolled, completed, or withdrawn will be summarized. Reasons for withdrawal, when known, will be provided. Demographic data will be summarized by descriptive statistics and will include total number of observations (n), mean, standard deviation (SD) and range for continuous variables, and number and percentages for dichotomous variables. The primary safety, and reactogenicity outcomes will include all subjects who meet the eligibility criteria, received the study product, and for whom safety data are available.

<u>Safety Analyses</u>

For the safety analysis, data from all subjects who received at least one dose and for whom safety data are available will be included. All analyses will be descriptive. Data will be presented by dose, overall/dose and overall/subject. Results will be summarized by study group. The percentage of subjects with at least one local AE (solicited or unsolicited), with at least one general AE (solicited or unsolicited) and with any AE during the solicited follow-up period will be tabulated with exact 95% CI (two-sided). No multiplicity adjustment will be implemented in analysis. The same calculations will be performed for AEs rated as grade 3. The percentage of subjects reporting each individual solicited local and general AE during the solicited follow-up period will be tabulated with exact 95% CI. The same tabulation will be performed for grade 3 AEs and for AEs with relationship to vaccine administration. The reports of unsolicited AEs

will be reviewed by a physician and will be categorized by MedDRA terms. The percentage of subjects with at least one report of unsolicited AE and reported up to 28 days after vaccine administration will be tabulated with exact 95% CI. The same tabulation will be performed for grade 3 unsolicited AEs and for unsolicited AEs with a relationship to vaccine administration. SAEs will be described in detail. Withdrawals due to AEs/SAEs will also be summarized. Vital signs which are outside of the normal range and clinically significant will also be listed in tables. The frequency of signs and symptoms will be compared between groups with the chi- square test or fishers exact test. Serious adverse events (SAEs) occurring at any point during the trial will be summarized and relatedness to vaccine will be assessed.

Clinical Laboratory Data Analyses

Any clinically important deviations in routine laboratory test results and/or vital signs as determined by the investigator will be listed. Isolated laboratory abnormalities will be reported as AEs if they are considered clinically relevant by the investigator. Vital signs which are considered clinically relevant by the investigator will be summarized. All adverse events will be listed by participant and will include details of onset time, duration, severity and relationship to the study product.

12.2 Secondary study parameter(s)

SMFA will be performed to determine TRA of R0.6C and ProC6C. Differences will be assessed by comparing mean values between time points using either a two-tailed student's t-test or a non-parametric equivalent. Paired tests will be used if pre-intervention values are compared with post-intervention values. For discrete variables (e.g. the number of positive assays), the chi-squared test or Fisher's exact test will be used (two-tailed).

13. ETHICAL CONSIDERATIONS

13.1 Regulation statement

This study will be conducted in accordance with the latest Fortaleza revision of the Declaration of Helsinki (2013), the Medical research Involving Human Subjects act (WMO), the ICH Good Clinical Practice, and local regulatory requirements. The investigators are responsible for obtaining all relevant ethical and regulatory approvals of the protocol and any subsequent amendments in compliance with local law before the start of the study.

13.2 Recruitment and consent

As soon as the study is approved by the competent authority, healthy subjects will be recruited to participate in the study. Community assent process will start. Community assent will be obtained from the local administrative structures, opinion leaders, family heads, and other community members after explanation and discussion of the study. The community permission process goes through the following steps:

- Study investigators/personnel explain the study to the local administration: mayor, district physician and heads of the peripheral health facilities.

- Study investigators/personnel explain the study to village leaders, including the village chief, opinion leaders, family heads, associations …
- The study and the informed consent process are explained in detail to the potential volunteers by study investigators/personnel.

Informed consent will be obtained using simple French or in local language for not literate subjects in presence of an independent witness who is not a member of the study team.

Subjects who are interested in participating will be invited to come for a screening visit. In the event that the number of volunteers is large in relation to the size of the study sample, a public draw will be made to select the subjects to participate in the screening. Eligible subjects may only be included in the study after providing written, approved informed consent. Informed consent must be obtained before conducting any study-specific procedures (i.e. all of the procedures described in the protocol, including screening procedures). The process of obtaining informed consent should be documented in the subject source documents. During the screening visit, the inclusion and exclusion criteria will be checked. Again, the investigator will answer any questions the volunteer has. The possibility of withdrawal from the study, at any time, without penalty and without any declaration of the reason will be pointed out to the subjects. The investigators will be responsible for providing adequate verbal and written information regarding the objectives and procedures of the study, the potential risks involved and the obligations of the subjects. Subjects will be informed that they will not gain health benefits from this study. Trainees or other students who might be dependent on the investigators or the study group will not be included in the study.

Age of consent for married or otherwise emancipated minors is 20 years and above in Burkina Faso. Thus, emancipated minors will sign a consent and will not need parent/guardian to also sign the consent. The study consent form will be translated orally into local languages and dialects in the event that a potential study participant does not read or speak French (which will likely be a majority of the potential participants). Verification that the oral translations are accurate and that the contents of the informed consent form were transmitted to the participant orally will be done by an independent witness.

13.3 Benefits and risks assessment

Testing in human subjects remains the only reliable and convincing way to obtain information on the immunological responses that are important for protection against malaria. Explorative studies looking for new and complementary candidate malaria interventions are of paramount importance with the potential of large-scale application in endemic countries. Of course, the compelling need for new methods of malaria interventions needs to be balanced with the potential risks and discomforts for the subjects. Risks for subjects are related to administration with R0.6C or ProC6C TBV candidate. There are no direct benefits to participation in the trial for subjects.

13.4 Compensation for injury

The trial will be covered by clinical trials insurance.

13.5 Incentives

Participants enrolled in the trial will be offered compensation for transport expenses.
Participants will also receive monetary compensation for the daily loss of earnings due to the participation to the trial.

14. ADMINISTRATIVE ASPECTS, MONITORING AND PUBLICATION

14.1 Handling and storage of data and documents

A data management plan will be developed prior to start of study describing data management activities from project set-up through data lock and transfer. Designated trial staff will enter the data required by the protocol into the CRF. All data should be recorded, handled and stored in a way that allows its accurate reporting, interpretation and verification. An external monitor will review the data entered into the CRFs by investigational staff for completeness and accuracy and will instruct the site personnel to make any required corrections or additions. Queries are made during each monitoring visit. Designated investigator site staff are required to respond to the queries and confirm or correct the data.

14.2 Monitoring and Quality Assurance

Before study initiation, the protocol and CRFs together with relevant SOPs will be reviewed by the sponsor, the investigators and their staff. During and after completion of the study, the data monitor will visit the site to check the completeness of records, the accuracy of entries on the CRFs, the adherence to the protocol and to Good Clinical Practice and the progress of enrolment.

The investigator will maintain source documents for each subject in the study, consisting of case and visit notes containing demographic and medical information, laboratory data, subject’s diaries, and the results of any other tests or assessments. All information on CRFs must be traceable to these source documents in the subject's file. As with all parts of the CRF, there is an audit trail in place to register every data entry. The investigator will also keep the original informed consent form signed by the subject (a signed copy is given to the subject).

The investigator will give the external monitor access to all relevant source documents to confirm their consistency with the CRF entries. According to the NFU risk classification system, this clinical trial has been classified as high risk. The monitor will perform full verification for the presence of informed consent, adherence to the inclusion/exclusion criteria and documentation of SAEs. The recording of data that will be used for all primary and safety variables will be assessed for 100% of included subjects.

<u>External monitoring</u>

To ensure that the study is conducted in accordance with ICH-GCP and regulatory requirements, monitoring responsibilities will be provided by CRO. A site initiation visit will be conducted prior to beginning of the study, and monitoring will be conducted at initiation, during, and at closeout of the study. During the course of the study, monitors will verify compliance to the protocol; completeness, accuracy, and consistency of the data and study product accountability; and adherence to ICH-GCP and applicable regulations. As needed and when appropriate, the monitors will also provide clarifications and additional training to help the site resolve issues identified during the monitoring visit. As appropriate and informed by risk assessment, remote centralized monitoring activities may be considered

in place of or to supplement onsite monitoring. These may include analysis of data quality (e.g., missing or inconsistent data), identification of data trends not easily detected by onsite monitoring, and performance metrics (e.g., screening or withdrawal rates, eligibility violations, timeliness, and accuracy of data submission).
The extent and frequencies of the monitoring visits will be described in a separate monitoring plan developed prior to study initiation. The investigator will be notified in advance of scheduled monitoring visits. The monitors should have access to all trial related sites, subject medical records, study product accountability, and other study-related records needed to conduct monitoring activities. CRO will share the findings of the monitoring visit, including any corrective actions, with the site investigator. The site PI and the monitors must cooperate to ensure that any problems detected in the course of these monitoring visits are resolved in a predefined timeframe.
To ensure the quality of clinical data for all subjects, a clinical data management review will be performed on subject data received by CRO. During this review, subject data will be checked for consistency, omissions, and any apparent discrepancies. In addition, the data will be reviewed for adherence to the protocol and ICH-GCP. To resolve any questions arising from the clinical data management review process, data queries and/or site notifications will be sent to the site for resolution as soon as possible and within the period described in the monitoring plan; all queries must be resolved prior to database lock.
Essential documents must be filed in the site study file on an ongoing basis and be available for review by CRO.

14.3 Amendments

Amendments are changes made to the research after a favourable opinion by the competent authorities has been given. All amendments will be notified to the competent authorities that gave a favourable opinion.

A ‘substantial amendment’ is defined as an amendment to the terms of the competent authorities application, or to the protocol or any other supporting documentation, that is likely to affect to a significant degree:

- the safety or physical or mental integrity of the subjects of the trial;
- the scientific value of the trial;
- the conduct or management of the trial; or
- the quality or safety of any intervention used in the trial.

All substantial amendments will be notified to the competent authorities and to the competent authority.
Non-substantial amendments will not be notified to the competent authorities and the competent authority, but will be recorded and filed by the sponsor.

14.4 Annual progress report

The sponsor/investigator will submit a summary of the progress of the trial to the competent authorities once a year. Information will be provided on the date of inclusion of the first subject,

numbers of subjects included and numbers of subjects that have completed the trial, serious adverse events/ serious adverse reactions, other problems, and amendments.

14.5 Temporary halt and (prematurely) end of study report

The sponsor will notify the competent authorities and the competent authority of the end of the study within a period of 90 days. The end of the study is defined as the last patient's last visit. The sponsor will notify the competent authorities immediately of a temporary halt of the study, including the reason of such an action. In case the study is ended prematurely, the sponsor will notify the competent authorities and the competent authority within 15 days, including the reasons for the premature termination. Within one year after the end of the study, the investigator/sponsor will submit a final study report with the results of the study, including any publications/abstracts of the study, to the competent authorities and the Competent Authority.

14.6 Public disclosure and publication policy

The study will be registered in Clinicaltrials.gov registry once approved by the competent authorities.

The results of this study will be published in peer-reviewed open access journals and presented at scientific meetings. Sponsor-initiated publications and/or presentations will be agreed upon between the Investigator and Sponsor. Investigator-initiated publications and/or presentations will be provided for review by the Sponsor at least 30 days before the submission deadline for the manuscript and/or presentation abstract to enable relevant input based on information from other studies that may not yet be available to the Investigator.

Any formal publication of the study in which input of the Sponsor personnel exceeded that of conventional monitoring will be considered as a joint publication by the Investigator and the appropriate Sponsor personnel. Authorship will be determined by mutual agreement in accordance with International Committee of Medical Journal Editors recommendations. Additional authors will be agreed prior to journal submission.

14.7 Timeframe and duration of the study

14.7.1 Timeline

The total duration of the project is projected to take approximately 12 months, including approximately 8 months of patient recruitment and follow up.

14.7.2 Gantt chart

	2020	2021				2022			
	Q4	Q1	Q2	Q3	Q4	Q1	Q2	Q3	Q4
**Activity**									
Protocol development	■								
IRB approval / Annual Review		■	■	■					
Regulatory approval/ Annual Review			■	■	■				
CRF and SOP development	■	■	■	■					
Investigational product shipment				■					
Site Initiation visit				■					
Participants recruitment & Follow up					■	■	■		.
Study closeout								■	
Immunological assays					■	■	■	■	
Manuscript and results dissemination									■

	2020	2021				2022				2023	
	Q4	Q1	Q2	Q3	Q4	Q1	Q2	Q3	Q4	Q1	Q2
**Activity**											
Protocol development	■										
IRB approval / Annual Review		■	■	■							
Regulatory approval/ Annual Review			■	■	■						
CRF and SOP development	■	■	■	■							
Investigational product shipment				■							
Site Initiation visit				■							
Participants recruitment & Follow up						■	■	■	.		
Study closeout									■		
Immunological assays						■	■	■	■	■	■
Manuscript and results dissemination										■	■

Appendix A. Toxicity Tables

Vital Sign AE Grading[1]

Vital Signs[2]	Mild (Grade 1)	Moderate (Grade 2)	Severe (Grade 3)	Potentially Life Threatening (Grade 4)
Tachycardia - beats per minute; at rest + calm	101 – 115	116 – 130	> 130	ER visit or hospitalization for arrhythmia
Bradycardia - beats per minute[3]; at rest + calm	50 – 54	45 – 49	< 45	ER visit or hospitalization for arrhythmia
Hypertension (systolic) -mm Hg; at rest + calm	141 – 150	151 – 155	> 155	ER visit or hospitalization for malignant hypertension
Hypertension (diastolic) - mm Hg; at rest + calm	91 – 95	96 – 100	> 100	ER visit or hospitalization for malignant hypertension
Hypotension (systolic) -mm Hg; at rest + calm	85 – 89	80 – 84	< 80	ER visit or hospitalization for hypotensive shock

Abbreviations: ER, emergency room.
[1] The definitions provided in the table are taken from the FDA Guidance for Industry "Toxicity Grading Scale for Healthy Adults and Adolescent Volunteers Enrolled in Preventive Vaccine Clinical Trials" dated September 2007.
[2] Subject should be at rest for all vital sign measurements.
[3] When resting heart rate is between 60 – 100 beats per minute. Use clinical judgment when characterizing bradycardia among some healthy subject populations, for example, conditioned athletes.

Laboratory AE Grading: Adults

Hematology and Biochemistry Values[1, 2]	Mild (Grade 1)	Moderate (Grade 2)	Severe (Grade 3)	Potentially Life Threatening (Grade 4)
Hemoglobin (Male) - gm/dL	9.5 – 10.3	8.0 – 9.4	6.5 – 7.9	< 6.5 and/or requiring transfusion
Hemoglobin (Female) - gm/dL	8.0 – 9.3	7.0 – 7.9	6.0 – 6.9	< 6 and/or requiring transfusion
WBC Increase - 10^3/µL	11.5 – 15.0	15.1 – 20.0	20.1 – 25.0	> 25.0
WBC Decrease - 10^3/µL	2.5 – 3.3	1.5 – 2.4	1.0 – 1.4	< 1.0 with fever
Neutrophil/Granulocyte Decrease[3] - 10^3/µL	0.80 – 0.90	0.50 – 0.79	< 0.50	< 0.50 with fever
Platelets Decreased - 10^3/µL	100 – 110	70 – 99	25 – 69	< 25
Creatinine (Male) - µmol/L	130.00 – 150.99	151.00 – 176.99	177.00 – 221.00	> 221.00 and requires dialysis
Creatinine (Female) - µmol/L	110.00 – 132.99	133.00 – 159.99	160.00 – 215.99	> 216.00 and requires dialysis
Liver Function Tests/ALT - U/L	75.0 – 150.9	151.0 – 300.9	301.0 – 600.0	> 600.0

Abbreviations: ALT, alanine transaminase; WBC, white blood cell.
[1] The laboratory values provided in the tables serve as guidelines and are dependent upon institutional normal parameters. Institutional normal reference ranges should be provided to demonstrate that they are appropriate.
[2] The clinical signs or symptoms associated with laboratory abnormalities might result in characterization of the laboratory abnormalities as Potentially Life Threatening (Grade 4). For example, a low sodium value that falls within a grade 3 parameter should be recorded as a grade 4 hyponatremia event if the subject had a new seizure associated with the low sodium value.
[3] Note, neutropenias are graded and followed, but based on previous experience in African populations, should be interpreted with caution since lower values are more frequently observed in people of African descent [29, 30].

15. REFERENCES

1. Ashley, E.A., et al., *Spread of artemisinin resistance in Plasmodium falciparum malaria.* N Engl J Med, 2014. **371**(5): p. 411-23.
2. The mal, E.R.A.C.G.o.V., *A Research Agenda for Malaria Eradication: Vaccines.* PLOS Medicine, 2011. **8**(1): p. e1000398.
3. 2019, W.H.O., *World malaria report 2019.* Geneva: World Health Organization; 2019. , 2019. **Licence:**
CC BY-NC-SA 3.0 IGO.
4. Gallup, J.L. and J.D. Sachs, *The economic burden of malaria.* Am J Trop Med Hyg, 2001. **64**(1-2 Suppl): p. 85-96.
5. Sauerwein, R.W. and T. Bousema, *Transmission blocking malaria vaccines: Assays and candidates in clinical development.* Vaccine, 2015. **33**(52): p. 7476-82.
6. Targett, G.A. and B.M. Greenwood, *Malaria vaccines and their potential role in the elimination of malaria.* Malaria Journal, 2008. **7**(1): p. S10.
7. Singh, S.K., et al., *Construct design, production, and characterization of Plasmodium falciparum 48/45 R0.6C subunit protein produced in Lactococcus lactis as candidate vaccine.* Microb Cell Fact, 2017. **16**(1): p. 97.
8. Bousema, T. and C. Drakeley, *Epidemiology and infectivity of Plasmodium falciparum and Plasmodium vivax gametocytes in relation to malaria control and elimination.* Clin Microbiol Rev, 2011. **24**(2): p. 377-410.
9. Blagborough, A.M., et al., *Transmission-blocking interventions eliminate malaria from laboratory populations.* Nature Communications, 2013. **4**(1): p. 1812.
10. Rabinovich, R.N., et al., *malERA: An updated research agenda for malaria elimination and eradication.* PLOS Medicine, 2017. **14**(11): p. e1002456.
11. Griffin, J.T., et al., *Reducing Plasmodium falciparum Malaria Transmission in Africa: A Model-Based Evaluation of Intervention Strategies.* PLOS Medicine, 2010. **7**(8): p. e1000324.
12. Singh, S.K., et al., *A Plasmodium falciparum 48/45 single epitope R0.6C subunit protein elicits high levels of transmission blocking antibodies.* Vaccine, 2015. **33**(16): p. 1981-6.
13. Magnusson, S.E., et al., *Immune enhancing properties of the novel Matrix-M adjuvant leads to potentiated immune responses to an influenza vaccine in mice.* Vaccine, 2013. **31**(13): p. 1725-33.
14. Reimer, J.M., et al., *Matrix-M adjuvant induces local recruitment, activation and maturation of central immune cells in absence of antigen.* PLoS One, 2012. **7**(7): p. e41451.
15. Magnusson, S.E., et al., *Matrix-M adjuvant enhances immunogenicity of both protein- and modified vaccinia virus Ankara-based influenza vaccines in mice.* Immunol Res, 2018. **66**(2): p. 224-233.
16. Carrasco, Y.R. and F.D. Batista, *B cells acquire particulate antigen in a macrophage-rich area at the boundary between the follicle and the subcapsular sinus of the lymph node.* Immunity, 2007. **27**(1): p. 160-71.
17. Gray, E.E. and J.G. Cyster, *Lymph node macrophages.* J Innate Immun, 2012. **4**(5-6): p. 424-36.
18. Bengtsson, K.L., et al., *Matrix-M adjuvant enhances antibody, cellular and protective immune responses of a Zaire Ebola/Makona virus glycoprotein (GP) nanoparticle vaccine in mice.* Vaccine, 2016. **34**(16): p. 1927-35.
19. Shinde, V., et al., *Induction of Cross-reactive Hemagglutination Inhibiting Antibody and Polyfunctional CD4+ T-cell Responses by a Recombinant Matrix-M-Adjuvanted Hemagglutinin Nanoparticle Influenza Vaccine.* Clin Infect Dis, 2020.
20. Bengtsson, K.L., et al., *Matrix-M adjuvant: enhancing immune responses by 'setting the stage' for the antigen.* Expert Rev Vaccines, 2013. **12**(8): p. 821-3.
21. Shinde, V., et al., *Improved Titers against Influenza Drift Variants with a Nanoparticle Vaccine.* N Engl J Med, 2018. **378**(24): p. 2346-2348.

22. Datoo, M.S., et al., *High efficacy of a low dose candidate malaria vaccine, R21 in 1 Adjuvant Matrix-M, with Seasonal Administration to Children in Burkina Faso.* Lancet, 2021.
23. Keech, C., et al., *Phase 1-2 Trial of a SARS-CoV-2 Recombinant Spike Protein Nanoparticle Vaccine.* N Engl J Med, 2020. **383**(24): p. 2320-2332.
24. Shinde, V., et al., *Efficacy of NVX-CoV2373 Covid-19 Vaccine against the B.1.351 Variant.* N Engl J Med, 2021. **384**(20): p. 1899-1909.
25. Shinde, V., et al., *Comparison of the Safety and Immunogenicity of a Novel Matrix-M-adjuvanted Nanoparticle Influenza Vaccine with a Quadrivalent Seasonal Influenza Vaccine in Older Adults: A Randomized Controlled Trial.* 2020: p. 2020.08.07.20170514.
26. Tapiainen, T. and U. Heininger, *Fever following immunization.* Expert Rev Vaccines, 2005. **4**(3): p. 419-27.
27. Singh, S.K., et al., *The Plasmodium falciparum circumsporozoite protein produced in Lactococcus lactis is pure and stable.* J Biol Chem, 2020. **295**(2): p. 403-414.
28. Theisen, M., et al., *A multi-stage malaria vaccine candidate targeting both transmission and asexual parasite life-cycle stages.* Vaccine, 2014. **32**(22): p. 2623-30.
29. Bain, B.J., *Ethnic and sex differences in the total and differential white cell count and platelet count.* J Clin Pathol, 1996. **49**(8): p. 664-6.
30. Haddy, T.B., S.R. Rana, and O. Castro, *Benign ethnic neutropenia: what is a normal absolute neutrophil count?* J Lab Clin Med, 1999. **133**(1): p. 15-22.

TBVax1 Clinical trial:

Phase 1 Dose Escalating, Double-Blind, Randomised Comparator Controlled Trial of the Safety and Immunogenicity of Different Adjuvant Formulations of R0.6C and ProC6C transmission blocking vaccines candidates against *Plasmodium falciparum* in Adults in Burkina Faso (TBVax1).

Pharmacy Manual
TBVax1 Clinical trial

R0.6C-AlOH and R0.6C-AlOH + Matrix-M1

ProC6C-AlOH and ProC6C-AlOH + Matrix-M1

Authors:
Name: J. Plieskatt
Date and Signature
22 September 2022

Review by:
Name: Bougouma Edith Christiane

Version 1.3 of 21 SEP 2022

Table of contents

1. TBVax1 study product formulation 2
2. Study Product Labels 2
3. Study product storage Conditions 3
4. Study product storage Monitoring 3
5. Study product doses and volumes for administration 4
   Fractioned dose R0.6C-AlOH 6
   Fractioned dose R0.6C-AlOH + Matrix-M1 6
   Fractioned dose ProC6C-AlOH 6
   Fractioned dose ProC6C-AlOH + Matrix-M1 6
   Full dose R0.6C-AlOH 6
   Full dose R0.6C-AlOH + Matrix-M1 6
   Full dose ProC6C-AlOH 6
   Full dose ProC6C-AlOH + Matrix-M1 6
6. Preparation of the study product for administration 8
7. Administration of prepared products 12
8. Accuracy of study product administration 13
9. Study product accountability 13
10. Study product disposition 14

1. TBVax1 study product formulation

The R0.6C-AlOH vaccine is composed of R0.6C recombinant protein (0.2 mg/ml of the recombinant protein) adsorbed to 1.6 mg/ml of Alhydrogel® as a refrigerated suspension (stored 2-8°C). The vaccine will be provided in a sterile 2 mL Type I glass vial containing 800 µL of suspension of white or grey particles in a colorless liquid. Each vial is designed for use in direct administration, or by admixing with Matrix-M1 added to the R0.6C-AlOH vial, for a single intramuscular dose.

The ProC6C-AlOH vaccine is composed of ProC6C recombinant protein (0.2 mg/ml of the recombinant protein) adsorbed to 1.6 mg/ml of Alhydrogel® as a refrigerated suspension (stored at 2-8°C). The vaccine will be provided in a sterile 2 mL Type I glass vial containing 800 µL of suspension of white or grey particles in a colorless liquid. Each vial is designed for use in direct administration, or by admixing with Matrix-M1 added to the ProC6C-AlOH vial, for a single intramuscular dose.

Matrix-M1 is supplied ready-to-mix as a refrigerated solution (stored 2-8°C) at a concentration of 0.375 mg/mL and a fill volume of 0.75 mL. The solution is clear and colorless.

2. Study Product Labels

Code de l'essai: TBVax1
10 flacons à usage unique

R0.6C-AlOH
(0.8 mL suspension stérile)
200 µg R0.6C (antigène) - 1.60 mg $Al(OH)_3$ (adjuvant)/mL
Vaccin reconstitué pour injection IM injection IM et pour reconstitution et injection IM.
Pour essai clinique uniquement
Préparer selon le Manuel de la Pharmacie
PI: Dr Alfred B. Tiono, Groupe de Recherche en Santé Publique (GRAS), BF, Tel: +226 7028 5726
Fabricant: Statens Serum Institut,
5 Artillerivej, 2300 Copenhagen S, Denmark
Conservé réfrigéré entre 2°C - 8° C
Numéro de lot: xxxxxx Utiliser avant: MM/YYYY

Code de l'essai: TBVax1
200 µg R0.6C - 1.60 mg $Al(OH)_3$/mL pour injection IM et pour reconstitution et injection IM. Usage unique selon le Manuel de la Pharmacie.
Pour essai clinique uniquement
Conservé réfrigéré entre 2°C - 8° C
PI: Dr Alfred B. Tiono

Numéro de lot: xxxxxx

Figure 1. Box label (left) and vial label (right) for R0.6C-AlOH

Code de l'essai: TBVax1
10 flacons à usage unique

ProC6C-AlOH
(0.8 mL suspension stérile)
200 µg ProC6C (antigène) - 1.60 mg $Al(OH)_3$ (adjuvant)/mL
Vaccin reconstitué pour injection IM
Pour essai clinique uniquement
Préparer selon le Manuel de la Pharmacie
PI: Dr Alfred B. Tiono, Groupe de Recherche en Santé Publique (GRAS), BF, Tel: +226 7028 5726
Fabricant: Statens Serum Institut,
5 Artillerivej, 2300 Copenhagen S, Denmark
Conservé réfrigéré entre 2°C - 8° C
Numéro de lot: xxxxxx Utiliser avant: MM/YYYY

Code de l'essai: TBVax1
200 µg ProC6C - 1.60 mg $Al(OH)_3$/mL pour injection IM et pour reconstitution et injection IM. Usage unique selon le Manuel de la Pharmacie.
Pour essai clinique uniquement
Conservé réfrigéré entre 2°C - 8° C
PI: Dr Alfred B. Tiono

Numéro de lot: xxxxxx

Figure 2. Box label (left) and vial label (right) for ProC6C-AlOH

Matrix-M1 Adjuvant 0.375 mg/mL
Pour essai clinique uniquement
Conservé réfrigéré entre 2°C - 8° C
Pour la reconstitution avec l'antigène et l'injection IM selon le Manuel de la Pharmacie
Expiration: 2022-05
Numéro de lot: M1-118
Fabricant: Novavax AB
PI: Dr Alfred B. Tiono , Groupe de Recherche en Santé Publique (GRAS), BF, Tel : +226 7028 5726
Code de l'essai: TBVax1

.75mL/flacon Art. nr:30-110-614 03
Matrix-M1 Adjuvant 0.375 mg/mL
Pour la reconstitution avec l'antigéne et l'injection IM
Expiration: 2022-05
Numéro de lot: M1-118 Fabricant: Novavax AB
PI: Dr Alfred B. Tiono
Code de l'essai: TBVax1

Figure 3. Box label (left) and vial label (right) for Matrix-M1 adjuvant

3. Study product storage Conditions

Vials of R0.6C-AlOH must be stored at 2ºC to 8ºC. Freezing destroys the integrity of aluminum hydroxide suspensions. Thus, any vials that have been frozen must not be used for administration to humans. **Vials should be stored in the upright position.**

Vials of ProC6C-AlOH must be stored at 2ºC to 8ºC. Freezing destroys the integrity of aluminum hydroxide suspensions. Thus, any vials that have been frozen must not be used for administration to humans. **Vials should be stored in the upright position.**

Vials of Matrix-M1 must also be stored at 2ºC to 8ºC. Any vials that have been frozen must not be used for administration to humans. **Vials should be stored in the upright position.**

4. Study product storage Monitoring

Vials of R0.6C-AlOH, ProC6C-AlOH, and Matrix-M1 must be stored at 2ºC to 8ºC and monitored regularly. The provided SSI Libero-logger must be placed together with the IP (IMP) (same shelf – same position). The temperature should be read at the beginning of each working day (before IP (IMP) is dispensed) and checked for alarms. Please note that the Libero Logger to be used must be placed in the refrigerator approximately 2 hours before the expected placement of the IP (IMP) to avoid alarm. The Libero Logger must first be started /activated when the IP is placed in the refrigerator.

How to use the Libero logger (Figure 4): The logger is started at the start/stop button and the display shows the actual temperature. Logging intervals are already pre-defined and there is a pre-defined start-up delay of 30 minutes. If the logger's shelf life (indicated on the actual logger) has exceeded or if the IP (IMP) is stored for more than 5 months the logger will need to be replaced. The trial site must have a system to keep track of when to replace the Libero loggers. When replaced with a new logger the start/stop button is pressed, and the USB interface is placed in a computer.

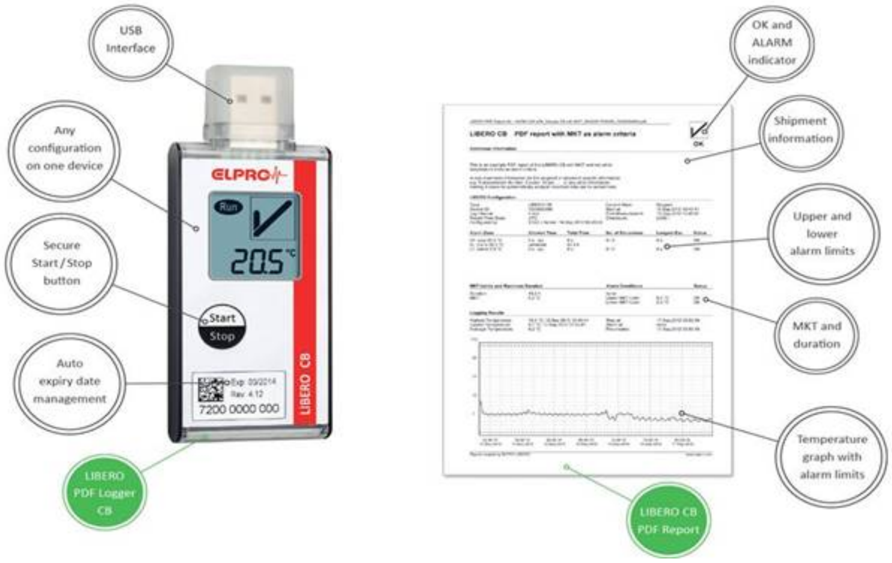

Figure 4. Libero temperature logger details

In case of refrigerator breakdown, alarm, power supply failure, etc: If there is a cold storage breakdown, temperature alarm, power supply failure, or similar the logger is stopped. The IP (IMP) is moved to a backup refrigerator and a new logger is placed together with the IP (IMP). If NO temperature excursion has been detected the alarm display on the Libero logger will display a **tick mark**: If a temperature excursion has been detected the alarm display on the Libero logger will display an **X mark**:

5. Study product doses and volumes for administration

For the clinical study the Malaria drug products R0.6C-AlOH and ProC6C-AlOH are administered intramuscularly. R0.6C-AlOH and ProC6C-AlOH are intended as a ready-to-use vaccines (study arms A or C) or further reconstituted on-site with Matrix-M1 (study arms B or D). The vaccine will first be administered in a fractioned dose to the sentinel groups (group 1). If considered safe, administration of the full dose is initiated (Group 2). See **table 1** for an overview of the study groups and corresponding vaccine and adjuvant doses.

Study arm	***Protein***	***Number of subjects***	***Number of vaccinations per subject***	***Candidate vaccine dose***	***Adjuvants***	***Volume R0.6C-AlOH in admin. dose***	***Volume Matrix-M1 in admin. dose***	***Total vol. to be admin.***
1A	*R0.6C*	*n=5*	*3*	*30µg R0.6C*	*Alhydrogel*	*150 µL*	*-*	*150 µL*
1B	*R0.6C*	*n=5*	*3*	*30µg R0.6C + 15µg Matrix-M1*	*Alhydrogel + Matrix-M1*	*150 µL*	*40 µL*	*190 µL*
1C	*ProC6C*	*n=5*	*3*	*30µg ProC6C*	*Alhydrogel*	*150 µL*	*-*	*150 µL*
1D	*ProC6C*	*n=5*	*3*	*30µg ProC6C + 15µg Matrix-M1*	*Alhydrogel + Matrix-M1*	*150 µL*	*40 µL*	*190 µL*
1E	*HepB*	*n=5*	*3*	*N/A*	*Aluminum hydroxide*	*N/A*	*N/A*	*1 mL (ENGERIX-B)*
2A	*R0.6C*	*n=20*	*3*	*100µg R0.6C*	*Alhydrogel*	*500 µL*	*-*	*500 µL*
2B	*R0.6C*	*n=20*	*3*	*100µg R0.6C + 49µg Matrix-M1*	*Alhydrogel + Matrix-M1*	*500 µL*	*130 µL*	*630 µL*
2C	*ProC6C*	*n=20*	*3*	*100µg ProC6C*	*Alhydrogel*	*500 µL*	*-*	*500 µL*
2D	*ProC6C*	*n=20*	*3*	*100µg ProC6C + 49µg Matrix-M1*	*Alhydrogel + Matrix-M1*	*500 µL*	*130 µL*	*630 µL*
2E	*HepB*	*n=20*	*3*	*N/A*	*Aluminum hydroxide*	*N/A*	*N/A*	*1 mL (ENGERIX-B)*

Table 1: TBVax1 study arms with corresponding vaccine dose and vaccine and adjuvant volumes for intramuscular administration.

Fractioned dose R0.6C-AlOH (**Group 1A**)

The fractioned dose R0.6C-AlOHfrac used for intramuscular administration is a final formulated vaccine ready to be used following aspiration of a volume of 150 µL from the original R0.6C-AlOH vial which corresponds to 30 µg of R0.6C (**Figure 5.)**

Fractioned dose R0.6C-AlOH + Matrix-M1 (**Group 1B**)

The fractioned dose R0.6C-AlOHfrac + Matrix-M1 used for intramuscular administration is composed of one vial R0.6C-AlOH which is reconstituted on-site with the addition of 210 µL Matrix-M1 to the R0.6C-AlOH vial. The reconstituted Trial Vaccine will be used immediately following aspiration of a volume of 190 µL (from the reconstituted vial) which corresponds to 30 µg of R0.6C and 15 µg of Matrix-M1. **(Figure 5.)**

Fractioned dose ProC6C-AlOH (**Group 1C**)

The fractioned dose ProC6C-AlOHfrac used for intramuscular administration is a final formulated vaccine ready to be used following aspiration of a volume of 150 µL from the original ProC6C-AlOH vial which corresponds to 30 µg of ProC6C. **(Figure 6.)**

Fractioned dose ProC6C-AlOH + Matrix-M1 (**Group 1D**)

The fractioned dose ProC6C-AlOHfrac + Matrix-M1 used for intramuscular administration is composed of one vial of ProC6C-AlOH which is reconstituted on-site with the addition of 210 µL Matrix-M1 to the ProC6C-AlOH vial. The reconstituted Trial Vaccine will be used immediately following aspiration of a volume of 190 µL (from the reconstituted vial) which corresponds to 30 µg of ProC6C and 15 µg of Matrix-M1. **(Figure 6.)**

Full dose R0.6C-AlOH (**Group 2A**)

The full dose of R0.6C-AlOH used for intramuscular administration is a final formulated vaccine ready to be used following aspiration of a volume of 500 µL from the original R0.6C-AlOH vial which corresponds to 100 µg of R0.6C. **(Figure 5.)**

Full dose R0.6C-AlOH + Matrix-M1 (**Group 2B**)

The full dose of R0.6C-AlOH + Matrix-M1 used for intramuscular administration is composed of one vial of R0.6C-AlOH which is reconstituted on-site with the addition of 210 µL Matrix-M1 to the R0.6C-AlOH vial. The reconstituted Trial Vaccine will be used immediately following aspiration of a volume of 630 µL (from the reconstituted vial) which corresponds to 100 µg of R0.6C and 49 µg of Matrix-M1. **(Figure 5.)**

Full dose ProC6C-AlOH (**Group 2C**)

The full dose of ProC6C-AlOH used for intramuscular administration is a final formulated vaccine ready to be used following aspiration of a volume of 500 µL from the original ProC6C-AlOH vial which corresponds to 100 µg of ProC6C. **(Figure 6.)**

Full dose ProC6C-AlOH + Matrix-M1 (**Group 2D)**

The full dose of ProC6C -AlOH + Matrix-M1 used for intramuscular administration is composed of one vial of ProC6C -AlOH which is reconstituted on-site with the addition of 210 µL Matrix-M1 to the ProC6C -AlOH vial. The reconstituted Trial Vaccine will be used immediately following aspiration of a volume of 630 µL (from the reconstituted vial) which corresponds to 100 µg of ProC6C and 49 µg of Matrix-M1. **(Figure 6.)**

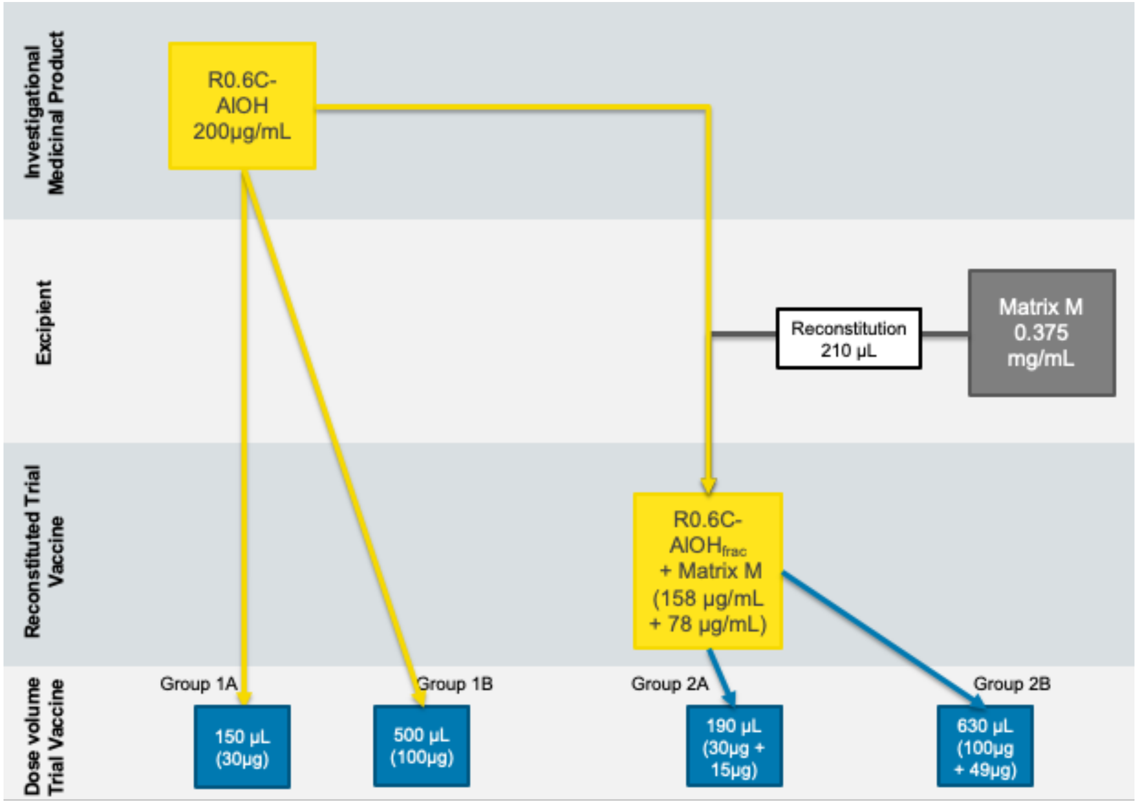

Figure 5. R0.6C-AlOH formulation manipulations with and without Matrix-M1 **(Groups A,B)**

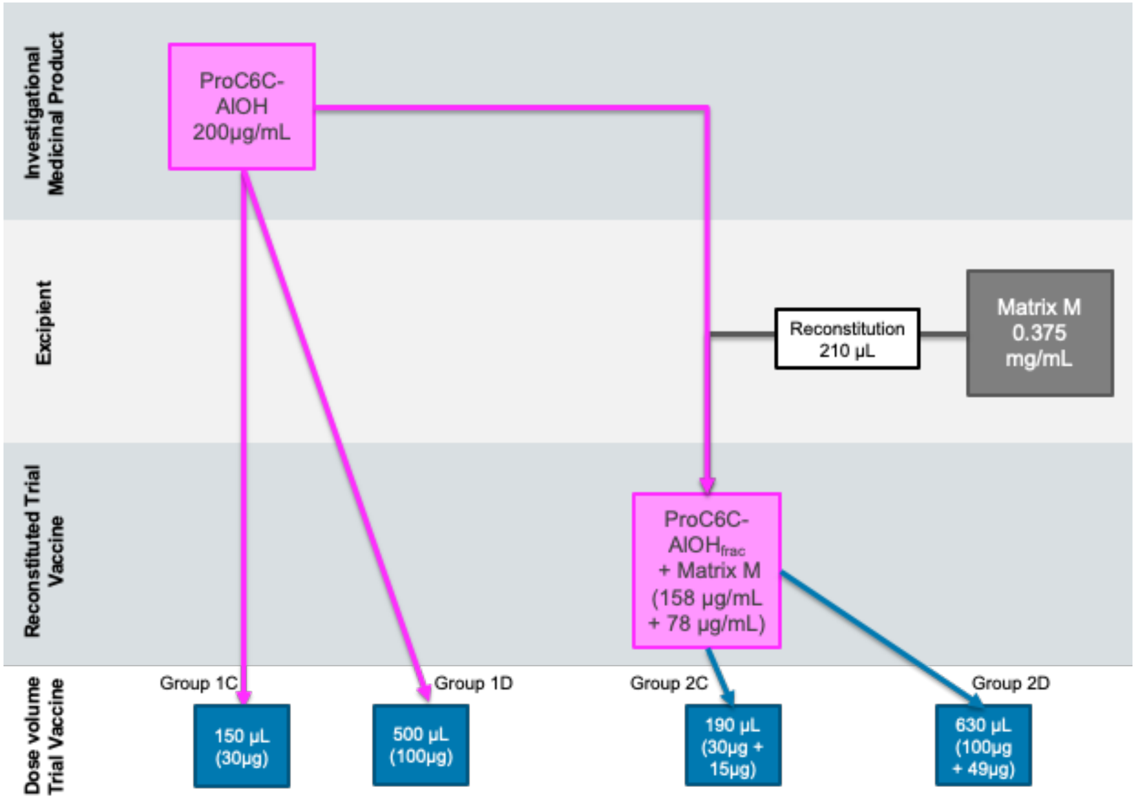

Figure 6. R0.6C-AlOH formulation manipulations with and without Matrix-M1 **(Groups C,D)**

6. Preparation of the study product for administration

This section describes the preparation and administration of the study product.

The following procedures apply to preparing the product for administration in **groups A(R0.6C)** and **groups C (ProC6C)** *without Matrix-M*.

1. Syringes and materials specified below are preferable, however like-like substitutions may be made if exact materials are unavailable. Product manipulations should occur with a syringe with volumetric indications of 0.010 mL. The needle gauge for administration should not be changed (25G) but length may be adjusted as required for material availability.
2. A prescription request must be sent to the pharmacy. A prescription request must be sent to the pharmacy, The prescription must contain the subject's participant code, Date of Prescription, Visit number and cohort. The participant code will be compared to the code assigned to the randomization envelope (randomization envelope prepared by the independent pharmacist, containing the relevant group, vaccine (the dose level of R0.6C or ProC6C, and whether Matrix-M is to be added as an additional adjuvant) or placebo).
3. The products as contained in sealed, sterile vials, do not require manipulation in a biological safety cabinet. Pharmacy manipulations may be performed at Room Temperature (20-30°C) "Benchside" at either GRAS or at the clinical trial site in the pharmacy.
4. The material should be kept refrigerated or at room temperature during the entire preparation period until use. If refrigerated, it is imperative that frozen cold packs are not used nor come in contact with the product – it is suggested to utilize cold packs equilibrated to 2-8°C.
5. Mix the contents of the vial with the study product R0.6C-AlOH or ProC6C-AlOH by inversion (10x) to ensure re-suspension and assure homogeneity before withdrawing into a syringe for administration. Do not shake the vial.
6. If the vial has settled for > 10 minutes, prior to withdrawing of the dose – the vial should be mixed by inversion (10x) to ensure resuspension. Use a 1mL luer-lok syringe (BD309628 or equivalent) and an appropriate needle for aspiration (e.g. 18G 1-1 ½ inch or equivalent) of the study product from the vial.
7. Aspirate the volume for administration (150 µL or 500 µL) plus approximately 100 µL overage. Register the time of aspiration from the vial into the syringe.
8. Replace the needle for aspiration with a needle suitable for intramuscular administration (e.g. 25G 1 ½ inch needle BD305127 or equivalent).
9. Remove the overage from the syringe.
10. The syringe should be labeled according to the GCP guidelines with the time since the drug product was prepared. The syringe should be allowed to equilibrate to room temperature before administration. Since this is a randomized trial, the Contents of syringes will be masked to keep blinded (syringes should be masked by opaque tape or other means). This is to ensure that the operator and the study subjects are unaware of the exact vaccine formulation i.e test vaccine versus control vaccine and volume.
11. The syringe should be inverted 10x by hand to assure homogeneity. This step can be skipped if there is <10 minutes between aspiration from the vial and intramuscular administration of the study product. The vaccine should be administered immediately after dispensing.

12. After completion of procedure steps, 1 to 11 the study product is ready for intramuscular administration in the deltoid muscle.
13. Any unused portion of a product vial will not be used for another subject and will be marked and retained per Product Disposition as described in section 10.

The following procedures apply to preparing the product for administration in **groups B (R0.6C)** and **groups D (ProC6C)** *with Matrix-M:*

1. Syringes and materials specified below are preferable, however like-like substitutions may be made if exact materials are unavailable. Product manipulations should occur with a syringe with volumetric indications of 0.010 mL. The needle gauge for administration should not be changed (25G) but length may be adjusted as required for material availability. For addition of Matrix-M to the study product (R0.6C-AlOH or ProC6C-AlOH) may be completed with a needle of any gauge or length.
2. A prescription request must be sent to the pharmacy. A prescription request must be sent to the pharmacy, the prescription must contain the subject's participant code, date of Prescription, Visit number and cohort. The participant code will be compared to the code assigned to the randomization envelope (randomization envelope prepared by the independent pharmacist, containing the relevant group, vaccine (the dose level of R0.6C or ProC6C, and whether Matrix-M is to be added as an additional adjuvant) or placebo).
3. The products as contained in sealed, sterile vials, do not require manipulation in a biological safety cabinet. Pharmacy manipulations may be performed at Room Temperature (20-30°C) "Benchside" at either GRAS or at the clinical trial site in the pharmacy.
4. The material should be kept refrigerated or at room temperature during the entire preparation period until use. If refrigerated, it is imperative that frozen cold packs are not used nor come in contact with the product – it is suggested to utilize cold packs equilibrated to 2-8°C.
5. Mix the contents of the vial with the adjuvant Matrix-M1 by inversion (10x) to ensure re-suspension and assure homogeneity. Do not shake the vial.
6. Use a 1mL luer-lok syringe (BD309628 or equivalent) and an appropriate needle for aspiration (e.g. 18G 1-1 ½ inch or equivalent) of the adjuvant Matrix-M1 from the vial. Aspirate 210 µL plus approximately 40 µL overage from the vial containing Matrix-M1 plus approximately 40 µL overage. from the vial containing Matrix-M1
7. Keep the rest of the contents of the Matrix-M1 vial, return the vial to storage at 2-8°C, for another product preparation (one vial of Matrix-M1 is sufficient to withdraw up to two doses of Matrix-M1).
8. Remove overage from the syringe and transfer the 210 µL of Matrix-M1 into the vial containing 800 µL of the study product R0.6C-AlOH or ProC6C-AlOH with the same needle as used in the previous step.
9. Mix the contents of the vial now containing the study product R0.6C-AlOH or ProC6C-AlOH and Matrix-M1 by inversion (10x) to ensure re-suspension and assure homogeneity before withdrawing into a syringe for administration. Do not shake the vial. Once the two components are mixed, the vaccine preparation should be immediately withdrawn into a syringe for administration. While the prepared product should be administered expeditiously, the products once mixed are stable for up to 8 hours at 2-8°C or Room Temperature (25°C). While stable, the products should be stored at 2-8°C whenever possible prior to dose withdrawal.

10. If the vial has settled for > 10 minutes, prior to withdrawing of the dose – the vial should be mixed by inversion (10x) to ensure resuspension. Use a new 1mL luer-lok syringe (BD309628 or equivalent) and new needle for aspiration of (R0.6C-AlOH + Matrix-M1) or (ProC6C-AlOH + Matrix-M1) from the vial.
11. Withdraw the volume for administration (190 µL or 630 µL) plus approximately 100 µL overage. Register the time of aspiration from the vial into the syringe.
12. Replace the needle for aspiration with a needle suitable for intramuscular administration (e.g. 25G 1 ½ inch needle BD305127 or equivalent).
13. Remove the overage from the syringe.
14. The syringe should be labeled with according to the GCP guidelines with the time since the drug product was prepared. The syringe should be allowed to equilibrate to room temperature before administration. Since his is a randomized trial, the Contents of syringes will be masked to keep blinded (syringes should be masked by opaque tape or other means). This is to ensure that the operator and the study subjects are unaware of the exact vaccine formulation i.e test vaccine versus control vaccine and volume.
15. The syringe should be inverted 10x by hand to assure homogeneity. This step can be skipped if there is <10 minutes between aspiration from the vial and intramuscular administration of the study product. The vaccine should be administered within immediately after dispensing.
16. After completion of procedure steps 1 to 15 the study product is ready for intramuscular administration in the deltoid muscle.
17. Any unused portion of a reconstituted product vial will not be used for another subject and will be marked and retained per Product Disposition as described in section 10. If prepared on the same day, the Matrix-M1 vial may be used for multiple R0.6C-AlOH or ProC6C-AlOH reconstitution preparations (e.g. Max of three 210 µL withdrawals of Matrix-M from a single vial for reconstitution is possible).

The following procedures apply to preparing the product for administration in **groups E (Hepatitis B, placebo)**

1. A prescription request must be sent to the pharmacy. . A prescription request must be sent to the pharmacy, The prescription must contain the subject's participant code, date of Prescription, Visit number and cohort. The participant code will be compared to the code assigned to the randomization envelope (randomization envelope prepared by the independent pharmacist, containing the relevant group, vaccine (the dose level of R0.6C or ProC6C, and whether Matrix-M is to be added as an additional adjuvant) or placebo).
2. The relevant formulation (respective to group and dose level) will then be prepared according to the assigned group. If a subject is assigned to the placebo group (Group 1E or Group 2E), the following preparation conditions apply.
3. The hepatitis B vaccine is prepared according to the package insert for the product. The placebo formulation is intended to be ENGERIX-B supplied in a single-dose vial or pre-filled syringed.
   For single-use vial: Shake well before use. With thorough agitation, ENGERIX-B is a homogeneous, turbid white suspension. Do not administer if it appears otherwise. Parenteral drug products should be inspected visually for particulate matter and discoloration prior to administration, whenever solution and container permit. If either of these conditions exists, the vaccine should not be administered. Withdraw 1 mL of the ENGERIX-B suspension

   For pre-filled syringe: Invert the syringe 10X by hand, verify the appropriate volume is present in the pre-filled syringe (e.g. 1 mL).
4. Replace the needle for aspiration with a needle suitable for intramuscular administration (e.g. 25G 1 ½ inch needle BD305127 or equivalent).
5. Remove the overage from the syringe.
6. The syringe should be labeled with according to the GCP guidelines with the time since the drug product was prepared. The syringe should be allowed to equilibrate to room temperature before administration. Since his is a randomized trial, the Contents of syringes will be masked to keep blinded (syringes should be masked by opaque tape or other means). . This is to ensure that the operator and the study subjects are unaware of the exact vaccine formulation i.e test vaccine versus control vaccine and volume.
7. The syringe should be inverted 10 x by hand to assure homogeneity. This step can be skipped if there is <10 minutes between aspiration from the vial and intramuscular administration of the study product. The vaccine should be administered within immediately after dispensing.

7. Administration of prepared products

The following procedures apply to the administration of the product:

1. Confirm identification and subject participant code of the subject prior to the administration.
2. Verify if the label on the syringe contains the correct participant code, Date of Prescription, Visit number, and cohort.
3. The syringe should be inverted 10x by hand to assure homogeneity. This step can be skipped if there is <10 minutes between aspiration from the vial and intramuscular administration of the study product.
4. Disinfect the injection site and administer the contents of the syringe in the deltoid muscle.

8. Accuracy of study product administration

The precision of the administered volume was estimated by withdrawing Milli-Q water following steps of the preparation procedures described in section 6. The weight (and thus volume) of the Milli-Q after ejection of the syringes was determined using a Sartorius Entris 90mm analytical balance (readability of 0.1 mg). Results are represented in **figure 7**. Deviations were below 10% for the low dose of 30µg (150µL and 190µL) and below 5% for the full dose of 100µg (500µL and 630µL).

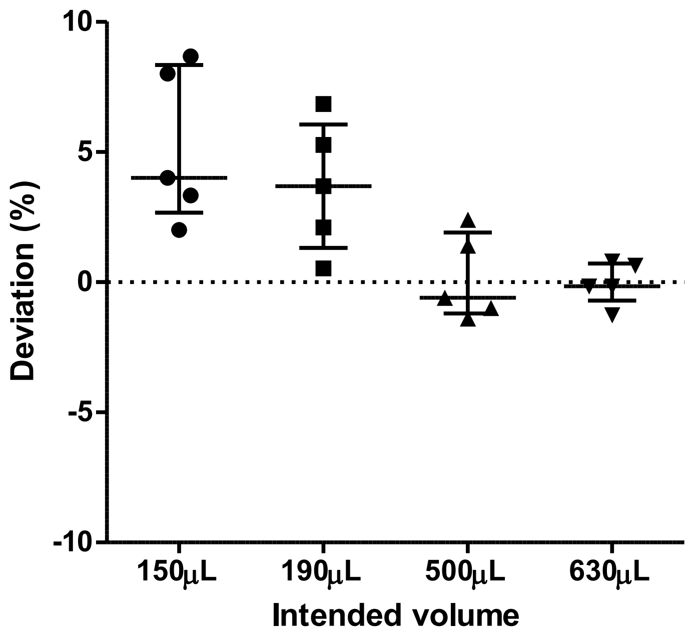

Figure 7. Accuracy of volumes for administration. Represented are the deviations in percentage (%) from the intended volume for administration. Error bars represent median and interquartile ranges.

9. Study product accountability

The study pharmacist will be responsible for maintaining complete records of TBVax1 study products including inventory and an accountability record of study agent supplies. Electronic documentation, as well as paper copies, may be used.

10. Study product disposition

The empty vials or unused portion of entered vials (R0.6C-AlOH, ProC6C-AlOH, or Matrix-M1) shall be retained and stored at 2-8°C until SSI provides a recommendation on disposal. Any unopened vials that remain at the end of the study will be returned to SSI or discarded at the discretion of SSI in accordance with policies that apply to investigational agents. Partially used reconstituted vials will not be administered to other subjects or used for in vitro experimental studies. These vials will be disposed of in accordance with institutional or pharmacy policy.
